# Identification of NETs and inflammation-related prognostic genes in breast cancer and PCR experimental validation

**DOI:** 10.3389/fgene.2026.1900298

**Published:** 2026-08-21

**Authors:** Zhihong Xu, Qian Ma, Qihua Jiang, Wensong Wei, Dan Tao

**Affiliations:** Department of Breast Surgery, Nanchang People’s Hospital, Nanchang, China

**Keywords:** breast cancer, inflammation, neutrophil extracellular traps, prognostic gene, prognostic model

## Abstract

**Background:**

Neutrophil extracellular trap-driven necrosis-related genes (NRGs) and inflammation-related genes (IRGs) are crucial in mitigating or inhibiting cancer progression in breast cancer patients. The interaction between NRGs and IRGs in breast cancer remains unclear. Thus, identifying prognostic genes associated with NETosis and inflammation in breast cancer may offer a novel approach to improving the outcome of breast cancer.

**Methods:**

Genes with statistically significant expression levels and correlations with IRGs and NRGs scores were selected as candidate genes, and the protein-protein interactions of the encoded proteins were explored. Subsequently, prognostic genes were further identified and build the risk model. Lastly, independent prognostic factors were determined through independent prognostic analysis, a prognostic model was established, and the immune microenvironment and drug sensitivity were analyzed.

**Results:**

The study identified *ZMYND10*, *IL12B*, *CXCL13*, *TFF1*, *LTB*, and *SPIB* as prognostic genes. Additionally, risk score and three clinical features, including age, were established as independent prognostic factors. A prognostic model with moderate predictive accuracy was developed. Further analysis revealed that six prognostic genes were considerably correlated with differential immune cells, among which *CXCL13*, *IL12B*, *LTB*, and *SPIB* were considerably positively correlated with most differential immune cells. Additionally, 18 drugs were considerably associated with the risk score, including six drugs such as Metformin and Thapsigargin, which could potentially be used to treat breast cancer.

**Conclusion:**

This study constructed a risk model and a nomogram for breast cancer prognosis using bioinformatics methods, and analyzed prognostic genes of breast cancer, which will be helpful to the improvement of breast cancer’s outcome and the development of clinical medicine.

## 1 Introduction

Breast cancer (BC) is the most commonly diagnosed cancer among women worldwide and the leading cause of cancer-related mortality in females. With over 2.4 million new cases recorded annually, its persistently high incidence and mortality rates pose a major global public health challenge (Xu and Wang, 2025). BC is a highly heterogeneous disease. Even patients with the same clinical stage and pathological grade can exhibit distinct responses to treatment and varying prognoses (Chen W. et al., 2024). The disease carries a high incidence rate, with tumor recurrence and distant metastasis serving as the primary causes of patient mortality. Approximately 5% of initially diagnosed cases already present with metastasis, while 20%–30% of patients with localized disease progress to metastatic BC (Sung et al., 2021). Despite current therapeutic approaches encompassing surgery, systemic therapy, and radiotherapy, tumor heterogeneity and drug resistance significantly limit clinical efficacy. Consequently, the prognosis for BC remains poor (Xiong et al., 2025). Therefore, there is an urgent need for molecular biomarkers to guide personalized treatments and improve overall prognosis.

Recent evidence indicates that neutrophils are crucial components of the tumor microenvironment and promote BC progression and metastasis (Liu et al., 2026; Obeagu, 2025). Studies demonstrate that the DNA components of Neutrophil Extracellular Traps (NETs) can mediate tumor cell metastasis via the CCDC25 receptor. Importantly, NET components are frequently detected in BC liver metastasis lesions. This suggests that serum NETs detection may serve as a predictive marker for early-stage liver metastasis (Martins-Cardoso et al., 2020). Xiao et al. found that tumor-secreted protease cathepsin C influences neutrophil recruitment and NET formation, promoting lung metastasis in BC (Xiao et al., 2021). Various studies have explored the relationship between NETs and BC from multiple angles. For example, Zhen et al. highlighted the critical role certain NET components play in enhancing the invasiveness of BC cells (Wang et al., 2020). While similar to the research by Yang et al. and Xiao et al., this study emphasizes the influence of NET components on cancer cell invasiveness, as opposed to Yang et al.’s focus on NETs’ role in liver metastasis and Xiao et al.’s focus on tumor-secreted proteases in NET formation and lung metastasis. Collectively, these findings suggest that NETs may serve as a strategic therapeutic target for blocking BC metastasis. However, a deeper exploration of their functional basis is still required. In addition to the crucial role of NETs in facilitating metastasis, the inflammatory state within the tumor microenvironment is recognized as another key driver of BC progression.

In recent years, an increasing number of studies have constructed prognostic models for breast cancer based on NETs-related genes or inflammation-related genes. Huang et al. identified four NETs-related biomarkers (F2RL2, AZU1, IL33, ELANE) (Huang et al., 2025) and established a NETs-based prognostic model; they also developed a three-gene NET-associated risk score specifically for triple-negative breast cancer (Huang et al., 2024). Another recent study reported a clinically validated NETosis-related immune signature panel capable of predicting postoperative prognosis (Wang and Da, 2026). With regard to inflammation, Wang et al. built a risk signature model incorporating 13 inflammation-related genes and developed multiple inflammatory response-related signature panels for specific breast cancer subtypes (Wang et al., 2025). All existing models share a common limitation: they only investigate NETs or inflammation as a single biological axis and fail to systematically integrate these two closely interconnected pathological processes. Despite these advances, there remains a substantial knowledge gap regarding systematic research on the synergistic mechanisms between NRGs and IRGs in BC.

To elucidate the molecular mechanisms underlying the biological functions of NETs in breast cancer, we constructed a necrosis-related gene set (NRGs) based on published literatures (Wu et al., 2022). Despite being termed “necrosis-related”, this gene set comprehensively covers multi-layer biomolecules associated with NETs, including core effector molecules of NETosis, upstream regulatory signals governing neutrophil activation, molecules mediating NETs-induced remodeling of the tumor microenvironment, and executors of necrotic programmed cell death. The selection of this gene set aims to capture a broad spectrum of NETs-relevant biological processes, encompassing NETosis execution molecules, upstream regulators of neutrophil activation, mediators of NETs-triggered microenvironmental remodeling, and modulators of inflammatory cell death pathways (Shi et al., 2020; Shahzad et al., 2025; Wu et al., 2025). Accordingly, the NRG set enables integrated characterization of NETs-associated biological behaviors in tumors.

Despite these advances, there remains a major knowledge gap concerning the systematic investigation of the synergistic mechanisms between NRGs and IRGs in BC. Current studies predominantly focus on the individual functions of either NRGs or IRGs, with a lack of comprehensive analysis of their synergistic effects. Prognostic models integrating both NRGs and IRGs, as well as their corresponding drug sensitivity analyses, have rarely been explored. This deficiency severely hinders the development of targeted therapeutic strategies that can simultaneously target NETosis and inflammatory pathways.

Given the critical roles of NETs and inflammation in breast cancer prognosis and the poorly elucidated synergistic mechanisms underlying their actions, the present study adopted the following research strategies. Consistent with the study conducted by Li Y. et al. (2023), we integrated transcriptomic data from The Cancer Genome Atlas (TCGA) and Gene Expression Omnibus (GEO) datasets. Based on public breast cancer transcriptomic data, bioinformatic approaches were utilized to explore the biological functions of NRGs and IRGs in breast cancer, screen prognosis-related genes associated with NETs and inflammation, and perform drug sensitivity prediction and analysis. Reverse transcription quantitative polymerase chain reaction (RT-qPCR) assays were further performed to verify the differential expression levels of these candidate prognostic genes between breast cancer tissues and normal control tissues.

Notably, we constructed a novel prognostic risk model integrating NETosis and inflammation-related signaling pathways, providing a dual-dimensional perspective for tumor biological research. This study aims to identify novel therapeutic targets for the prevention and clinical treatment of breast cancer, and propose innovative strategies to improve patient prognosis and advance the development of clinical breast cancer treatment.

## 2 Materials and procedures

### 2.1 Collection of data

Supplementary Figure S1 illustrates the workflow of this study. This research utilized the GDC-TCGA-BRCA dataset from the Cancer Genome Atlas (TCGA) database (https://xenabrowser.net/) as the training set (referred to as the TCGA-BRCA training set, 6 September 2024). The RNA sequencing data of TCGA - BRCA (including log2 (count+1) and log2 (bp-km+1) values) were downloaded from the UCSC Xena database (https://xena.ucsc.edu/). During the downstream differential expression analysis, the expression values need to be converted from the original count form to integer form using formula 2^(count−1), and then input into the DESeq2 software for analysis. Eventually, 19,712 genes from 1,104 tumor samples and 113 adjacent normal tissue samples were covered, among which 1,082 tumor samples had survival data. The GSE42568 and GSE20685 datasets (GPL570 platform) were retrieved from the Gene Expression Omnibus (GEO) database (https://www.ncbi.nlm.nih.gov/geo/). GSE42568 contains 104 BC tumor tissue samples with associated survival data, and GSE20685 includes 327 tumor tissue samples with associated survival data. Both datasets were processed in Series Matrix File format and had undergone background correction and quantile normalization using the RMA algorithm by the GEO database; expression values were therefore already log2-transformed. Probe IDs were mapped to gene symbols using the idmap function from the AnnoProbe package. Where multiple probes mapped to the same gene, the probe with the highest mean expression was retained, yielding 15,294 genes for downstream analysis. A total of 200 inflammation-related genes (IRGs) were obtained from the Molecular Signatures Database (MSigDB, v2023.2; https://www.gsea-msigdb.org/gsea/msigdb/), specifically from the HALLMARK_INFLAMMATORY_RESPONSE gene set (accession ID: M5933; H: hallmark gene sets collection) (Supplementary Table S1). The NRG list comprising 136 genes was curated from a previously published study (Wu et al., 2022), (Supplementary Table S2). Although this gene set extends beyond classical NETosis effectors, it was deliberately selected to capture the broader spectrum of NET-associated biology, encompassing NETosis execution molecules (e.g., *PADI4, MPO, ELANE*), upstream neutrophil activation regulators (e.g., *TLR2/4, NLRP3*), NET-associated microenvironmental remodeling mediators (e.g., *HMGB1, MMP9*), and inflammatory cell death pathway regulators (e.g., *RIPK1, GSDMD*).

To validate that the NRG list effectively reflects NET-associated biological activity in breast cancer, we obtained an established NETosis gene signature from the MSigDB C7 immunologic gene sets (GSE37416_0H_VS_24H_F_TULARENSIS_LVS_NEUTROPHIL_UP; 195 genes) (MSigDB v2023.2), which was derived from a neutrophil activation experiment and represents core NETosis-associated transcriptional changes. Single-sample gene set enrichment analysis (ssGSEA) was performed on TCGA-BRCA tumor samples using the “GSVA R” package (v1.5.0) (Hänzelmann et al., 2013) to independently score each sample for both the NRG list and the NETosis signature. Spearman rank correlation was then calculated between the two ssGSEA score vectors across all tumor samples, with statistical significance defined as P < 0.05.

### 2.2 Data preprocessing and quality control

For the TCGA-BRCA training set, low-expression genes were removed using the criterion of counts >1 in at least one sample, excluding 207 genes and retaining 19,505 genes for downstream analyses. No additional batch correction was applied to the TCGA dataset, as detailed batch metadata were unavailable and the DESeq2 internal normalization procedure accounts for library size and technical variation between samples. For the GEO datasets, following probe-to-gene mapping and log2 (x+1) transformation as described in Section 2.1, expression values were used directly for risk score calculation without further normalization. For cross-platform risk score validation, the risk score formula derived from the TCGA training set was applied directly to GEO microarray expression values without additional transformation. Given that risk scores are computed as a linear combination of gene expression values weighted by regression coefficients, the directional relationship between expression levels and risk stratification remains biologically consistent across platforms. The validity of this approach is supported by the significant risk group separation observed in the GSE42568 validation cohort (P = 0.044).

Clinical variables were obtained from the GDC database for 1,082 tumor samples with complete survival information. Samples with missing clinical data were excluded from clinical correlation analyses. Continuous variables were dichotomized where appropriate: age was categorized as ≤60 vs. >60 years; pathological stage as stage I–II vs. III–IV; T stage as T1–2 vs. T3–4; N stage as N0–1 vs. N2–3; and M stage as M0 vs. M1. All categorical variables were encoded as factors prior to analysis.

### 2.3 Differential expression of TCGA-BRCA dataset

The differential expression analysis was based on the original count data of the TCGA-BRAIN training set and was completed using the DESeq2 package (v 1.38.3) (Love et al., 2014). The tumor samples were set as the experimental group and the adjacent normal tissues as the control group. Using the default parameters, genes that met |log_2_FC| > 2 and a corrected P-value <0.05 were defined as significantly differentially expressed genes (DEGs). This analysis directly operated on the standardized integer count data, while all subsequent downstream analyses used the expression values after global log2 transformation. To present the results, volcano plots and heatmaps were drawn respectively using ggplot2 (v 3.4.1) and pheatmap (v 1.0.12) (Hu, 2021), and the top ten DEGs with the highest and lowest expression rankings were highlighted in descending order of |log2FC (Fold Change)|.

### 2.4 Survival analysis

To assess the impact of IRGs and NRGs on the survival of patients with BC, ssGSEA from the “GSVA” package (v 1.46.0) (Hänzelmann et al., 2013) was used to compute scores for all 1,082 samples in the TCGA-BRCA training set. Based on the optimal cutoff values for IRG and NRG scores, patients were classified into high-risk groups (HRG) and low-risk groups (LRG). Kaplan-Meier survival analysis for both groups was conducted using the “survival” package (v 3.3-1) (Gan et al., 2024). A P-value <0.05 indicated a significant difference in survival between the groups. All log-rank tests were two-sided, with statistical significance defined as P < 0.05.

### 2.5 Genetic co-expression network analysis

Gene modules related to IRGs and NRGs scores were identified through weighted gene co-expression network analysis (WGCNA) using the “WGCNA” package (v 1.72-5) (Langfelder and Horvath, 2008) on samples of patients with BC from the TCGA-BRCA training set. Hierarchical clustering with Euclidean distance was conducted on gene expression data from 1,082 TCGA-BRCA BC samples to identify and exclude outliers. The optimal soft threshold (β) for approximating a scale-free gene interaction network was determined using the “pickSoftThreshold” function. Gene modules were identified by applying the dynamic tree cutting algorithm with minModuleSize = 100 and mergeCutHeight = 0.1. IRG and NRG scores were used as phenotypic traits for Pearson correlation analysis between gene modules and traits, using the “psych” package, with a threshold of |r| > 0.3 and P < 0.05. (v 2.4.3) (Zein and Akhtar, 2025). The two modules with the highest correlations were designated as key modules, and genes within these modules were identified as key module genes.

### 2.6 Candidate factors and their enrichment analysis

Candidate genes for downstream analysis were identified as the common genes between DEGs and key module genes using the “VennDiagram” package (v 1.7.3) (Chen and Boutros, 2011). To explore the biological pathways and functions of these genes, Gene Ontology (GO) and Kyoto Encyclopedia of Genes and Genomes (KEGG) pathway enrichment analyses were performed using the “clusterProfiler” package (v 4.7.1.3) (Wu et al., 2021) and the “org.Hs.eg.db” package (v 3.15.0) (Ji et al., 2024). A Benjamini–Hochberg FDR-adjusted P < 0.05 was considered statistically significant. The top five significant GO terms and top 10 KEGG enrichment results were visualized using the “circlize” package (v 0.4.15) (Liu et al., 2024). Protein-protein interactions (PPIs) among proteins encoded by candidate genes were predicted using the STRING database (https://string-db.org/) with a confidence score of at least 0.4. Key nodes were identified by eliminating isolated, non-interacting proteins, and the results were visualized using Cytoscape software (v 3.7.2) (Lu et al., 2022).

### 2.7 Prognostic gene screening and risk model construction

A univariate Cox regression analysis was performed on 1,082 BC samples using the “survival” package to examine the association between candidate genes and BC prognosis. Genes with P < 0.05 and HR ≠ 1 were selected for the proportional risk assumption test (P > 0.05) to identify potential prognostic genes. The “glmnet” package (v 4.1.4) (Engebretsen and Bohlin, 2019) was used to perform LASSO-Cox proportionalrisks regression (family = “cox”) with L1 penalization on candidate genes. The optimal penalization parameter λ was selected *via* 10-fold cross-validation as the value minimizing partial likelihood deviance (λ.min = 0.004049102). These prognostic genes were subsequently used to construct a risk model, and risk scores were calculated for BC samples in TCGA-BRCA. The risk score was calculated using the following formula:

$$\text{risk score}=\sum_{i=1}^{n}\;\left(\text{coefi}*\text{xi}\right)$$

In this formula, “Coef” represents the coefficient of prognostic genes derived from LASSO analysis, while “expression” refers to their corresponding expression levels. The 1,082 patients with BC with complete survival data were classified into HRG and LRG. For external validation cohorts, the same cutoff was applied directly without re-optimization. The “ggplot2” package was used to visualize the distribution of risk score and survival statuses, while the “pheatmap” package was employed to generate a heatmap of prognostic gene expression in both HRG and LRG groups. Survival differences between HRG and LRG in the TCGA-BRCA training set were assessed using the “survminer” package (v 0.4.9) (Li et al., 2025), with statistically significant results (P < 0.05). ROC curves for various years were plotted using the “survivalROC” package (v 1.0.3.1) (Beyene et al., 2024) to evaluate the accuracy and precision of the risk model, where an AUC >0.6 indicated strong predictive performance. The model’s predictive capacity was further validated on the GSE42568 and GSE20685 datasets using the same criteria to assess its effectiveness in the validation set. To evaluate model optimism and internal validity, bootstrap resampling (n = 1,000 iterations) was performed using the “rms” package to calculate the optimism-corrected C-index.

### 2.8 Determination of independent prognosis factors and nomograph construction

To identify factors closely linked to prognosis, univariate Cox regression analysis was conducted on six clinical variables and risk score in the TCGA-BRCA training set. Variables with HR ≠ 1 and P < 0.05 were selected for the proportionalrisks assumption test using the cox.zph function. Variables satisfying the PH assumption (P > 0.05) were entered into multivariate Cox regression analysis. For variables violating the PH assumption (Stage, P = 0.0014; T.stage, P = 0.0054), a stratified Cox model was applied, incorporating them as stratification variables to allow stratum-specific baselinerisk functions. All categorical variables were encoded using dummy coding: age was dichotomized as ≤60 vs. >60 years, Stage as I, II, and III/IV, T.stage as T1, T2, and T3/4, N.stage as N0, N1, N2, and N3, and M.stage as M0 vs. M1. A nomogram was constructed based on independent prognostic factors identified from the stratified multivariable Cox model to predict the survival probability of patients with BC, using the “rms” package (v 6.5.0) (Li M. et al., 2023). The reliability of the nomogram was evaluated using the “rms”, “survivalROC”, and “ggDCA” (v 1.2) packages (https://cran-e.com/package/ggDCA). To assess the robustness of the risk model across clinically relevant subgroups, stratified Cox regression analyses were performed in subgroups defined by molecular subtype (ER, PR, HER2 status) and clinical stage (Stage I–II and Stage III–IV) using GSE42568. Risk ratios and 95% confidence intervals were estimated for each subgroup, and results were visualized as a forest plot.

### 2.9 Clinical feature analysis

The clinical value of the risk model was assessed by comparing risk score for age, gender, clinical stage, and other factors between HRG and LRG using the Wilcoxon test, with a P value <0.05 considered indicative of significant differences. Furthermore, the expression levels of prognostic genes were analyzed across various clinical characteristics, including age and gender, using the Wilcoxon test.

### 2.10 Analysis of immune infiltration

The ssGSEA algorithm was utilized to determine the infiltration levels of 28 immune cell types in both HRG and LRG. The ssGSEA immune cell gene signature set used in this study was derived from the research of Charoentong et al. This document contains 28 immune cell-related gene sets, covering various immune cell types such as T cells, B cells, macrophages, dendritic cells, and other key immune subgroups, as well as their specific expression profiles (Valdez et al., 2016). The Wilcoxon rank-sum test was employed to compare immune cell infiltration levels between HRG and LRG, with multiple comparisons corrected using the Benjamini–Hochberg method; both P-value and adjusted P are reported. The median difference (median_diff) between groups was calculated as a measure of effect size. Correlations between prognostic genes and differentially infiltrated immune cells were examined using the “psych” package, with Spearman correlation coefficients reported as effect sizes and a threshold of |r| > 0.3 and adjusted P < 0.05. To validate the robustness of the ssGSEA results, CIBERSORT algorithm was independently applied to estimate immune cell infiltration abundance between HRG and LRG. The CIBERSORT analysis employs the LM22 gene feature matrix established by Newman et al., which comprises 547 genes and can distinguish 22 types of human hematopoietic cell phenotypes (Banin et al., 2014).To further explore the association between the risk score and NET activity, Spearman correlations between the risk score and NET hallmark genes (MPO, ELANE, and PADI4) were calculated, with adjusted P < 0.05 considered statistically significant.

### 2.11 Enrichment of genetic groups and assay of tissue specificity

Gene expression differences between HRG and LRG in TCGA-BRCA patient samples were analyzed using the “DESeq2” package. DEGs were ranked by log_2_FC, and the associated signaling pathways and biological mechanisms were investigated. The gene list was compared with the “c2.cp.kegg.v2023.2.symbols.gmt” gene set from the MSigDB database (https://www.gsea-msigdb.org/gsea/msigdb) for Gene Set Enrichment Analysis (GSEA), using the “clusterProfiler” package (Wu et al., 2021), with criteria of adjust.P < 0.05 and |NES| > 1. The top five significantly enriched results were visualized using the “enrichplot” package (v1.18.0) (Zhai et al., 2023). The Genotype-Tissue Expression (GTEx) database (https://www.gtexportal.org/home/) was used for tissue-specific analyses of prognostic genes.

### 2.12 Molecular regulatory network, drug sensitivity and immunotherapy response analysis

Prognostic microRNAs (miRNAs) were predicted using the TargetScan database (https://targetscan.org/), and potential long non-coding RNAs (lncRNAs) interacting with these miRNAs were identified using the starBase database (http://starbase.sysu.edu.cn/) with a threshold of clipExpNum >15. A network linking lncRNA, miRNA, and mRNA was constructed using the “ggsankey” package (v 0.0.999) (https://github.com/davidsjoberg/ggsankey), illustrating the molecular regulatory mechanisms of the prognostic genes. Chemotherapy drug sensitivity data were obtained from the Cancer Drug Sensitivity Genomics database (https://ngdc.cncb.ac.cn/databasecommons/database/id/419), which included 138 drugs in TCGA-BRCA samples. The half-maximal inhibitory concentration (IC50) for each drug was calculated using the “pRRophetic” package (v0.5) (Geeleher et al., 2014). The correlation between the risk score and drug IC50 was assessed using Spearman rank correlation, with Spearman r reported as the effect size and multiple comparisons corrected using the Benjamini–Hochberg method (adjusted P < 0.05, |r| > 0.3). Differences in IC50 between HRG and LRG were compared using the Wilcoxon rank-sum test, with median difference (median_diff) reported as the effect size and BH correction applied for multiple comparisons. A violin plot was generated using the “pRRophetic” package (v 0.5) to visualize the top six drugs ranked by P-value.

To evaluate the potential response to immune checkpoint inhibitor therapy, tumor immune dysfunction and exclusion (TIDE) scores, immune exclusion scores, and dysfunction scores were computed for each patient using the TIDE platform (http://tide.dfci.harvard.edu). Differences in these three scores between the high- and low-risk groups were analyzed using the Wilcoxon rank-sum test, with P < 0.05 considered statistically significant. A higher TIDE score generally predicts a poorer response to immunotherapy and shorter overall survival.

### 2.13 Reverse transcription quantitative PCR (RT-qPCR)

Ethical approval was granted by the Third Hospital of Nanchang. All participants signed informed consent forms, with the ethics review document number K-ky2023041. RT-qPCR validation was performed on five paired tissue samples, each pair consisting of breast cancer tumor tissue and adjacent non-tumor breast tissue from the same patient. Patients ranged in age from 47 to 72 years (median: 56.2 years). Among them, three cases were stage II, one was stage I, and one was stage III. One patient was diagnosed with triple-negative breast cancer, while the remaining patients had luminal-type breast cancer. Total RNA was extracted from 50 mg of each frozen tissue sample using TRIzol reagent (Ambion, United States) following the manufacturer’s protocol. RNA concentration and purity were measured using the NanoPhotometer N50. RNA concentrations ranged from 705.44 to 992.56 ng/μL for control samples and from 220.32 to 2,989.30 ng/μL for tumor samples. A260/A280 ratios ranged from 1.300 to 1.923 (controls) and 1.447 to 1.703 (tumor samples). Although A260/A230 ratios were below the conventional standard (range: 0.156–1.171), primer specificity was rigorously confirmed by (i) designing primers according to established principles with validation via NCBI Primer-BLAST to ensure exclusive target gene binding, and (ii) performing melt curve analysis after amplification, which revealed a single, sharp peak for all gene targets, confirming the absence of non-specific amplification or primer dimer formation. Detailed RNA quality metrics for all samples are provided in Supplementary Table S4. The melting curve data are presented in Supplementary Figure S2.

mRNA was reverse-transcribed into cDNA using the Hifair® III 1st Strand cDNA Synthesis SuperMix kit (Yeasen, China) with 2 μg total RNA per reaction. RT-qPCR was performed using 2× Universal Blue SYBR Green qPCR Master Mix on a BIO-RAD CFX Connect real-time PCR system. Each 10 μL reaction consisted of 3 μL cDNA, 5 μL master mix, 1 μL forward primer (10 μM), and 1 μL reverse primer (10 μM). Thermal cycling conditions were: pre-denaturation at 95 °C for 1 min; 40 cycles of denaturation at 95 °C for 20 s, annealing at 55 °C for 20 s, and extension at 72 °C for 30 s. Each biological sample was run in technical triplicate, with reaction compositions, thermal cycling conditions, and primer sequences provided in Supplementary Tables S3,S4.

Of the six identified prognostic genes, five (ZMYND10, IL12B, CXCL13, TFF1, and SPIB) were subjected to RT-qPCR validation. LTB was not included in the experimental validation, as the available tissue volume from each patient was inherently constrained by the clinical sampling protocol conducted under the existing ethical approval framework (K-ky2023041); additional tissue collection solely for research validation purposes was not ethically permissible within the scope of this study. GAPDH served as the internal reference gene for normalization. Relative expression levels were calculated using the 2^−ΔΔCT^ method. Expression differences between tumor and control samples were assessed using an paired t-test (P < 0.05 considered statistically significant).

### 2.14 Statistical analysis

All data were processed using R software (v 4.2.3) (Peng et al., 2024). Group comparisons were conducted using the Wilcoxon rank-sum test (two-sided). Survival differences between groups were assessed using the log-rank test (two-sided). Cox regression analyses are reported as risk sorce with 95% CI. Spearman rank correlation (two-sided) was used for correlation analyses, with correlation coefficients and 95% CI reported as effect sizes. For analyses involving multiple comparisons, P-values were corrected using the Benjamini–Hochberg method and reported as FDR-adjusted P. Exact P-values are reported where feasible.

## 3 Results

### 3.1 1,819 genes identified as DEGs

**FIGURE 1:**
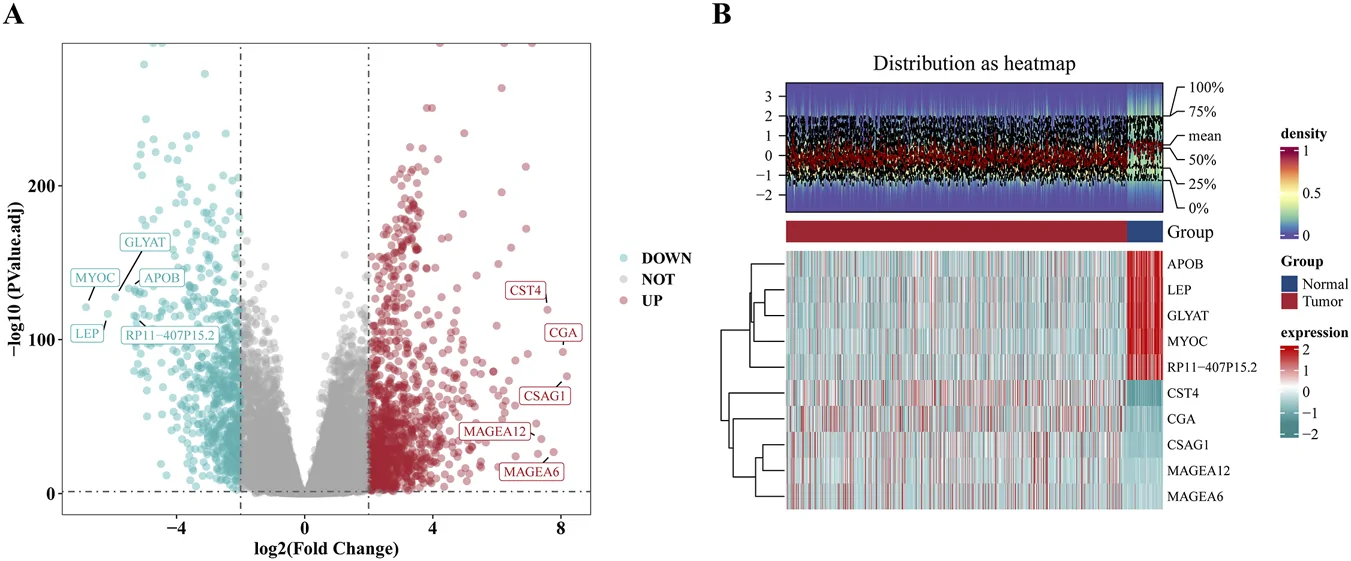
Identification of DEGs in TCGA-BRCA. **(A)** Volcano plot of transcriptomic comparison between BC tumor samples and normal breast tissue. Upregulated genes and downregulated genes met thresholds of |log_2_FC| > 2 and p.adj <0.05 (Benjamini–Hochberg correction). **(B)** Heatmap of the top 10 upregulated and top 10 downregulated DEGs (ranked by |log_2_FC|) across tumor and normal samples. Color scale represents z-scored expression; hierarchical clustering applied to rows and columns.

Prior to downstream analyses, we validated the biological relevance of the NRG list in breast cancer. ssGSEA scoring in TCGA-BRCA tumor samples demonstrated a strong positive correlation between NRG scores and NETosis signature scores (Spearman r = 0.702, P < 0.001; Supplementary Figure S3A), supporting the capacity of the NRG list to capture NET-associated biological activity in the breast cancer tumor microenvironment. A total of 1,819 DEGs (FDR-adjusted P < 0.05, |log_2_FC| > 2) were identified between tumor and normal groups in TCGA-BRCA, including 1,127 upregulated genes and 692 downregulated genes in BC (Figure 1A). Additionally, the heatmap of DEGs showed distinct expression patterns between tumor and normal samples, with the top 10 upregulated and downregulated genes (ranked by |log_2_FC|) showing clear separation between the two groups (Figure 1B).

### 3.2 Survival gaps among high and low scores

**FIGURE 2:**
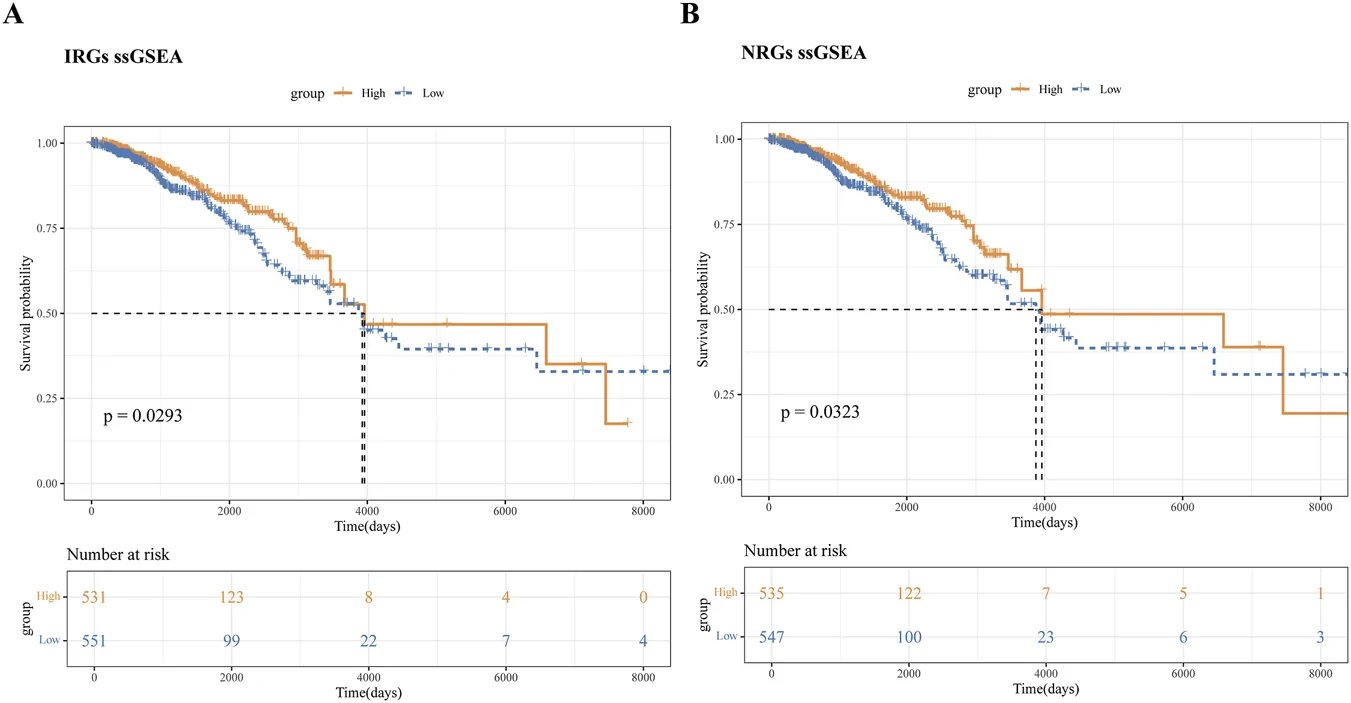
K-M survival curves based on ssGSEA scores of IRGs and NRGs in BC patients. **(A)** KM curves for IRG ssGSEA score groups (optimal cutoff = 0.4001984; HRG n = 531, LRG n = 551; log-rank P = 0.0293). At-risk numbers at 0/2,000/4,000 days: HRG 531/123/6, LRG 551/99/22. **(B)** KM curves for NRG ssGSEA score groups (optimal cutoff = 0.6991292; HRG n = 535, LRG n = 547; log-rank P = 0.0323). At-risk numbers at 0/2,000/4,000 days: HRG 535/122/6, LRG 547/100/25. Optimal cutoffs determined by maximally selected rank statistics.

To stratify the 1,082 BC patients by IRG and NRG activity, ssGSEA scores were calculated and optimal cutoffs were determined by maximally selected rank statistics. For IRGs, a cutoff of 0.4001984 classified 531 patients into the HRG and 551 into the LRG; KM analysis showed significantly longer OS in the HRG versus the LRG (log-rank test, P = 0.0293; Figure 2A), with the HRG maintaining a survival probability of ∼0.50 at ∼4,000 days while the LRG declined below this threshold earlier. For NRGs, a cutoff of 0.6991292 classified 535 patients into the HRG and 547 into the LRG; similarly, the HRG demonstrated significantly longer OS than the LRG (log-rank test, P = 0.0323; HR = 0.72, 95% CI: 0.52–0.99; Figure 2B).

### 3.3 Identification of key module genes

**FIGURE 3:**
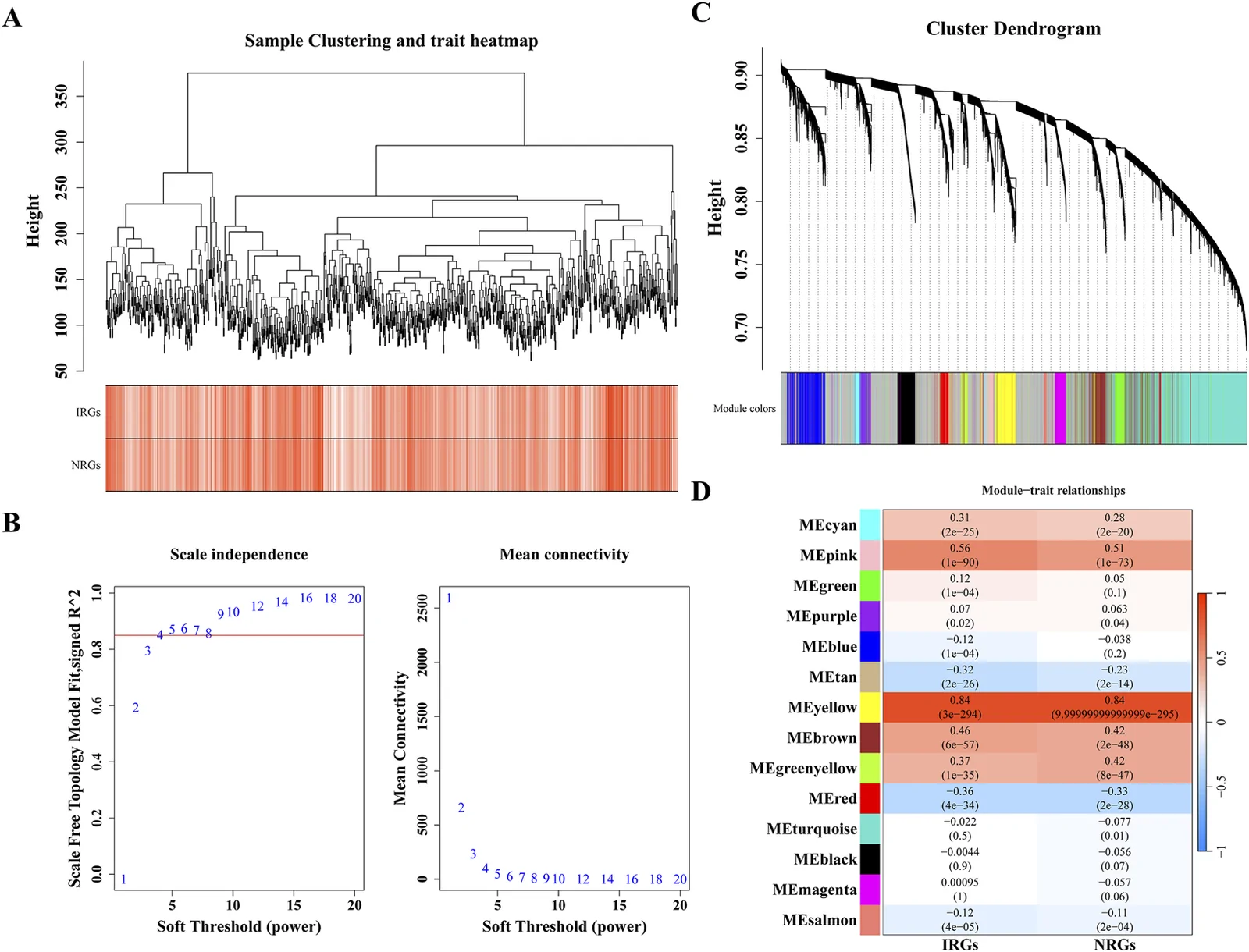
WGCNA module identification in TCGA-BRCA. **(A)** Sample clustering dendrogram with IRG and NRG trait heatmap; no outliers were excluded. **(B)** Scale-free topology fit index (R^2^) and mean connectivity plots used to select the optimal soft-thresholding power (β = 4, R^2^ = 0.85). **(C)** Gene dendrogram with 14 co-expression modules identified (gray module excluded). **(D)** Module–trait correlation heatmap (Pearson r; P-values in parentheses). The yellow module showed the strongest positive correlation with IRGs (r = 0.84, P = 3 × 10^−294^) and NRGs (r = 0.84, P = 9.9 × 10^−295^); the red module showed the strongest negative correlations (IRGs: r = −0.36, P = 4 × 10^−34^; NRGs: r = −0.33, P = 2 × 10^−28^).

To identify module genes strongly correlated with IRG and NRG scores, outlier samples were first excluded from the TCGA-BRCA BC dataset (Figure 3A). As evidenced by the sample clustering dendrogram, no samples exceeded the height threshold of 150 and were therefore retained for downstream analysis. The minimum soft threshold for constructing a scale-free network was determined to be 4, based on a scale-free fit index of R^2^ = 0.85, with network connectivity nearing zero (Figure 3B). WGCNA analysis identified 14 modules (excluding the gray module) (Figure 3C). The module sizes ranged from the smallest (MEsalmon) to the largest (yellow module), reflecting substantial heterogeneity in co-expression cluster sizes across the 14 identified modules. Among these, the yellow module, which contained 921 genes, exhibited the strongest positive correlation with both IRGs (r = 0.84, P = 3 × 10^−294^) and NRGs (r = 0.84, P = 9.9 × 10^−295^). The red module, comprising 792 genes, showed the strongest negative correlation with both IRGs (r = −0.36, P = 4 × 10^−34^) and NRGs (r = −0.33, P = 2 × 10^−28^) (Figure 3D). Integration of these two key modules yielded a total of 1,713 key module genes (Supplementary Table S6).

### 3.4 Determination and enrichment of 104 candidate genes

**FIGURE 4:**
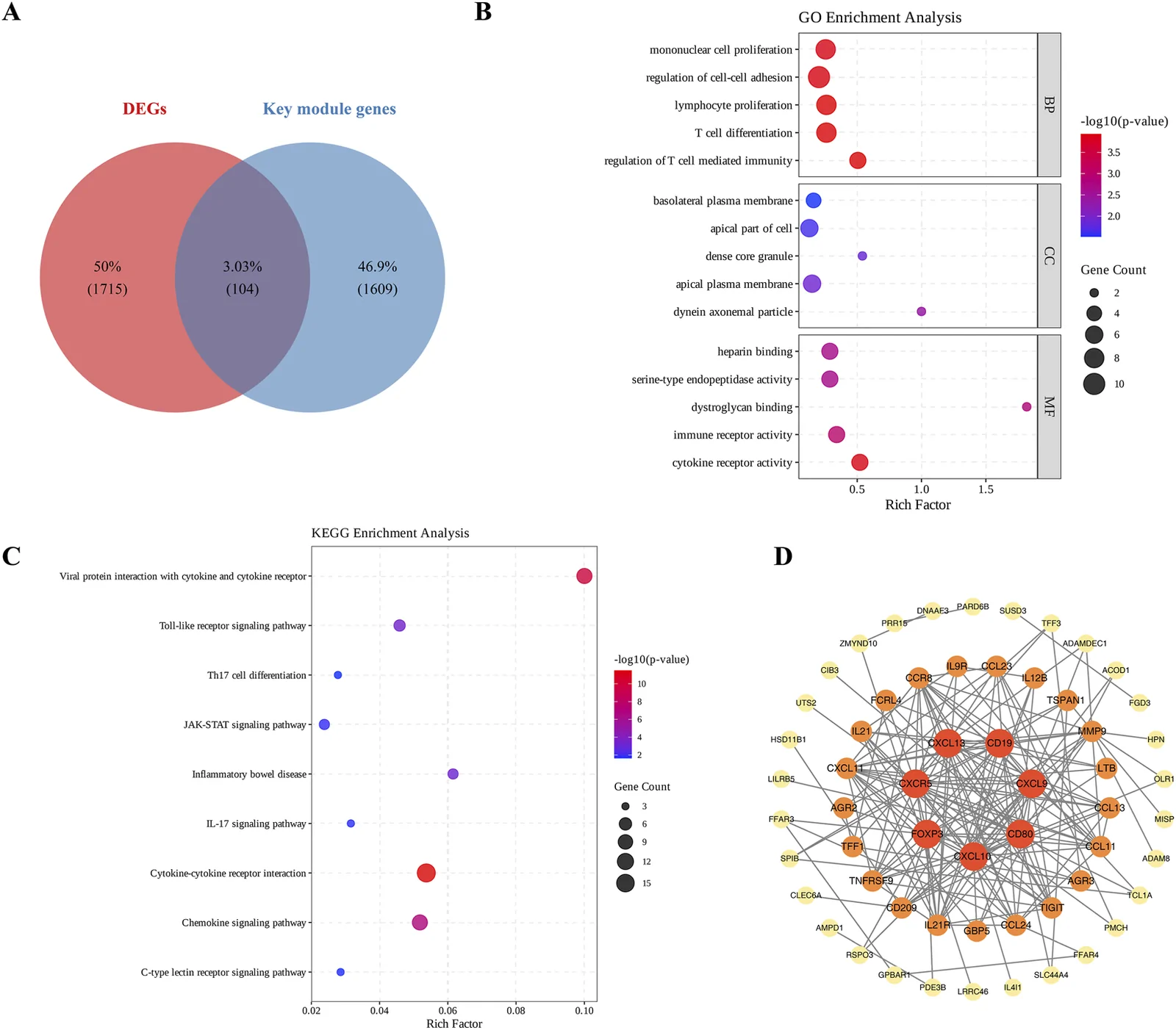
Identification and enrichment of 104 candidate genes. **(A)** Venn diagram showing overlap between 1,819 DEGs and 1,713 key module genes, yielding 104 candidate genes. **(B)** GO enrichment circular plot of 104 candidate genes (Fisher’s exact test, Benjamini–Hochberg correction, p < 0.05). **(C)** KEGG pathway enrichment circular plot; with Cytokine–cytokine receptor interaction ranked highest. **(D)** PPI network of 104 candidate genes; 46 isolated nodes excluded, leaving 58 hub nodes (e.g., CXCR5, CD19, CD80).

From the 1,713 key module genes and 1,819 differentially expressed genes, 104 candidate genes were identified (Figure 4A). A GO analysis of these 104 candidate genes identified 959 significantly enriched GO terms (p < 0.05), including 395 in biological processes (BPs), nine in cellular components (CCs), and 34 in molecular functions (MFs). The top five BP terms ranked by significance were mononuclear cell proliferation, regulation of cell-cell adhesion, lymphocyte proliferation, T cell differentiation, and regulation of T cell mediated immunity. In CCs, basolateral plasma membrane, basolateral plasma membrane, dense core granule, apical plasma membrane, and dynein axonemal particle were significantly enriched. In MFs, heparin binding, serine-type endopeptidase activity, dystroglycan binding, immune receptor activity, and cytokine receptor activity were significantly enriched (Figure 4B; Supplementary Table S7). Subsequently, all 46 KEGG pathways were enriched (p < 0.05). The top 10 significantly enriched KEGG pathways, ranked by significance from highest to lowest, wereViral protein interaction with cytokine and cytokine receptor, Toll-ike receptor signaling pathway, Th17 cell differentiation, JAK-STAT signaling pathway, Inflammatory bowel disease, Th17 cell differentiation, LL-17 signaling pathway,Cytokine-cytokine receptor interaction, Chemokine signaling pathway, C-type lectin receptor signaling pathway (Figure 4C; Supplementary Table S8). These pathways were primarily related to viral pathogenesis and immune regulation. The PPI network comprised 58 nodes and visually demonstrated a densely interconnected core, with hub nodes such as CXCR5, CD19, and CD80 showing the highest degree of connectivity among the retained genes (Figure 4D).

### 3.5 Building and validating a risk model

**FIGURE 5:**
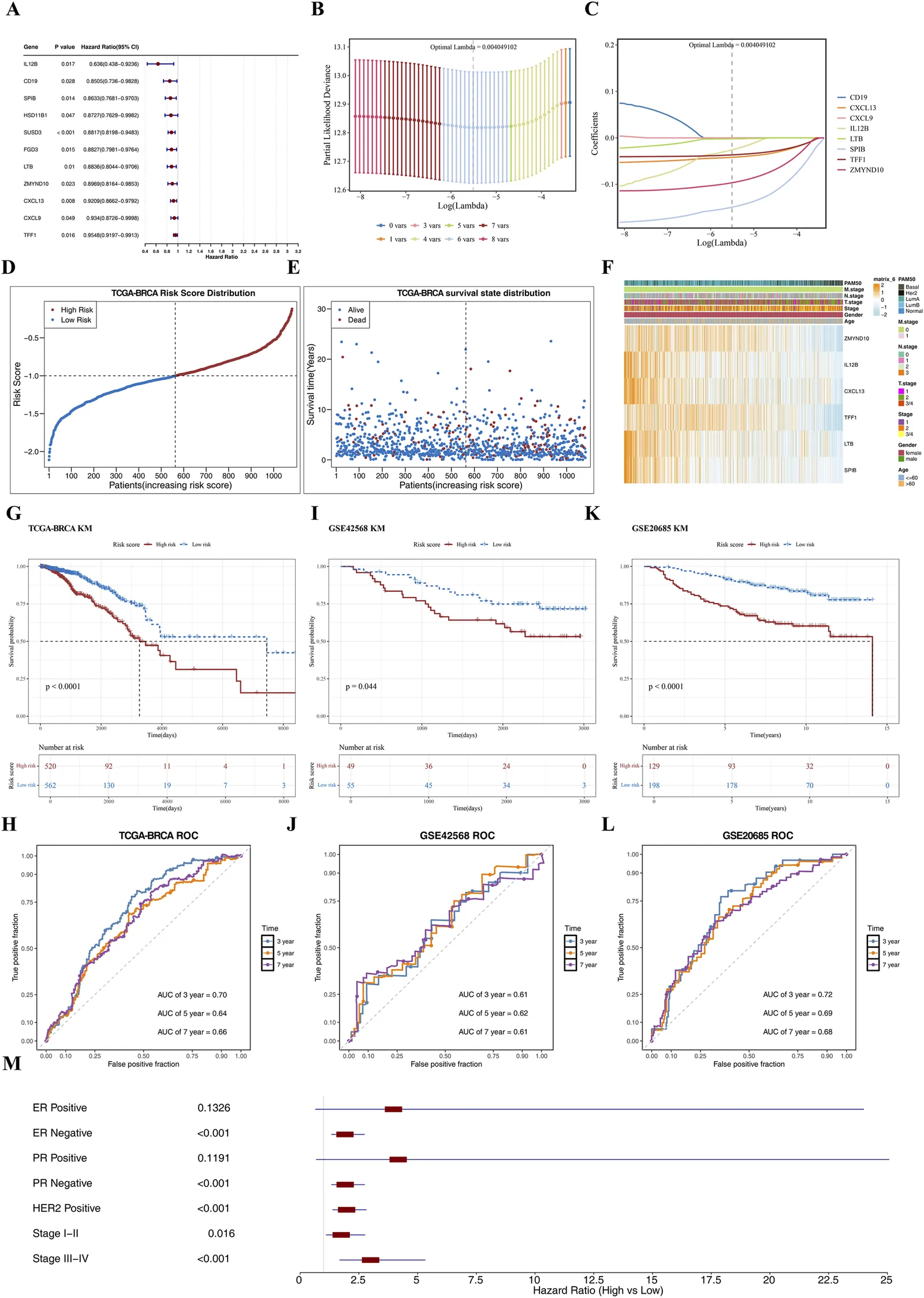
Determination of independent prognostic factors and construction of a risk model. **(A)** Univariate Cox regression forest plot of 11 candidate prognostic genes. Hazard ratios (95% CI) and unadjusted p-values are shown; genes with p < 0.05 were retained for further selection. **(B)** LASSO penalized Cox regression: 10-fold cross-validation partial likelihood deviance versus log(λ). Optimal λ = 0.004049 (dashed line). **(C)** LASSO coefficient paths versus log(λ). Eight genes (CD19, *CXCL13*, CXCL9, *IL12B*, *LTB*, *SPIB*, *TFF1*, *ZMYND10*) were retained at the optimal λ. **(D)** Risk score distribution for each patient in the training cohort (n = 1,082), ranked by increasing risk score. Red/blue indicate high- and low-risk groups based on the median cutoff. **(E)** Corresponding survival status (alive/dead) and survival time for each patient in the training cohort (n = 1,082). **(F)** Heatmap of expression levels of the six signature genes (*ZMYND10*, *IL12B*, *CXCL13*, *TFF1*, *LTB*, *SPIB*) across high- and low-risk patients in the training cohort. **(G)** K-M overall survival curves for high-risk (n = 520) and low-risk (n = 562) groups in the training cohort. p < 0.0001 by log-rank test. **(H)** Time-dependent ROC curves at 3, 5, and 7 years in the training cohort. AUC: 3-year = 0.70, 5-year = 0.64, 7-year = 0.68. **(I)** K-M overall survival curves for high-risk (n = 49) and low-risk (n = 55) groups in validation cohort 1. p = 0.044 by log-rank test. **(J)** Time-dependent ROC curves at 3, 5, and 7 years in the validation cohort (GSE42568). AUC: 3-year = 0.61, 5-year = 0.62, 7-year = 0.61. **(K)** K-M overall survival curves for high-risk (n = 129) and low-risk groups (n = 198) in validation cohort 2. p < 0.0001 by log-rank test. **(L)** Time-dependent ROC curves at 3, 5, and 7 years in validation cohort (GSE20685). AUC: 3-year = 0.72, 5-year = 0.69, 7-year = 0.68. **(M)** Subgroup forest plot showing the prognostic performance of the risk score across different clinical subgroups, including ER, PR, HER2 status, and tumor stage.

Regression analysis using a univariate Cox model was performed on the 58 key nodes identified from the PPI network. Further validation through the proportional risk test revealed genes strongly associated with BC prognosis (Figure 5A; Table 1). In subsequent LASSO regression analysis, with an optimal lambda of 0.004049102, six prognostic genes were selected from eight candidates: *ZMYND10*, *IL12B*, *CXCL13*, *TFF1*, *LTB*, and *SPIB* (Figures 5B,C). The risk score for BC samples in the training set were calculated using the formula: risk score = *ZMYND10* × (−0.096620187) + *IL12B* × (−0.026057922) + *CXCL13* × (−0.041857438) + *TFF1* × (−0.036619288) + *LTB* × (−0.002043212) + *SPIB* × (−0.149233102). Patients with BC were categorized into HRG (520 samples) and LRG (562 samples) based on the optimal cutoff value of −1.000475, determined by the surv_cutpoint function (maximally selected rank statistics) in the training cohort. The risk score distribution curve demonstrated a clear separation between the HRG and LRG at the optimal cutoff of −1.000475, with risk scores ranging from approximately −2.5 to 0.5 (Figure 5D). Survival status distribution showed that the majority of death events (red dots) were concentrated in the HRG, whereas patients in the LRG were predominantly alive (blue dots), confirming a positive correlation between risk score and mortality (Figure 5E). In the clinical feature association analysis, the expression levels of six prognostic genes were significantly correlated with multiple clinical variables (Figure 5F). K-M analysis demonstrated significantly worse overall survival in the HRG compared with the LRG in the training cohort (P < 0.0001; Figure 5G). The ROC curves for 3, 5, and 7-year survival rates demonstrated AUC values of 0.7, 0.64, and 0.66 (Figure 5H). It demonstrated moderate predictive accuracy of therisk model. Bootstrap internal validation (n = 1,000) yielded an optimism-corrected C-index of 0.621 (95% CI: 0.582–0.683), compared with the apparent C-index of 0.627, confirming minimal overfitting and satisfactory model robustness (Supplementary Figure S3B).

**TABLE 1:** PH assumption testing for prognosis-related genes identified through univariate Cox regression analysis.

Gene	chisq	df	p-value
*ZMYND10*	0.489398598830252	1	0.484195698425821
*IL12B*	3.41950429004041	1	0.0644303574474851
*CXCL9*	0.517449974361823	1	0.471931481980269
*CXCL13*	3.18682918462583	1	0.0742338711147542
*TFF1*	0.435380362874443	1	0.509360626857443
*CD19*	0.0625652598810622	1	0.802486440953522
*LTB*	0.16519213690028	1	0.684420542277235
*SPIB*	0.103569077843986	1	0.747587925475872

The risk model was further validated using the GSE42568 and GSE20685 datasets. In the GSE42568 dataset, patients were categorized into HRG (49 samples) and LRG (55 samples) based on an optimal risk score cutoff of −1.558635 (Supplementary Table S9). The analysis of the GSE42568 dataset, including survival time and risk score variations between HRG and LRG patients, yielded results consistent with those from the TCGA-BRCA dataset (Supplementary Figure S4). Validation in the GSE42568 dataset similarly showed significantly worse survival in the HRG than in the LRG (P = 0.044; Figure 5I). Additionally, the ROC curves for 3, 5, and 7-year survival rates demonstrated AUC values of 0.61, 0.62, and 0.61 (Figure 5J). Bootstrap internal validation (n = 1,000) yielded an optimism-corrected C-index of 0.621 (95% CI: 0.489-0.711), compared with the apparent C-index of 0.610 (Supplementary Figure S3C). At the same time, in dataset GSE20685, according to the optimal risk score threshold of −2.56009, BC patients were divided into 198 cases in the HRG and 129 cases in the low-risk gene group LRG (Supplementary Table S10). The analysis results showed that the survival time and risk changes were consistent with those in the training set (Supplementary Figure S5). In the external validation cohort of GSE20685, the survival rate of HRG patients was significantly lower than that of LRG patients (P < 0.0001; Figure 5K), which preliminarily confirmed the prognostic stratification ability of the risk model; meanwhile, the time-dependent ROC analysis showed that the corresponding AUC of the model at the third, fifth, and seventh years were 0.72, 0.69, and 0.68 (Figure 5L), suggesting that it had moderate predictive efficacy in the validation set. To further evaluate the model’s stability, we calculated the C-index based on Bootstrap internal validation (repeated sampling 1,000 times). The results showed that the corrected C-index after optimism adjustment was 0.669 (95% confidence interval: 0.613-0.724), while the apparent C-index was 0.667 (Supplementary Figure S3D); the corrected estimated value was close to the apparent value, and the confidence interval did not contain 0.5, indicating that the model’s discrimination was robust and reliable. Based on the above results, this risk model was supported to have good predictive performance in independent validation.

To further evaluate the robustness of the risk model across clinically relevant subgroups, stratified Cox regression analyses were performed in the GSE42568 dataset by molecular subtype (ER, PR, and HER2 status) and clinical stage. The risk score demonstrated significant prognostic value in ER-negative (HR = 1.91, P < 0.001), PR-negative (HR = 1.92, P < 0.001), HER2-positive (HR = 1.98, P < 0.001), Stage I–II (HR = 1.75, P = 0.016), and Stage III–IV (HR = 3.00, P < 0.001) subgroups, with high-risk patients consistently showing worse outcomes. In ER-positive (HR = 3.97, P = 0.133) and PR-positive (HR = 4.17, P = 0.119) subgroups, the risk ratios were elevated but did not reach statistical significance, likely attributable to the limited sample size in these subgroups (Figure 5M).

### 3.6 Construction of the prognostic model and model stability testing

**FIGURE 6:**
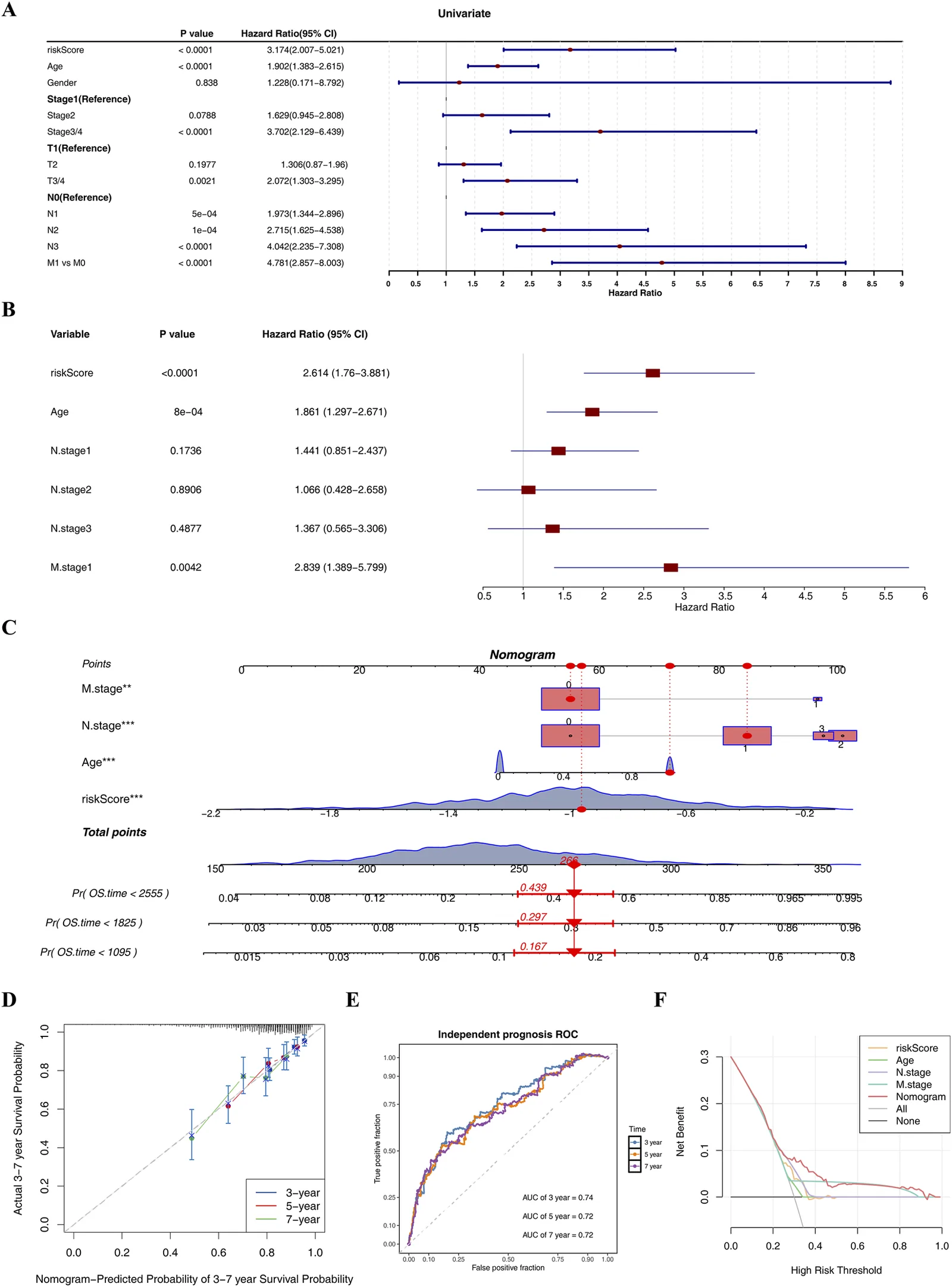
Nomogram development and validation. **(A)** Univariate Cox regression forest plot for clinical variables and risk score. Significant factors: riskScore (HR = 3.174, 95% CI: 2.007–5.021, P < 0.0001), Age (HR = 1.902, 95% CI: 1.383–2.615, P < 0.0001), Stage3/4 (HR = 3.702, 95% CI: 2.129–2.439, P < 0.0001), T3/4 (HR = 1.306, 95% CI: 1.303–3.295, P = 0.0021), N1 (HR = 1.973, 95% CI: 1.334–2.896, P = 5 × 10^−4^), N3 (HR = 4.042, 95% CI: 2.235–7.308, P < 0.0001), M1 vs. M0 (HR = 4.781, 95% CI: 2.857–8.003, P < 0.0001). Stage and T.stage violated the PH assumption (cox.zph: P = 0.001 and P = 0.007, respectively) and were incorporated as stratification variables. **(B)** Multivariable stratified Cox regression forest plot; independent prognostic factors: riskScore (HR = 2.614, 95% CI:1.76–3.881, P < 0.001), Age (HR = 1.861, 95% CI: 1.297–2.671, P = 8.0 × 10^−4^), M. stage1 (HR = 2.839, 95% CI: 1.389–5.799, P = 0.0042). **(C)** Nomogram integrating riskScore, Age, N.stage, and M.stage for individualized 3-, 5-, and 7-year OS prediction. **(D)** Calibration curves for 3-, 5-, and 7-year survival; error bars represent 95% CI. **(E)** Time-dependent ROC curves; AUC: 3-year = 0.74, 5-year = 0.72, 7-year = 0.72. **(F)** Decision curve analysis comparing nomogram versus individual variables and reference strategies.

Univariate Cox regression identified risk score (HR = 3.174, 95% CI: 2.007–5.021, P < 0.0001), Age (HR = 1.902, 95% CI: 1.383–2.615, P < 0.0001), Stage 3/4 (HR = 3.702, 95% CI: 2.129–6.439, P < 0.0001), T3/4 (HR = 2.072, 95% CI: 1.303–3.295, P = 0.0021), N1 (HR = 1.973, 95% CI: 1.344–2.896, P = 5.0 × 10^−4^), N3 (HR = 4.042, 95% CI: 2.235–7.308, P < 0.0001), and M1 (HR = 4.781, 95% CI: 2.857–8.003, P < 0.0001) as significant prognostic factors. cox.zph testing confirmed that riskScore (χ^2^ = 0.199, P = 0.656) and Age (χ^2^ = 0.047, P = 0.828) satisfied the proportional hazards assumption, while Stage (χ^2^ = 13.602, P = 0.001) and T.stage (χ^2^ = 10.026, P = 0.007) violated it; N.stage (χ^2^ = 6.799, P = 0.079) and M.stage (χ^2^ = 2.095, P = 0.148) also satisfied the assumption (Figure 6A; Table 2). Accordingly, a stratified Cox model was constructed incorporating Stage and T.stage as stratification variables. Multivariable analysis identified riskScore (HR = 2.614, 95% CI: 1.760–3.881, P < 0.001), Age (HR = 1.861, 95% CI: 1.297–2.671, P = 8.0 × 10^−4^), and M.stage (HR = 2.839, 95% CI: 1.389–5.799, P = 0.0042) as independent prognostic factors for BC (Figure 6B). A nomogram was then developed incorporating these factors. According to the nomogram model, higher total scores for each patient correspond to an increased mortality rate and, conversely, a lower survival probability (Figure 6C). The calibration curve for the nomogram closely aligned with the reference line, with calibration intercepts of −0.134, −1.007, and −1.491, and calibration slopes of 0.133, 0.970, and 1.398 at 3, 5, and 7 years, respectively, demonstrating satisfactory agreement between predicted and observed survival probabilities (Figure 6D; Table 3). Additionally, time-dependent ROC analysis demonstrated moderate predictive performance of the nomogram model, with AUC values of 0.74, 0.72, and 0.72 at 3, 5, and 7 years, respectively (Figure 6E). Furthermore, the decision curve analysis revealed that the nomogram provided additional clinical benefit, with a net benefit greater than zero (Figure 6F).

**TABLE 2:** PH assumption testing for independent prognostic factors identified through univariate Cox regression analysis.

​	chisq	df	p
riskScore	0.19866668445802	1	0.655799219113232
Age	0.0472310157441512	1	0.827953533402815
Stage	13.601943443087	2	0.00111269339420582
T.stage	10.0262732676359	2	0.00665001190681172
N.stage	6.76990949745186	3	0.0796045927814619
M.stage	2.09463121162874	1	0.147817375726045

**TABLE 3:** Multivariate Cox regression analysis of the risk score and clinicopathological variables.

​	coef	exp (coef)	se (coef)	z	Pr (>|z|)
Risk score	0.827360236627102	2.28727290474962	0.194798463493963	4.24726264153897	2.16398227964911e-05
Age	0.657983133816338	1.93089404965115	0.177780368940457	3.70110118309357	0.00021466584868698
N. stageN1	0.602285546423013	1.82628810024629	0.207723384921542	2.89945952233783	0.00373806606731112
O. stageN2	1.02177099047817	2.77811041698795	0.281838119529591	3.62538251455688	0.000288534049821511
N.stageN3	1.13901686544176	3.12369584175019	0.350570739743131	3.24903574746807	0.00115796925140333
M. stageM1	0.885989864026216	2.42538400401989	0.316671458694737	2.79782038987065	0.00514487133585561

### 3.7 Clinical significance of the risk model

**FIGURE 7:**
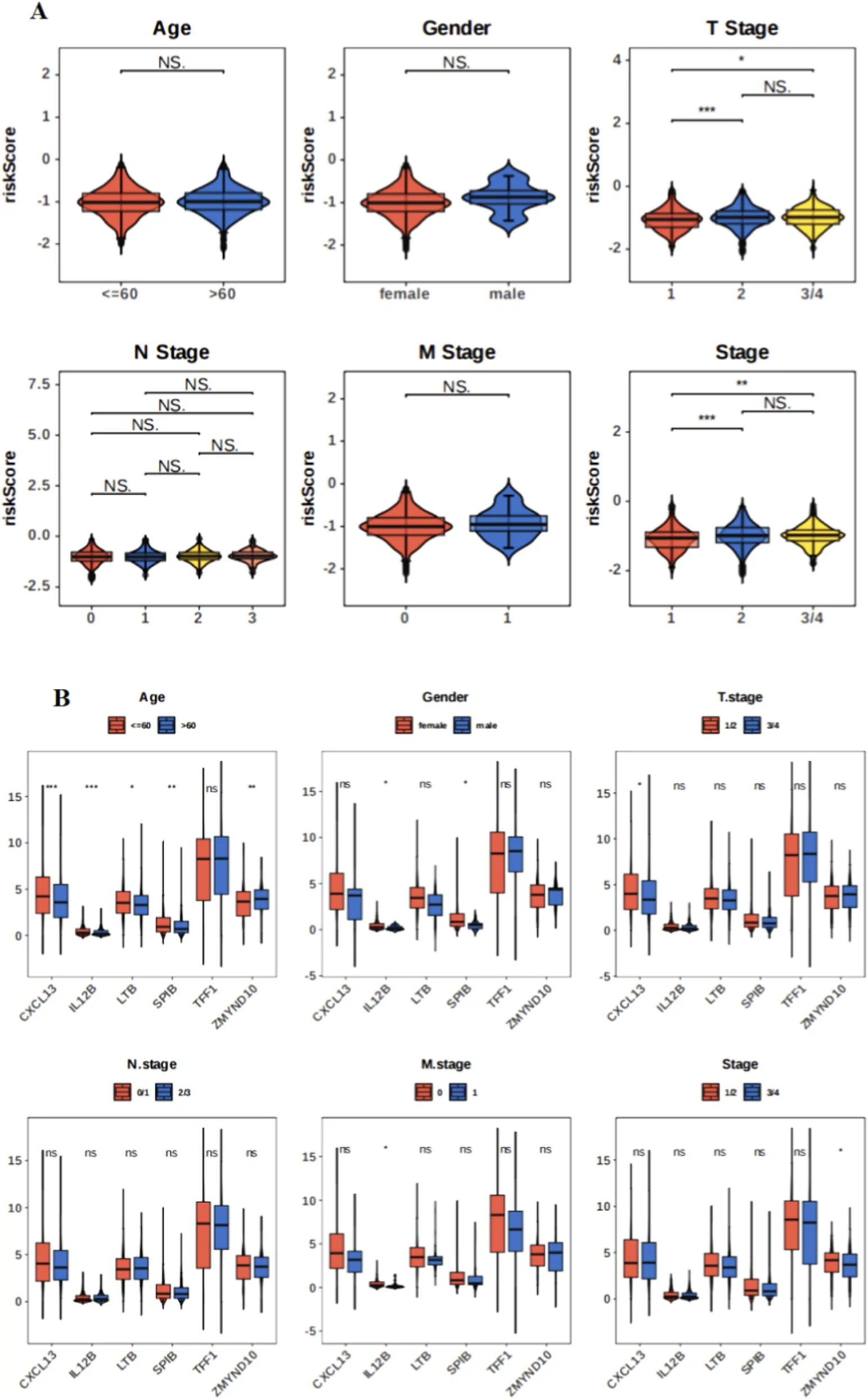
Risk score and prognostic gene expression across clinical subgroups. **(A)** Violin plots of risk score distribution across Age, Gender, T stage, N stage, M stage, and overall Stage subgroups. Comparisons by Wilcoxon rank-sum test or Kruskal–Wallis test with Dunn’s post-hoc correction. * represents p < 0.05, **represents p < 0.01, ***represents p < 0.001; NS, not significant. **(B)** Box plots of mRNA expression of six prognostic genes stratified by the same clinical variables. Comparisons by Wilcoxon rank-sum test. Significant associations: *IL12B* and *SPIB* by Gender; *CXCL13*, *IL12B*, *LTB*, *SPIB*, *ZMYND10* by Age; *CXCL13* by T stage; *IL12B* by M stage; *ZMYND10* by Stage (all P < 0.05).

Risk scores differed significantly across T stages (T1 vs. T3/4: P < 0.001; T2 vs. T3/4: NS) and Stage groups (Stage 1 vs. Stage 3/4: P < 0.001; Stage 2 vs. Stage 3/4: P < 0.01), while no significant differences were observed across Age, Gender, N stage, or M stage subgroups (all P > 0.05) (Figure 7A). Among the six prognostic genes, *IL12B* and *SPIB* showed significantly different expression between females and males (P < 0.05), while *CXCL13*, *IL12B*, *LTB*, *SPIB*, and *ZMYND10* expression varied significantly across age groups (P < 0.05). *CXCL13* expression differed significantly between T1/2 and T3/4 (P < 0.05), *IL12B* between M0 and M1 (P < 0.05), and *ZMYND10* across Stage groups (P < 0.05) (Figure 7B).

### 3.8 Immune microenvironment analysis

**FIGURE 8:**
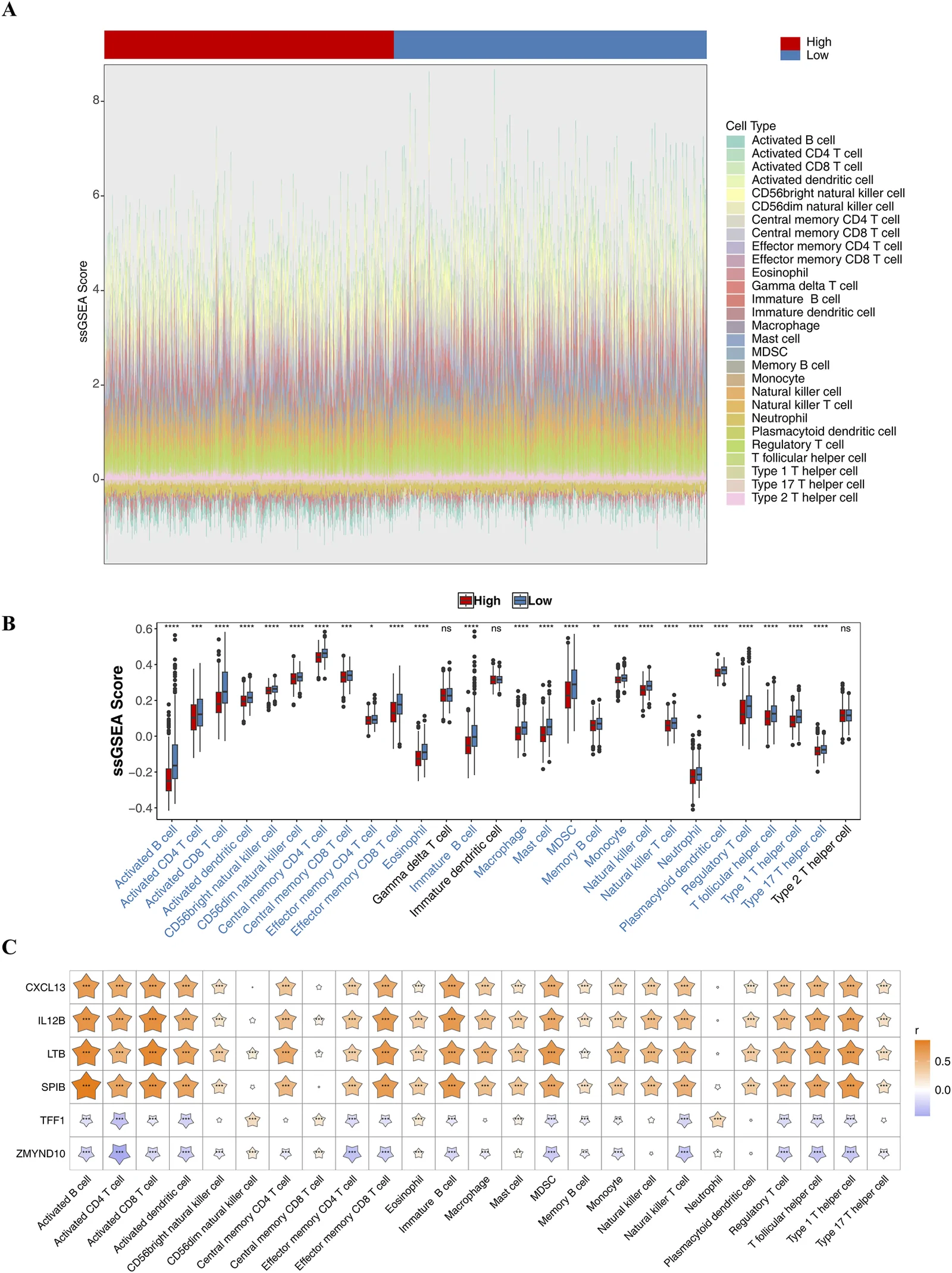
Immune microenvironment analysis between HRG and LRG. **(A)** Stacked bar chart of ssGSEA infiltration scores for 28 immune cell types across all samples, ordered by risk group (HRG, red; LRG, blue). **(B)** Box plots comparing ssGSEA scores of 28 immune cell types between HRG and LRG (Wilcoxon rank-sum test, Benjamini–Hochberg correction). Significant differences observed in 25 of 28 cell types (p.adj <0.05); gamma delta T cells, immature dendritic cells, and type 2 T helper cells were non-significant. **(C)** Spearman correlation bubble plot between six prognostic genes and 28 immune cell types. Bubble size represents |r|; color indicates direction. * represents p < 0.05, **represents p < 0.01, ***represents p < 0.001.

A statistically significant difference in the infiltration abundance of 28 immune cell types was observed between HRG and LRG. Natural killer cells and T cells showed relatively high infiltration abundance in both groups (Figure 8A). Apart from gamma delta T cells, immature dendritic cells, and type 2 T helper cells, significant differences were observed in the infiltration rates of the remaining 25 immune cell types (Figure 8B; Supplementary Table S11). The CIBERSORT algorithm independently verified the differential infiltration of monocytes and eosinophils between the two groups, supporting the robustness of the ssGSEA results. Correlation analysis between the six prognostic hub genes and 25 immune cell types revealed distinct and consistent patterns. *SPIB* exhibited the strongest positive correlation with Activated B cells (r = 0.864, 95% CI: 0.848–0.878, FDR-adjusted P < 0.001), followed by *LTB* (r = 0.792, 95% CI: 0.768–0.813) and *IL12B* (r = 0.720, 95% CI: 0.690–0.747), while *CXCL13* also showed a strong positive correlation (r = 0.674, 95% CI: 0.641–0.706). These four genes were broadly positively correlated with the majority of immune cell types examined, including Activated CD8 T cells, Effector memory CD8 T cells, Immature B cells, MDSC, and Type 1 T helper cells (all FDR-adjusted P < 0.001). In contrast, *TFF1* displayed predominantly negative correlations with most immune cell types, but showed a modest yet significant positive correlation with Eosinophils (r = 0.171, 95% CI: 0.112–0.228, FDR-adjusted P < 0.001) and Neutrophils (r = 0.274, 95% CI: 0.218–0.328, FDR-adjusted P < 0.001). *ZMYND10* showed the strongest negative correlation with Activated CD4 T cells (r = −0.459, 95% CI: −0.505 to −0.410, FDR-adjusted P < 0.001) and Activated dendritic cells (r = −0.270, 95% CI: −0.325 to −0.214, FDR-adjusted P < 0.001), with consistently negative associations across most immune subsets (Figure 8C; Supplementary Table S12). Furthermore, Spearman correlation analysis revealed that ELANE was significantly negatively correlated with the risk score (r = −0.162, 95% CI: −0.222 to −0.098, p.adjust <0.001), providing a direct link between the risk model and neutrophil-mediated NET biology (Supplementary Table S13).

### 3.9 Tissue expression differences of signaling pathways and prognostic genes

**FIGURE 9:**
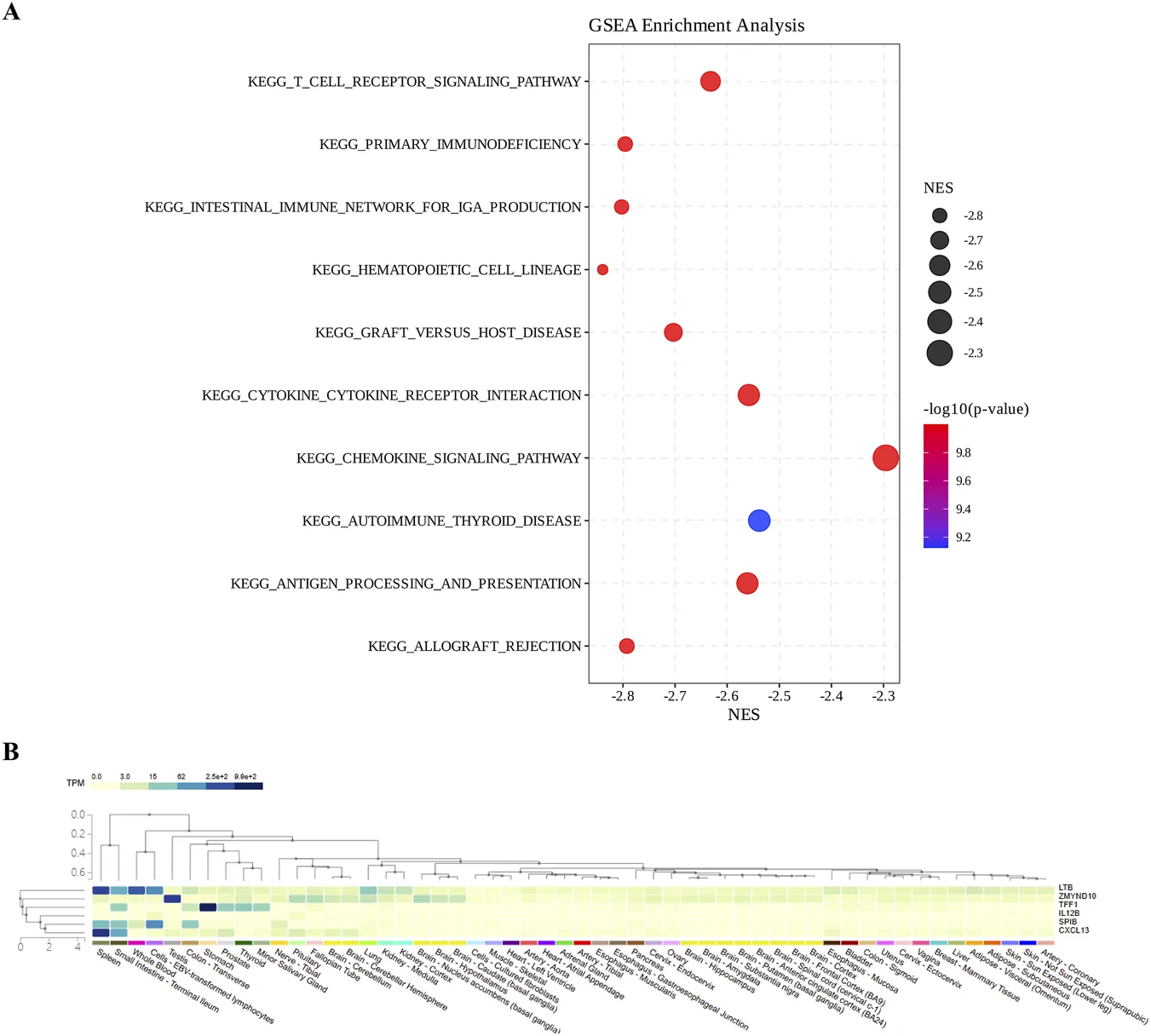
Tissue expression differences of signaling pathways and prognostic genes. **(A)** GSEA enrichment plots for the top five enriched KEGG pathways comparing HRG versus LRG. All five pathways showed negative NES, indicating downregulation in HRG. **(B)** Heatmap of tissue-specific expression (Human Protein Atlas) of six prognostic genes across human tissues.

GSEA identified 26 enriched pathways, including hematopoietic cell lineage, intestinal IgA production immune network, and allograft rejection (Figure 9A). The top ten enriched pathways shown include T cell receptor signaling pathway, primary immunodeficiency, intestinal immune network for iga production, alhematopoietic cell lineage, etc., all demonstrating negative enrichment scores (NES <0), indicating downregulation of these immune-related pathways in the high-risk group. The analysis of prognostic gene expression showed that *LTB* and *SPIB* were predominantly expressed in the spleen, small intestine-terminal ileum, and EBV-transformed lymphocytes; *ZMYND10* was highly expressed in the testis; *TFF1* was notably expressed in the stomach; and *CXCL13* was primarily expressed in the spleen (Figure 9B). The significant expression of *LTB*, *SPIB*, and *CXCL13* in the spleen highlights their potential role in the spleen’s physiological functions, positioning it as a key immune organ.

### 3.10 lncRNA-miRNA-mRNA network construction, drug sensitivity and immunotherapy response analysis

**FIGURE 10:**
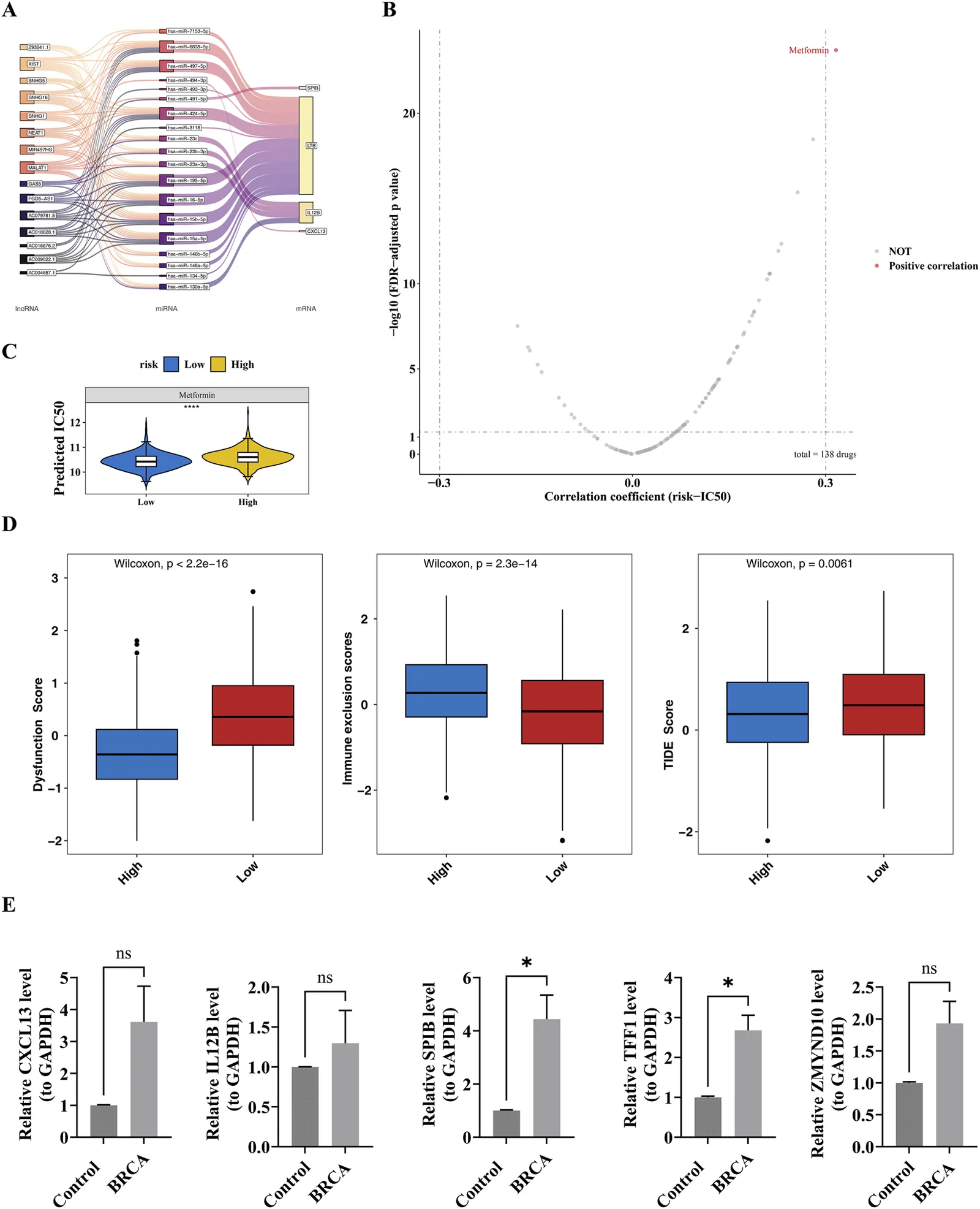
lncRNA-miRNA-mRNA network construction, drug sensitivity analysis, and *in vitro* experimental results. **(A)** Sankey diagram of the lncRNA-miRNA-mRNA regulatory network comprising four prognostic genes (SPIB, LTB, IL12B, CXCL13), 19 miRNAs, and 15 lncRNAs. **(B)** Volcano plot showing the correlation between predicted IC50 values of 138 chemotherapy drugs and the risk score. Dashed lines indicate thresholds of |r| = 0.3 and FDR-adjusted P = 0.05. **(C)** Violin plot comparing predicted Metformin IC_50_ between high- and low-risk groups (****P < 0.0001). **(D)** Comparison of TIDE scores, immune exclusion scores, and dysfunction scores between high- and low-risk groups. **(E)** Bar plots showing relative mRNA expression levels of prognostic genes normalized to GAPDH in BRCA tumor tissues versus normal controls (paired t-test; ns, not significant; *P < 0.05).

Among the six prognostic genes, only four were predicted to interact with 19 miRNAs and 15 lncRNAs. Specifically, *SPIB* interacted with one miRNA (hsa-miR-491-5p), *LTB* was associated with 13 miRNAs (such as hsa-miR-7153-5p), *IL12B* interacted with four miRNAs (such as hsa-miR-23c), and *CXCL13* showed a strong interaction with one miRNA (hsa-miR-494-3p). This interaction network led to the construction of an lncRNA-miRNA-mRNA regulatory network consisting of four prognostic genes (*SPIB*, *LTB*, *IL12B*, *CXCL13*), 19 miRNAs, and 15 lncRNAs (Figure 10A). From 138 common chemotherapy drugs evaluated, only Metformin showed a significant positive correlation with the risk score (FDR-adjusted P < 0.05, |r| > 0.3; r = 0.316, 95% CI: 0.262–0.369, FDR-adjusted P < 0.001; Figure 10B; Supplementary Table S14). Comparison of predicted IC50 values between high- and low-risk groups revealed that Metformin exhibited a significantly higher median IC50 in the high-risk group than in the low-risk group (Δ = 0.181, Wilcoxon P < 0.001, FDR-adjusted P < 0.001; Figure 10C; Supplementary Table S15), suggesting that high-risk patients may require higher drug concentrations to achieve equivalent pharmacological effects. However, as these estimates are derived from cell-line-based computational predictions, the findings should be interpreted with caution and require validation in independent pharmacogenomic databases and clinically relevant cohorts before any therapeutic implications can be inferred.

TIDE analysis revealed that TIDE scores, immune exclusion scores, and dysfunction scores were all significantly different between the high- and low-risk groups (all P < 0.05, Figure 10D). This result suggests that the risk model is significantly associated with multiple dimensions of immunotherapy resistance, including immune cell exclusion and T cell dysfunction, further supporting that patients in the high-risk group may derive less benefit from immune checkpoint inhibitor therapy.

### 3.11 Analysis of *in vitro* experimental results

RT-qPCR analysis confirmed the differences in the relative expression levels of mRNA between breast cancer samples and control samples. Specifically, five genes showed high expression in the disease group, but only *TFF1* (*P < 0.05) and *SPIB* (*P < 0.05) were significantly expressed in the disease group, while the expression of the remaining genes might be related to the sample size (Figure 10E). *LTB* was not included in the experimental validation, as the available tissue volume from each patient was inherently constrained by the clinical sampling protocol conducted under the existing ethical approval framework (K-ky2023041); additional tissue collection solely for research validation purposes was not ethically permissible within the scope of this study. Overall, all five experimentally tested genes demonstrated expression trends concordant with the *in silico* predictions, although statistical significance was reached for only two of them.

## 4 Discussion

BC involves malignant cells and significant alterations in the surrounding stroma and tumor microenvironment. The tumor microenvironment is recognized as a critical factor in the growth and progression of solid tumors, including BC (Sabit et al., 2025). Research has shown that inflammatory markers within the tumor microenvironment can serve as predictors for BC progression and prognosis. Inflammation acts as a key determinant of BC outcomes. Although the connection between BC and inflammation is well-documented, the specific roles of neutrophils and other inflammatory mediators in the tumor microenvironment, as well as their impact on BC progression, remain inadequately understood. This study applied bioinformatics and transcriptome sequencing to explore the mechanisms of NRGs and IRGs in BC, examining the biological pathways and regulatory processes of these genes and providing new insights into BC prognosis and treatment. Six prognostic genes associated with BC were identified: *ZMYND10*, *IL12B*, *CXCL13*, *TFF1*, *LTB*, and *SPIB*. Based on these genes, a risk score model was constructed, providing important insights into the role of NETs and inflammation in BC. This model demonstrates moderate predictive performance in clinical applications, though its Area Under the Curve (AUC) value is currently slightly lower than that of established commercial tools like Oncotype DX (Boehm et al., 2025). However, our model possesses a unique merit: it specifically incorporates genes relevant to NETosis and inflammation, thereby expanding the scope of prognostic markers recognized in BC. Oncotype DX primarily assesses recurrence risk based on a panel of 21 genes (Whitney et al., 2018). In contrast, our model incorporates six genes that exhibit strong associations with NETosis and inflammatory pathways, offering a distinct perspective for investigating BC prognosis.

Among the six identified prognostic genes, *ZMYND10* has attracted significant attention due to its well-established tumor-suppressing properties. The *ZMYND10* gene encodes a protein with a MYND-type zinc finger domain, which may be involved in the assembly of motor proteins. Mutations in this gene are linked to primary ciliary dyskinesia. Previous studies have established *ZMYND10* as a tumor suppressor gene, capable of arresting the cell cycle, inducing apoptosis, and inhibiting angiogenesis and cell proliferation across various tumor types. Yan Wang et al. demonstrated that *ZMYND10* inhibits tumor growth in BC through the miR145-5p/NEDD9 pathway (Zhang et al., 2012). Within this study, a low expression level of the *ZMYND10* gene was associated with an adverse outcome in BC. Evidence suggests that the protein encoded by the *ZMYND10* gene functions as a tumor suppressor, inhibiting tumor cell proliferation through the regulation of the cell cycle and the promotion of apoptosis (Wang et al., 2019). This study further supports the hypothesis that *ZMYND10* may serve as a promising therapeutic target for BC.

Another important prognostic gene, *IL12B*, plays a critical and distinct role in BC. *IL12B* encodes a subunit of interleukin-12 (IL-12), a cytokine that influences T cells and natural killer (NK) cells, facilitating a range of biological functions. IL-12 bridges innate and adaptive immunity by activating both systems. It suppresses tumor growth by promoting NK and T cell expansion and cytotoxicity, enhancing IFN-γ production, boosting antibody-dependent cellular cytotoxicity (ADCC), and exerting anti-angiogenic effects. The therapeutic potential of IL-12 is still under investigation. Previous studies by Huanhuan Hu (Dong et al., 2012) and Xiao Zhao (Cheng et al., 2015) have highlighted the predictive value of *IL12B* in their models. Previous studies have shown that high expression of *IL12B* is associated with favorable prognosis in specific inflammation-associated cancers (Liu et al., 2011), which aligns with our findings. However, the expression of *IL12B* failed to reach statistical significance in the RT-qPCR validation (p = 0.4886). This may be attributed to the fact that *IL12B* is predominantly expressed in infiltrating immune cells, including macrophages and dendritic cells, rather than in tumor cells themselves (Ullrich et al., 2020). Consequently, its transcript level in total tissue RNA is highly susceptible to interindividual heterogeneity in immune infiltration. The limited sample size (n = 5 pairs) further reduced the statistical power to detect subtle expression alterations in the presence of such biological variation. Future validation in larger cohorts or the application of single-cell approaches may more accurately delineate the immune cell-dependent expression dynamics of *IL12B* within the tumor microenvironment.

*CXCL13*, another key prognostic gene, presents a complex and intriguing role in BC. Also known as B lymphocyte chemokine, *CXCL13* facilitates the migration and accumulation of B lymphocytes by binding to the CXCR5 receptor. It is the most highly expressed chemokine in BC tissue and may contribute to tumorigenesis or metastasis by regulating B cell behavior. Evidence highlights the critical role of B cells within the tumor microenvironment in cancer (Lin et al., 2024). Furthermore, the chemokine *CXCL13* modulates the tumor immune microenvironment by influencing B cell activities. Elevated *CXCL13* expression is linked to younger patients, histological grades 2/3, lymph node positivity, and estrogen receptor (ER) negativity (Chen et al., 2015), indicating a potential connection to poor prognosis. Absence of *CXCL13* enhances the efficacy of conventional chemotherapy agents such as cyclophosphamide and anti-PD-1 immunotherapy (Ren et al., 2022). J-J Ma demonstrated that an anti-*CXCL13* antibody induced apoptosis in MDA-MB-231 BC cells (Ma et al., 2018), while Licheng Xu’s use of a *CXCL13* inhibitor in a BC mouse model effectively suppressed tumor growth, suggesting a novel therapeutic approach for BC (Xu et al., 2018). However, conflicting findings also exist (Yau et al., 2010; Schmidt et al., 2018), as explained by Qizhi Ma’s study (Ma et al., 2021), which revealed that *CXCL13* directly influences cancer cells expressing CXCR5, promoting their proliferation, migration, and invasion. In the absence of endogenous CXCR5 expression on tumor cells, *CXCL13* secretion within the tumor microenvironment can attract CXCR5+ immune cells, initiating antigen-specific anti-tumor immunity. Given the complexity of the *CXCL13*/CXCR5 axis in the tumor microenvironment, careful evaluation of CXCR5 expression on tumor cells in clinical settings is essential. As a chemokine, *CXCL13* recruits immune cells into the Tumor Microenvironment (TME). In this specific model, high *CXCL13* expression correlates with favorable prognosis. This beneficial effect is likely attributable to the tumor-suppressing and metastasis-limiting actions of the recruited immune cells, underscoring *CXCL13*’s distinct role in immune regulation.

Trefoil factor 1 (*TFF1*), another prognostic gene, stands out due to its controversial role in BC. *TFF1*, a peptide predominantly expressed in the gastrointestinal tract, was first identified in BC cells (MCF-7) (Masiakowski et al., 1982), and its influence on BC remains debated. High expression of the *TFF1* gene was associated with favorable prognosis in this study. However, research by Spadazzi et al. reported that elevated *TFF1* expression correlates with increased bone metastasis risk in HR-positive BC (Spadazzi et al., 2021). This discrepancy may stem from heterogeneity in *TFF1*’s expression levels and functional roles across distinct molecular subtypes of BC. Therefore, future studies are warranted to further elucidate subtype-specific mechanisms of *TFF1* and their clinical implications. Conversely, Jie Yi’s research found that *TFF1* overexpression significantly inhibited BC cell proliferation, migration, and invasion *in vitro*, while *TFF1* knockout enhanced migration abilities (Yi et al., 2020). The predictive value of *TFF1* has been observed in multiple established prediction models (Chen X. et al., 2024; Cheng et al., 2023; Feng et al., 2023).

Lymphotoxin beta (*LTB*), another gene identified as a prognostic marker, plays a multifaceted and complex role in BC. As a type II membrane protein in the TNF family, *LTB* is involved in inducing the inflammatory response and is essential for the normal development of lymphoid tissue. Research by Martinet et al. (2013) highlighted *LTB*’s critical role in the formation of tumor high endothelial venules (HEVs). HEVs facilitate the infiltration of lymphocytes into tumors, promoting tumor destruction, and the loss of HEVs is a key step in BC progression. Therefore, high *LTB* expression is associated with a favorable prognosis in BC. This observation was also confirmed by Marchetti et al. (2021). Additionally, Payton De La Cruz found that *LTB*-induced NF-κB signaling is linked to tumor regression in TNBC mouse models (De et al., 2024). On the other hand, *LTB* stimulates osteoblasts via NF-κB2 signaling, leading to the secretion of CCL2/5, enhancing tumor cell-osteoblast adhesion, accelerating osteoclastogenesis, and promoting bone metastasis progression (Wang et al., 2024). *LTB* gene expression was elevated in this study and correlated with favorable prognosis. The protein it encodes participates in inflammatory responses and lymphoid tissue development. Furthermore, research indicates that *LTB* influences the tumor microenvironment by inducing inflammation and lymphocyte infiltration, ultimately promoting tumor progression (Subramanian et al., 2017).

Finally, among the prognostic genes associated with NETosis and inflammation in BC, the Spi-B transcription factor (*SPIB*) also holds great potential for understanding BC biology. *SPIB* is crucial in regulating gene expression, significantly impacting biological processes, particularly the immune system and cancer development. *SPIB* regulates the development and function of immune cells. Research demonstrates its critical role in immune cell differentiation and immune responses. Significantly, within the tumor microenvironment, *SPIB* exerts anti-tumor effects by promoting the activation and functionality of immune cells (Huang et al., 2021). However, the specific mechanisms of *SPIB* in BC are not well characterized.

In the immune microenvironment analysis, significant differences were observed in the infiltration abundance of 25 immune cell subtypes between the high- and low-risk groups (adjusted *p* < 0.05). The CIBERSORT algorithm independently verified the differential infiltration of monocytes and eosinophils between the two groups, supporting the robustness of the ssGSEA results. Among the six prognostic genes, *CXCL13*, *IL12B*, *LTB* and *SPIB* were significantly positively correlated with most differentially infiltrated immune cells, whereas *TFF1* and *ZMYND10* showed negative correlations with these immune cells. These findings suggest that these genes may participate in the regulation of the tumor immune microenvironment through distinct mechanisms. In addition, the NETs marker gene *ELANE* was significantly negatively correlated with the risk score (*r* = −0.162, adjusted p < 0.001), providing direct evidence for the association between the risk model and neutrophil-mediated NET biology (Papayannopoulos, 2018). Notably, given the well-defined immunomodulatory functions of *CXCL13*, *IL12B*, *LTB* and *SPIB*, the six-gene prognostic signature may partly reflect the characteristics of tumor immune infiltration rather than merely intrinsic tumor biology. By contrast, *TFF1* and *ZMYND10* are involved in intrinsic tumor processes such as epithelial integrity maintenance and cell cycle regulation, respectively (Wang et al., 2019; Burclaff and Mills, 2018). This indicates that the model integrates biological information from two dimensions: immune regulation and tumor intrinsic properties, which may help broaden the scope of its prognostic prediction performance. The above findings require further validation in independent cohorts, and the underlying molecular mechanisms remain to be elucidated by functional experiments.

In this study, we identified prognostic genes (ZMYND10, IL12B, CXCL13, TFF1, LTB, SPIB) associated with NETosis and inflammation in BC and constructed a prognostic model for BC. We examined the expression of these prognostic genes across various tissues, offering valuable insights for designing BC therapies and clinical trials, and expanding the scope for developing novel treatments. Nevertheless, several limitations of the present study should be acknowledged. First, this is a retrospective study relying on data from public databases, which carries inherent batch effects and platform heterogeneity. Second, the small sample size of the validation cohort compromised the statistical power for further stratified analyses. Third, LTB was excluded from RT-qPCR validation due to limited tissue sample availability. Fourth, all findings regarding immune infiltration only demonstrated correlations derived from bulk transcriptomic data, and functional experiments (such as gene knockdown/overexpression and *in vivo* animal models) are required to validate the underlying biological mechanisms. Fifth, the model lacks prospective multicenter clinical validation. Sixth, the current model was constructed solely within the conventional Cox proportional hazards framework without machine learning survival algorithms including random survival forests and XGBoost; consequently, it may fail to fully capture nonlinear gene-gene interactions and complex dose-response relationships, partially restricting further improvements in predictive performance. Finally, direct application of the RNA-seq-derived risk formula to microarray data may introduce cross-platform technical noise. Nevertheless, the significant survival disparities observed across the two GEO cohorts sufficiently verify the cross-platform robustness of this formula.

In summary, this study offers a novel perspective and potential biomarkers for BC prognostic research. Despite the aforementioned limitations, further in-depth investigations will facilitate the clinical translation of prognostic models for BC.

## 5 Conclusion

This study identified 1,819 DEGs between Tumor and Normal groups in the TCGA-BRCA dataset, of which 1,127 were upregulated and 692 downregulated. Through WGCNA, PPI network construction, and multi-step regression analyses, six prognostic genes (ZMYND10, IL12B, CXCL13, TFF1, LTB, SPIB) were identified and used to construct a robustrisk model. The model demonstrated strong predictive performance (AUC ≥0.6 for 3-, 5-, and 7-year survival) and was validated in independent datasets (GSE42568 and GSE20685).risk score, age, N stage, and M stage were confirmed as independent prognostic factors. Significant differences in immune cell infiltration, clinical staging, and drug sensitivity were observed between HRG and LRG. By integrating NETs and IRGs within a bioinformatic framework, this study yields novel insights into BC prognosis and candidate therapeutic targets, yet further intensive research is warranted. Future investigations should systematically validate the biological functions of prognostic genes (particularly LTB) via *in vitro* and *in vivo* experiments; experimentally verify the predicted lncRNA-miRNA-mRNA regulatory networks; conduct large-scale prospective multicenter cohort studies to confirm their clinical utility; deploy single-cell and spatial transcriptomic techniques to dissect cell-type-specific expression patterns; explore the pharmacogenomic implications underlying the correlation between metformin and risk scores; and combine the identified gene signature with artificial intelligence-based multi-omics analytical pipelines. Moreover, the present prognostic signature can be integrated with machine learning survival algorithms including random survival forests and XGBoost, as well as multi-omics data (e.g., mutation, methylation, copy number variation), to capture nonlinear effects and gene-gene interactions, thereby further boosting predictive accuracy and advancing clinical translation.

## Data Availability

The datasets presented in this study can be found in online repositories. The names of the repository/repositories and accession number(s) can be found in the article/Supplementary Material.
