# Supplementary material for: Identification of NETs and inflammation-related prognostic genes in breast cancer and PCR experimental validation
Source: Front Genet. 2026 Aug 21;17:1900298. doi: 10.3389/fgene.2026.1900298 (PMC13541293; doi:10.3389/fgene.2026.1900298)
Supplement: Supplementary file 1 [file DataSheet1.pdf]

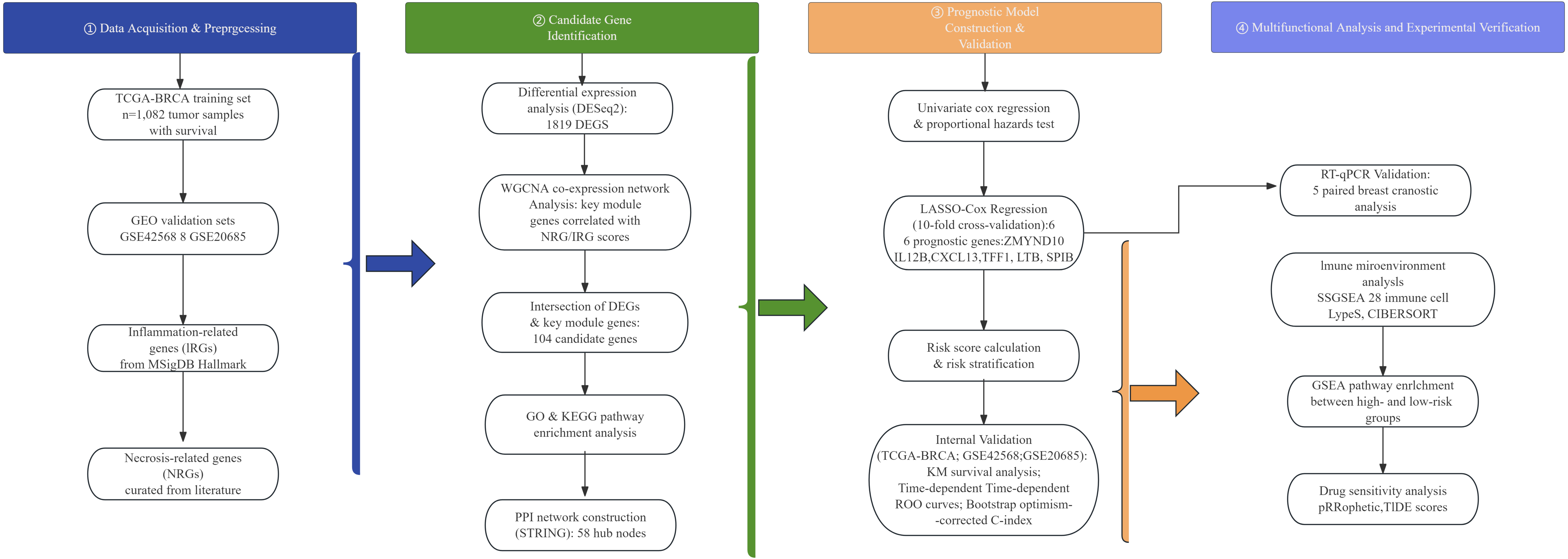
① Data Acquisition & Preprgcessing
TCGA-BRCA training set n=1,082 tumor samples with survival
GEO validation sets GSE42568 8 GSE20685
Inflammation-related genes (IRGs) from MSigDB Hallmark
Necrosis-related genes (NRGs) curated from literature
② Candidate Gene Identification
Differential expression analysis (DESeq2): 1819 DEGS
WGCNA co-expression network Analysis: key module genes correlated with NRG/IRG scores
Intersection of DEGs & key module genes: 104 candidate genes
GO & KEGG pathway enrichment analysis
PPI network construction (STRING): 58 hub nodes
③ Prognostic Model Construction & Validation
Univariate cox regression & proportional hazards test
LASSO-Cox Regression (10-fold cross-validation):6 6 prognostic genes:ZMYND10 IL12B,CXCL13,TFF1, LTB, SPIB
Risk score calculation & risk stratification
Internal Validation (TCGA-BRCA; GSE42568;GSE20685): KM survival analysis; Time-dependent Time-dependent ROO curves; Bootstrap optimism--corrected C-index
④ Multifunctional Analysis and Experimental Verification
RT-qPCR Validation: 5 paired breast cranostic analysis
lmune miroenvironment analysls SSGSEA 28 immune cell LypeS, CIBERSORT
GSEA pathway enrlchment between high- and low-risk groups
Drug sensitivity analysis pRRophetic,TIDE scores
