# Supplementary material for: Identification of NETs and inflammation-related prognostic genes in breast cancer and PCR experimental validation
Source: Front Genet. 2026 Aug 21;17:1900298. doi: 10.3389/fgene.2026.1900298 (PMC13541293; doi:10.3389/fgene.2026.1900298)
Supplement: Supplementary file 2 [file DataSheet2.zip › Supplementary Table0713/supplementary table 3.docx]

|  | Temperature | Time |
| --- | --- | --- |
| Pre-denaturation | 95℃ | 1min |
| Denaturation | 95℃ | 20s |
| Annealing | 55℃ | 20s |
| Extension | 72℃ | 30s |
