# Supplementary material for: Identification of NETs and inflammation-related prognostic genes in breast cancer and PCR experimental validation
Source: Front Genet. 2026 Aug 21;17:1900298. doi: 10.3389/fgene.2026.1900298 (PMC13541293; doi:10.3389/fgene.2026.1900298)
Supplement: Supplementary file 2 [file DataSheet2.zip › Supplementary Table0713/supplementary table 15.docx]

| drug | riskScore | r | p | ci_lower | ci_upper | p.adjust | change |
| --- | --- | --- | --- | --- | --- | --- | --- |
| Metformin | riskScore | 0.316361815394791 | 1.41339679066541e-26 | 0.261699199431857 | 0.369001342952763 | 1.95048757111827e-24 | Positive correlation |
