# Supplementary material for: Identification of NETs and inflammation-related prognostic genes in breast cancer and PCR experimental validation
Source: Front Genet. 2026 Aug 21;17:1900298. doi: 10.3389/fgene.2026.1900298 (PMC13541293; doi:10.3389/fgene.2026.1900298)
Supplement: Supplementary file 2 [file DataSheet2.zip › Supplementary Table0713/supplementary table 14.docx]

| drug | riskScore | r | p | ci_lower | ci_upper | p.adjust | change |
| --- | --- | --- | --- | --- | --- | --- | --- |
| A.443654 | riskScore | -0.025239993 | 0.406871898362485 | -0.084709298 | 0.0344084909868559 | 0.534745923562123 | NOT |
| A.770041 | riskScore | 0.0335284493447366 | 0.270499906120898 | -0.026120471 | 0.0929394653270336 | 0.392936705733515 | NOT |
| ABT.263 | riskScore | -0.140980557 | 3.23524303651675e-06 | -0.198906079 | -0.08207341 | 1.53952944496314e-05 | NOT |
| ABT.888 | riskScore | 0.0712000467638413 | 0.0191651022055966 | 0.011652766524069 | 0.130244109579141 | 0.0388938838878283 | NOT |
| AG.014699 | riskScore | 0.13387620307487 | 9.93851781069756e-06 | 0.0748768882712272 | 0.191941508042584 | 4.19464417488136e-05 | NOT |
| AICAR | riskScore | -0.00295232 | 0.922725677711578 | -0.062538043 | 0.0566543741997741 | 0.936295172972043 | NOT |
| AKT.inhibitor.VIII | riskScore | 0.0851998888728796 | 0.00504093025220189 | 0.0257338295486009 | 0.144065105234244 | 0.0124222924072118 | NOT |
| AMG.706 | riskScore | 0.12428565577475 | 4.14219007590557e-05 | 0.0651716571219359 | 0.182530376257738 | 0.000139420056213407 | NOT |
| AP.24534 | riskScore | 0.114176863862329 | 0.000167430944531902 | 0.0549540762690942 | 0.172599127784328 | 0.000502292833595706 | NOT |
| AS601245 | riskScore | 0.231344121153324 | 1.30074572353485e-14 | 0.174148440749338 | 0.286984096621144 | 4.48757274619522e-13 | NOT |
| ATRA | riskScore | 0.104207575 | 0.00059657114820289 | 0.0448896329325498 | 0.16279328524349 | 0.00158320804715382 | NOT |
| AUY922 | riskScore | 0.189378054409631 | 3.40625126024473e-10 | 0.131262801042308 | 0.246196134309692 | 4.27329703557975e-09 | NOT |
| Axitinib | riskScore | 0.133417479006918 | 1.06651093668039e-05 | 0.0744124241167273 | 0.191491608619009 | 4.32877968417334e-05 | NOT |
| AZ628 | riskScore | 0.0131851647236275 | 0.664849670398902 | -0.04644806 | 0.0727247444576574 | 0.735259164642751 | NOT |
| AZD.0530 | riskScore | -0.039949724 | 0.189148498879008 | -0.099310006 | 0.019693890723648 | 0.290027698281145 | NOT |
| AZD.2281 | riskScore | 0.1253716428259 | 3.54173513835926e-05 | 0.0662700692147058 | 0.18359658328939 | 0.000122189862273394 | NOT |
| AZD6244 | riskScore | 0.0976738432468062 | 0.00129610202035076 | 0.038300063192621 | 0.156360389266665 | 0.00337475620393216 | NOT |
| AZD6482 | riskScore | 0.0202989923554562 | 0.504767481905539 | -0.039345332 | 0.0797991813211601 | 0.617562372429802 | NOT |
| AZD7762 | riskScore | -0.080927284 | 0.00773793840714497 | -0.139849516 | -0.021433933 | 0.0180161843200867 | NOT |
| AZD8055 | riskScore | 0.173908008436192 | 8.52141721108894e-09 | 0.115508451922708 | 0.231109436122502 | 7.34972234456421e-08 | NOT |
| BAY.61.3606 | riskScore | 0.0220368349941544 | 0.468989237793207 | -0.037609284 | 0.0815264906645519 | 0.588368316504205 | NOT |
| Bexarotene | riskScore | 0.0445808735064759 | 0.142793680279118 | -0.015055854 | 0.103901546480878 | 0.22392645316498 | NOT |
| BI.2536 | riskScore | -0.007784843 | 0.798120173468253 | -0.067350322 | 0.0518359327402523 | 0.82194465625835 | NOT |
| BIBW2992 | riskScore | -0.018308479 | 0.547447641134928 | -0.077820293 | 0.0413333474627156 | 0.640666827428512 | NOT |
| Bicalutamide | riskScore | -0.059188293 | 0.0516088540718031 | -0.118367487 | 0.000409878975409515 | 0.0901521754672003 | NOT |
| BI.D1870 | riskScore | 0.0239697890655095 | 0.430896490921629 | -0.035677904 | 0.0834473091394117 | 0.555735661188643 | NOT |
| BIRB.0796 | riskScore | 0.0266365235481457 | 0.381400802013199 | -0.033012609 | 0.0860965758768381 | 0.511003016289529 | NOT |
| Bleomycin | riskScore | 0.163618902336104 | 6.22082869288617e-08 | 0.105046500312502 | 0.221060038834416 | 4.7693019978794e-07 | NOT |
| BMS.509744 | riskScore | 0.0730182037706724 | 0.0162934588063763 | 0.0134801382551755 | 0.132040337281542 | 0.0335596614220886 | NOT |
| BMS.536924 | riskScore | 0.0497618512729001 | 0.101844531250683 | -0.009864129 | 0.109035217998866 | 0.165347591912873 | NOT |
| BMS.708163 | riskScore | -0.02701751 | 0.374625456257373 | -0.086474998 | 0.0326317585209667 | 0.506846205524681 | NOT |
| BMS.754807 | riskScore | 0.012206737160066 | 0.688363867473433 | -0.04742449 | 0.0717512657402963 | 0.753922331042332 | NOT |
| Bortezomib | riskScore | 0.0808026156413577 | 0.00783312361742901 | 0.0213085019493233 | 0.139726479112985 | 0.0180161843200867 | NOT |
| Bosutinib | riskScore | 0.109019995563574 | 0.000327336585288493 | 0.0497464838475207 | 0.167528251475798 | 0.000941092682704418 | NOT |
| Bryostatin.1 | riskScore | 0.150360987649032 | 6.74746518537379e-07 | 0.0915849562100501 | 0.208092987979023 | 3.58134690608301e-06 | NOT |
| BX.795 | riskScore | 0.0225845035776058 | 0.458011367344262 | -0.037062107 | 0.0820707657103224 | 0.579867602692736 | NOT |
| Camptothecin | riskScore | 0.118769797976066 | 8.99782742492429e-05 | 0.0595949017372714 | 0.17711286783626 | 0.00028220458741808 | NOT |
| CCT007093 | riskScore | 0.181791141235199 | 1.71066212590069e-09 | 0.123532788543233 | 0.238800663683669 | 1.68622409553068e-08 | NOT |
| CCT018159 | riskScore | 0.21343460440231 | 1.29861279801379e-12 | 0.155819907903809 | 0.269601992365201 | 2.56012237322718e-11 | NOT |
| CEP.701 | riskScore | 0.066753114660209 | 0.0281153717455485 | 0.00718497209439944 | 0.125849179313974 | 0.0531496068614479 | NOT |
| CGP.082996 | riskScore | -0.159418919 | 1.3525764330947e-07 | -0.216954395 | -0.100779685 | 8.48434308032128e-07 | NOT |
| CGP.60474 | riskScore | -0.015826445 | 0.603048083354931 | -0.075352098 | 0.0438116050629867 | 0.687773847132071 | NOT |
| CHIR.99021 | riskScore | 0.00970086824540166 | 0.749926126998359 | -0.049924722 | 0.0692575539877886 | 0.802246554463361 | NOT |
| CI.1040 | riskScore | -0.011606416 | 0.702943321170965 | -0.071153925 | 0.0480235282383086 | 0.763828175760576 | NOT |
| Cisplatin | riskScore | 0.0272061985337171 | 0.371297566272344 | -0.032443131 | 0.0866624103736025 | 0.506846205524681 | NOT |
| CMK | riskScore | -0.178617189 | 3.29385083207065e-09 | -0.235704837 | -0.120301068 | 3.030342765505e-08 | NOT |
| Cyclopamine | riskScore | 0.00905300090378295 | 0.766123583895758 | -0.05057101 | 0.0686127086467424 | 0.813191371261266 | NOT |
| Cytarabine | riskScore | 0.151448272537932 | 5.59080915008234e-07 | 0.0926881327519912 | 0.209157183491019 | 3.08612665084545e-06 | NOT |
| Dasatinib | riskScore | 0.0186529930523382 | 0.539934961599617 | -0.040989299 | 0.0781628290750512 | 0.640666827428512 | NOT |
| DMOG | riskScore | 0.00882126581611275 | 0.771942533588593 | -0.050802168 | 0.0683820424108774 | 0.813191371261266 | NOT |
| Docetaxel | riskScore | 0.0545437013018653 | 0.0729075221134649 | -0.005069504 | 0.113770601924817 | 0.121667505067916 | NOT |
| Doxorubicin | riskScore | 0.18608112970391 | 6.92554865583579e-10 | 0.127902823477363 | 0.242983214401002 | 7.35173626542568e-09 | NOT |
| EHT.1864 | riskScore | 0.188486326196996 | 4.13217252789235e-10 | 0.130353887004502 | 0.24532725028488 | 4.7519984070762e-09 | NOT |
| Elesclomol | riskScore | 0.162346632938295 | 7.88764961240638e-08 | 0.103753758261161 | 0.219816561938707 | 5.37908186661617e-07 | NOT |
| Embelin | riskScore | 0.0202538522322958 | 0.50571558226326 | -0.039390421 | 0.0797543100365222 | 0.617562372429802 | NOT |
| Epothilone.B | riskScore | 0.0349065635685832 | 0.251286228262606 | -0.024741633 | 0.0943071012146404 | 0.370864329558179 | NOT |
| Erlotinib | riskScore | 0.113549998756397 | 0.000181922057314377 | 0.0543208731102764 | 0.171982881097508 | 0.000534154125731574 | NOT |
| Etoposide | riskScore | 0.125717379223563 | 3.3685776980579e-05 | 0.0666197913295445 | 0.183935993744017 | 0.000119195826238972 | NOT |
| FH535 | riskScore | 0.108619967865632 | 0.000344403456335102 | 0.0493426560397209 | 0.167134764902356 | 0.000950553539484881 | NOT |
| FTI.277 | riskScore | 0.172265315303689 | 1.18002241861139e-08 | 0.11383729375304 | 0.229505831725938 | 9.57900551578659e-08 | NOT |
| GDC.0449 | riskScore | 0.148857077896236 | 8.73276281667391e-07 | 0.0900593032020809 | 0.20662078965707 | 4.4634121063e-06 | NOT |
| GDC0941 | riskScore | 0.108839397177859 | 0.00033494139520471 | 0.0495641674466041 | 0.167350608485055 | 0.000943304337515307 | NOT |
| Gefitinib | riskScore | 0.0790580375089414 | 0.00927944123897995 | 0.0195534391934782 | 0.138004541583902 | 0.0206542401770844 | NOT |
| Gemcitabine | riskScore | 0.0664623611114078 | 0.0288091712245582 | 0.00689293731269316 | 0.125561744984564 | 0.0537252112025546 | NOT |
| GNF.2 | riskScore | -0.000681767 | 0.982128895578342 | -0.060276045 | 0.0589173527491917 | 0.982128895578342 | NOT |
| GSK269962A | riskScore | 0.134046759796251 | 9.68060583907619e-06 | 0.0750495857243346 | 0.192108777477669 | 4.19464417488136e-05 | NOT |
| GSK.650394 | riskScore | 0.133816284653257 | 1.00306708529772e-05 | 0.0748162186274383 | 0.19188274367945 | 4.19464417488136e-05 | NOT |
| GW.441756 | riskScore | 0.0415844029762337 | 0.171663582341814 | -0.018057074 | 0.100930992578319 | 0.266174992844611 | NOT |
| GW843682X | riskScore | 0.0217119397866187 | 0.475569754313085 | -0.037933871 | 0.0812035918475691 | 0.591248883740592 | NOT |
| Imatinib | riskScore | 0.0693485249062714 | 0.0225339879500666 | 0.00979226951423269 | 0.128414521050007 | 0.0450679759001332 | NOT |
| IPA.3 | riskScore | 0.152527704087461 | 4.63264350336662e-07 | 0.0937834838916444 | 0.21021355723848 | 2.66377001443581e-06 | NOT |
| JNJ.26854165 | riskScore | 0.0685353964002178 | 0.0241689528580191 | 0.00897532969814775 | 0.127610897872602 | 0.0469762745691075 | NOT |
| JNK.9L | riskScore | -0.031111231 | 0.306577314268355 | -0.090540085 | 0.0285384088793974 | 0.436161539886938 | NOT |
| JNK.Inhibitor.VIII | riskScore | -0.090380317 | 0.00292411719457183 | -0.149173539 | -0.030950301 | 0.00733687587001658 | NOT |
| JW.7.52.1 | riskScore | 0.0353497988429644 | 0.245313804511478 | -0.024298117 | 0.0947469187541129 | 0.367970706767217 | NOT |
| KIN001.135 | riskScore | -0.008553475 | 0.778682813051793 | -0.068115479 | 0.0510692838684612 | 0.814077486372329 | NOT |
| KU.55933 | riskScore | 0.0689569757834039 | 0.0233088917771142 | 0.00939887519105715 | 0.128027558750026 | 0.0459518152177394 | NOT |
| Lapatinib | riskScore | 0.0817288360236523 | 0.00715028373787791 | 0.0222404378007747 | 0.140640534480742 | 0.0173112132601255 | NOT |
| Lenalidomide | riskScore | 0.0454027079460415 | 0.135565608192416 | -0.014232529 | 0.104716087934539 | 0.217535510820388 | NOT |
| LFM.A13 | riskScore | 0.15752416153512 | 1.90761581336192e-07 | 0.098855483026554 | 0.215101530940894 | 1.14456948801715e-06 | NOT |
| Metformin | riskScore | 0.316361815394791 | 1.41339679066541e-26 | 0.261699199431857 | 0.369001342952763 | 1.95048757111827e-24 | Positive correlation |
| Methotrexate | riskScore | 0.0744936274058732 | 0.0142479811508034 | 0.0149633319562144 | 0.133497680567867 | 0.0302495599817058 | NOT |
| MG.132 | riskScore | 0.127630032081081 | 2.5468131796569e-05 | 0.0685547560177694 | 0.185813399958147 | 9.31511686773082e-05 | NOT |
| Midostaurin | riskScore | 0.0549754644484087 | 0.0706645629272858 | -0.004636453 | 0.114198037133129 | 0.121667505067916 | NOT |
| Mitomycin.C | riskScore | 0.122532570647454 | 5.31929136202421e-05 | 0.0633988167435694 | 0.180808932475854 | 0.000174776716180795 | NOT |
| MK.2206 | riskScore | 0.21367043194186 | 1.22537117056234e-12 | 0.15606099544473 | 0.269831113133045 | 2.56012237322718e-11 | NOT |
| MS.275 | riskScore | -0.013137173 | 0.665995620147419 | -0.072676998 | 0.0464959563531367 | 0.735259164642751 | NOT |
| Nilotinib | riskScore | -0.013948379 | 0.646732115994375 | -0.073484019 | 0.045686325748426 | 0.725601886237592 | NOT |
| NSC.87877 | riskScore | -0.16214705 | 8.18555936224199e-08 | -0.219621479 | -0.103550982 | 5.37908186661617e-07 | NOT |
| NU.7441 | riskScore | -0.00213078 | 0.944186946012677 | -0.061719668 | 0.0574732450916176 | 0.951078821531018 | NOT |
| Nutlin.3a | riskScore | 0.209201825000496 | 3.63914946267228e-12 | 0.151493885666499 | 0.265488508569382 | 5.58002917609749e-11 | NOT |
| NVP.BEZ235 | riskScore | 0.256767777432156 | 9.44151321633308e-18 | 0.200235153357995 | 0.311596295517538 | 4.34309607951321e-16 | NOT |
| NVP.TAE684 | riskScore | 0.0163755909478015 | 0.590529860565521 | -0.043263357 | 0.0758982457544292 | 0.679109339650349 | NOT |
| Obatoclax.Mesylate | riskScore | 0.00815544663118053 | 0.788731842116463 | -0.051466294 | 0.0677192588073723 | 0.81838341512836 | NOT |
| OSI.906 | riskScore | -0.147343183 | 1.12926867926597e-06 | -0.205138553 | -0.0885238 | 5.56568134781085e-06 | NOT |
| PAC.1 | riskScore | 0.0602912941882196 | 0.0473987833743085 | 0.000697072580213392 | 0.119458785513447 | 0.0849484689046049 | NOT |
| Paclitaxel | riskScore | 0.0594464870731737 | 0.0505963210288661 | -0.000150773 | 0.118622954075095 | 0.0895165679741478 | NOT |
| Parthenolide | riskScore | -0.054516807 | 0.0730491397734853 | -0.113743976 | 0.00509647818650869 | 0.121667505067916 | NOT |
| Pazopanib | riskScore | 0.0774977712833893 | 0.0107697249364167 | 0.0179841066164137 | 0.136464222093764 | 0.0232222193941484 | NOT |
| PD.0325901 | riskScore | 0.00674088874441734 | 0.824719134389207 | -0.05287708 | 0.0663109756150954 | 0.843046226264523 | NOT |
| PD.0332991 | riskScore | 0.280682294714123 | 4.8742022316236e-21 | 0.224846747670864 | 0.334680569495073 | 3.36319953982028e-19 | NOT |
| PD.173074 | riskScore | 0.0153276390686317 | 0.614520973220536 | -0.044309563 | 0.0748559861426478 | 0.695113887741261 | NOT |
| PF.02341066 | riskScore | 0.052082963037093 | 0.0868253841536649 | -0.007537158 | 0.111334111144348 | 0.142641702538164 | NOT |
| PF.4708671 | riskScore | -0.034808752 | 0.252617731728035 | -0.094210041 | 0.0248395034736193 | 0.370864329558179 | NOT |
| PF.562271 | riskScore | 0.0447967219233088 | 0.140867282587641 | -0.014839622 | 0.104115487157317 | 0.223444655139017 | NOT |
| PHA.665752 | riskScore | 0.0634734691957571 | 0.0368363213693967 | 0.00389146356287861 | 0.122606398669666 | 0.06777883131969 | NOT |
| PLX4720 | riskScore | 0.0322676047084246 | 0.288938834385562 | -0.027381778 | 0.0916880109690952 | 0.415349574429245 | NOT |
| Pyrimethamine | riskScore | 0.0377884549194708 | 0.214232310148037 | -0.021857496 | 0.0971663559625225 | 0.324879767037682 | NOT |
| QS11 | riskScore | 0.0282416172790063 | 0.353363713676423 | -0.031407972 | 0.0876907505847779 | 0.492567600882287 | NOT |
| Rapamycin | riskScore | 0.08117084157782 | 0.00755496131775967 | 0.0216789877332134 | 0.140089880781887 | 0.0179755976181178 | NOT |
| RDEA119 | riskScore | 0.0297991552033056 | 0.327436226192039 | -0.029850584 | 0.0892374019409073 | 0.461083665454096 | NOT |
| RO.3306 | riskScore | -0.054492593 | 0.0731768327582395 | -0.113720004 | 0.00512076337371856 | 0.121667505067916 | NOT |
| Roscovitine | riskScore | 0.16273894939589 | 7.33231769284444e-08 | 0.104152366547822 | 0.220200019867662 | 5.32557811375017e-07 | NOT |
| Salubrinal | riskScore | 0.0625771748678305 | 0.0395865760104504 | 0.00299160544156037 | 0.1217199593926 | 0.0718808880189757 | NOT |
| SB.216763 | riskScore | 0.024790316194058 | 0.415284390221263 | -0.03485791 | 0.0842625511772992 | 0.54065326274089 | NOT |
| SB590885 | riskScore | 0.027811758259379 | 0.360741604218349 | -0.031837739 | 0.0872638456340682 | 0.497823413821321 | NOT |
| Shikonin | riskScore | 0.127794241712741 | 2.48593432320445e-05 | 0.0687209019377159 | 0.185974563711403 | 9.31511686773082e-05 | NOT |
| SL.0101.1 | riskScore | 0.0198313255222858 | 0.514635310358169 | -0.039812454 | 0.0793342880528927 | 0.617562372429802 | NOT |
| Sorafenib | riskScore | -0.02350433 | 0.439901827767246 | -0.082984812 | 0.0361430252665148 | 0.562096779924814 | NOT |
| S.Trityl.L.cysteine | riskScore | -0.010786912 | 0.723022373927115 | -0.07033842 | 0.0488412124567399 | 0.779508496890171 | NOT |
| Sunitinib | riskScore | -0.018079965 | 0.552459075826036 | -0.077593084 | 0.0415615446168182 | 0.640666827428512 | NOT |
| Temsirolimus | riskScore | 0.196619714323081 | 6.85157950421208e-11 | 0.138647646064796 | 0.253248897573291 | 9.45517971581267e-10 | NOT |
| Thapsigargin | riskScore | -0.11450138 | 0.000160362176377122 | -0.172918129 | -0.055281892 | 0.00049177734088984 | NOT |
| Tipifarnib | riskScore | 0.226853460443499 | 4.28262073077857e-14 | 0.169548989614649 | 0.282629119176791 | 1.18200332169488e-12 | NOT |
| TW.37 | riskScore | 0.212737599218334 | 1.54107371381324e-12 | 0.155107396531152 | 0.268924772349545 | 2.65835215632785e-11 | NOT |
| Vinblastine | riskScore | 0.134689303161119 | 8.765135805695e-06 | 0.0757002257133088 | 0.192738906258807 | 4.0319624706197e-05 | NOT |
| Vinorelbine | riskScore | 0.0794413543617089 | 0.00894268361086946 | 0.0199390282836162 | 0.138382914416407 | 0.0202309891524588 | NOT |
| Vorinostat | riskScore | 0.130498821901993 | 1.66186039535802e-05 | 0.0714578448788122 | 0.188628527077165 | 6.55247813026878e-05 | NOT |
| VX.680 | riskScore | 0.020042238366366 | 0.51017266317526 | -0.03960179 | 0.0795439532526049 | 0.617562372429802 | NOT |
| VX.702 | riskScore | 0.0786468503010138 | 0.00965320387735687 | 0.019139834214727 | 0.137598638739172 | 0.0211451132551627 | NOT |
| WH.4.023 | riskScore | -0.073570495 | 0.015499421110994 | -0.132585891 | -0.014035308 | 0.0324078805048056 | NOT |
| WO2009093972 | riskScore | 0.118947952258574 | 8.779675801655e-05 | 0.0597749652169473 | 0.177287900845481 | 0.000281766339681021 | NOT |
| WZ.1.84 | riskScore | -0.094588191 | 0.00184100023072245 | -0.153320627 | -0.035189835 | 0.00470477836740182 | NOT |
| X17.AAG | riskScore | 0.12758161420711 | 2.56503218096936e-05 | 0.0685057678469041 | 0.185765879576119 | 9.31511686773082e-05 | NOT |
| X681640 | riskScore | -0.068324891 | 0.0246085946287893 | -0.127402841 | -0.00876385 | 0.0471664730385129 | NOT |
| XMD8.85 | riskScore | -0.018231006 | 0.549144132346436 | -0.077743264 | 0.0414107130685222 | 0.640666827428512 | NOT |
| Z.LLNle.CHO | riskScore | -0.105659231 | 0.00049905777425683 | -0.164221861 | -0.046354395 | 0.00135039162445966 | NOT |
| ZM.447439 | riskScore | 0.0262178998246503 | 0.388931462869361 | -0.033431063 | 0.0856807498712243 | 0.516082133422805 | NOT |
