# Supplementary material for: Identification of NETs and inflammation-related prognostic genes in breast cancer and PCR experimental validation
Source: Front Genet. 2026 Aug 21;17:1900298. doi: 10.3389/fgene.2026.1900298 (PMC13541293; doi:10.3389/fgene.2026.1900298)
Supplement: Supplementary file 2 [file DataSheet2.zip › Supplementary Table0713/supplementary table 2.docx]

gene

SGK1

ACTB

ACTG1

ACTN1

ACTN4

AKT1

AKT2

ARPIN

ATG7

AZU1

C3

C3AR1

C5AR1

CAMP

CARD11

CASP1

CAT

CCDC25

CCL2

CCL3

CCL4

CCL5

CD177

CD274

CD44

CEBPB

CFTR

CLEC4E

CLEC6A

CLEC7A

CSF3

CTSC

CTSG

CXCL1

CXCL2

CXCR4

CYBB

DEFA3

DNAJB1

DNASE1

ELANE

ENO1

ENTPD4

F2RL2

F3

FCAR

FCGR2B

FGL2

GPBAR1

GSDMD

H2AX

HIF1A

HMGB1

HRG

IL12A

IL17A

IL1B

IL1RL1

IL33

IL36RN

IL5

IL6

IL8

ILK

IRAK4

IRF1

ITGAM

ITGB1

ITGB2

KCNN3

KLF2

KRT10

LCP1

LDLR

LPAR3

LTF

LYZ

MAPK1

MAPK14

MAPK3

MAPK7

MCOLN3

MFN1

MFN2

MIR146A

MIR21

MIR223

MMP9

MNDA

MPO

MTOR

MYD88

MYH9

NFE2L2

NFIL3

NFKBIA

NLRP3

NOX4

OPA1

ORAI1

P2RX1

PADI4

PARVB

PF4

PIK3CA

PKM

PROCR

PRTN3

PTAFR

RIPK1

RIPK3

S100A12

S100A8

S100A9

S1PR2

SELP

SELPLG

SIGLEC14

SOCS3

SPP1

SRC

STAT3

SUCNR1

SYK

TICAM1

TIMP1

TKT

TLR2

TLR4

TLR7

TLR8

TLR9

TNF

TNFAIP3

WASL

XIST
