# Supplementary material for: Identification of NETs and inflammation-related prognostic genes in breast cancer and PCR experimental validation
Source: Front Genet. 2026 Aug 21;17:1900298. doi: 10.3389/fgene.2026.1900298 (PMC13541293; doi:10.3389/fgene.2026.1900298)
Supplement: Supplementary file 2 [file DataSheet2.zip › Supplementary Table0713/supplementary table 13.docx]

| Gene | Spearman_r | CI_lower_95 | CI_upper_95 | P_value | P_adjust |
| --- | --- | --- | --- | --- | --- |
| ELANE | -0.162 | -0.222 | -0.098 | 8.05e-08 | 0 |
