# Supplementary material for: Identification of NETs and inflammation-related prognostic genes in breast cancer and PCR experimental validation
Source: Front Genet. 2026 Aug 21;17:1900298. doi: 10.3389/fgene.2026.1900298 (PMC13541293; doi:10.3389/fgene.2026.1900298)
Supplement: Supplementary file 2 [file DataSheet2.zip › Supplementary Table0713/supplementary table 9.docx]

|  | id | OS | OS.time | ZMYND10 | IL12B | CXCL13 | TFF1 | LTB | SPIB | riskScore_out | risk_out |
| --- | --- | --- | --- | --- | --- | --- | --- | --- | --- | --- | --- |
| 1 | GSM1045208 | 0 | 3026 | 5.632514485 | 2.312811642 | 4.175770994 | 13.11406587 | 5.957540405 | 2.919989157 | -1.707428052 | Low |
| 2 | GSM1045209 | 1 | 755 | 4.937147865 | 2.312811642 | 2.93343214 | 13.64036756 | 4.660627392 | 2.55500121 | -1.550395119 | High |
| 3 | GSM1045210 | 0 | 3014 | 6.537371648 | 2.312811642 | 3.017775841 | 13.24727569 | 6.11328979 | 2.672479949 | -1.71464452 | Low |
| 4 | GSM1045211 | 1 | 1726 | 4.927264264 | 2.312811642 | 3.224404916 | 13.66576396 | 4.232547995 | 2.55500121 | -1.56167488 | Low |
| 5 | GSM1045212 | 0 | 2225 | 4.907160535 | 2.31968188 | 7.571446314 | 3.186597574 | 6.914062037 | 3.267023474 | -1.469864077 | High |
| 6 | GSM1045213 | 1 | 285 | 4.927264264 | 2.312811642 | 5.022123789 | 7.87426866 | 8.859336635 | 4.819081441 | -1.772171577 | Low |
| 7 | GSM1045214 | 0 | 2138 | 4.907160535 | 2.370395823 | 9.405376101 | 3.08937414 | 10.93999571 | 8.188499651 | -2.287061915 | Low |
| 8 | GSM1045215 | 0 | 2449 | 4.867855236 | 2.671543523 | 7.237736326 | 11.14666011 | 8.003641554 | 3.286902502 | -1.757951601 | Low |
| 9 | GSM1045216 | 1 | 2456 | 4.70561841 | 2.334054909 | 9.341301168 | 3.443132941 | 8.952144867 | 8.873438235 | -2.375068201 | Low |
| 10 | GSM1045217 | 1 | 420 | 5.223967018 | 2.419071546 | 3.165026749 | 7.911954107 | 4.041554245 | 2.55500121 | -1.37953519 | High |
| 11 | GSM1045218 | 1 | 1110 | 4.927264264 | 2.323521997 | 2.876126984 | 3.207205361 | 4.232547995 | 2.55500121 | -1.16439098 | High |
| 12 | GSM1045219 | 1 | 488 | 4.927264264 | 2.312811642 | 6.364556781 | 4.208185632 | 6.566453946 | 3.294964421 | -1.461979479 | High |
| 13 | GSM1045220 | 0 | 2718 | 4.941245448 | 2.418442961 | 7.384375807 | 6.389245821 | 6.766013829 | 2.566594779 | -1.480349642 | High |
| 14 | GSM1045221 | 1 | 767 | 4.927264264 | 2.312811642 | 3.111586108 | 4.52468518 | 4.667411532 | 2.55500121 | -1.223101297 | High |
| 15 | GSM1045222 | 1 | 1216 | 4.927264264 | 2.383182626 | 7.154672023 | 8.180328704 | 6.974633292 | 3.838461993 | -1.724284276 | Low |
| 16 | GSM1045223 | 0 | 2462 | 5.924994354 | 2.312811642 | 11.30178154 | 12.02575146 | 4.638918648 | 2.519164745 | -1.931600268 | Low |
| 17 | GSM1045224 | 1 | 1862 | 5.554767305 | 2.312811642 | 9.577161631 | 8.388552754 | 10.48264512 | 6.086555428 | -2.234761808 | Low |
| 18 | GSM1045225 | 1 | 1223 | 4.796700411 | 2.312811642 | 4.975103374 | 3.186597574 | 6.879959385 | 3.031853906 | -1.315171347 | High |
| 19 | GSM1045226 | 0 | 3019 | 7.142913919 | 2.312811642 | 5.11499111 | 12.75915033 | 4.772090098 | 2.608939608 | -1.830838707 | Low |
| 20 | GSM1045227 | 1 | 155 | 5.474548533 | 2.312811642 | 7.024002008 | 7.290931728 | 6.104913931 | 2.56312333 | -1.545190903 | High |
| 21 | GSM1045228 | 0 | 947 | 5.691975226 | 2.312811642 | 6.022390842 | 13.98596949 | 5.246126353 | 2.555776294 | -1.766590241 | Low |
| 22 | GSM1045229 | 1 | 552 | 4.927264264 | 2.312811642 | 7.690450112 | 11.11790563 | 6.590752794 | 3.131291286 | -1.746131201 | Low |
| 23 | GSM1045230 | 0 | 2903 | 5.420482175 | 2.33913935 | 7.046498129 | 13.51087712 | 7.568699093 | 3.381604887 | -1.894500011 | Low |
| 24 | GSM1045231 | 0 | 2438 | 4.655075733 | 2.346661345 | 9.260053948 | 6.011558372 | 8.772165917 | 4.761333378 | -1.847136469 | Low |
| 25 | GSM1045232 | 0 | 1627 | 5.293230294 | 2.312811642 | 5.077530234 | 13.82094633 | 6.518362591 | 2.312811642 | -1.648812035 | Low |
| 26 | GSM1045233 | 1 | 1708 | 4.927264264 | 2.312811642 | 4.963549973 | 13.04349478 | 6.029726608 | 2.602117345 | -1.622387286 | Low |
| 27 | GSM1045234 | 0 | 2237 | 4.862293486 | 2.312811642 | 2.400573424 | 11.94540672 | 3.680524141 | 2.510841497 | -1.450197666 | High |
| 28 | GSM1045235 | 1 | 845 | 4.927264264 | 2.375424235 | 6.35378662 | 9.619870266 | 7.593613459 | 3.152225171 | -1.642129542 | Low |
| 29 | GSM1045236 | 0 | 2957 | 5.223274076 | 2.312811642 | 3.219714499 | 8.687337879 | 5.778303437 | 2.676663194 | -1.429086958 | High |
| 30 | GSM1045237 | 1 | 913 | 4.927264264 | 2.326615414 | 8.949652971 | 9.668001976 | 9.11259859 | 4.724271248 | -1.98898147 | Low |
| 31 | GSM1045238 | 1 | 138 | 7.379444029 | 2.320888843 | 11.18298132 | 10.76690972 | 4.613522901 | 2.395755038 | -2.002800679 | Low |
| 32 | GSM1045239 | 1 | 1058 | 4.927264264 | 2.312811642 | 6.820225953 | 5.962631225 | 7.73798554 | 4.09421467 | -1.666967452 | Low |
| 33 | GSM1045240 | 0 | 2759 | 5.504501505 | 2.312811642 | 5.301755618 | 3.936137078 | 7.05577868 | 3.822308896 | -1.543000936 | High |
| 34 | GSM1045241 | 0 | 2764 | 5.005079568 | 2.312811642 | 7.899459179 | 6.512105356 | 8.415312105 | 2.967044863 | -1.572954146 | Low |
| 35 | GSM1045242 | 1 | 1157 | 4.927264264 | 2.312811642 | 4.19187347 | 6.682845776 | 4.901693817 | 3.134768845 | -1.434348874 | High |
| 36 | GSM1045243 | 0 | 546 | 5.139552038 | 2.312811642 | 2.844604915 | 8.237196279 | 5.246126353 | 2.880427026 | -1.418133686 | High |
| 37 | GSM1045244 | 0 | 2582 | 5.56517719 | 2.378750331 | 5.241087273 | 9.584345888 | 5.643573437 | 2.526650357 | -1.558635044 | Low |
| 38 | GSM1045245 | 0 | 2460 | 4.907160535 | 2.312811642 | 2.473662927 | 2.555630549 | 4.571767337 | 2.66805267 | -1.139027261 | High |
| 39 | GSM1045246 | 1 | 1338 | 7.625534208 | 2.312811642 | 3.111586108 | 11.42228311 | 4.497237972 | 2.510841497 | -1.729455976 | Low |
| 40 | GSM1045247 | 0 | 2626 | 7.262320976 | 2.312811642 | 2.316218028 | 12.96508529 | 4.04465013 | 2.55500121 | -1.723231851 | Low |
| 41 | GSM1045248 | 0 | 2948 | 4.927264264 | 2.369997621 | 2.959452359 | 4.062350307 | 9.301052286 | 3.350464902 | -1.329470164 | High |
| 42 | GSM1045249 | 0 | 2416 | 7.042685118 | 2.312811642 | 2.400573424 | 12.23785987 | 3.567741462 | 2.510841497 | -1.671346501 | Low |
| 43 | GSM1045250 | 1 | 194 | 4.907160535 | 2.312811642 | 3.058574608 | 5.871945572 | 4.581071676 | 2.773967568 | -1.30077628 | High |
| 44 | GSM1045251 | 0 | 2897 | 5.874484355 | 2.312811642 | 4.355913327 | 13.68519748 | 4.97409725 | 3.378273622 | -1.825643785 | Low |
| 45 | GSM1045252 | 0 | 2899 | 4.655075733 | 2.312811642 | 2.511413197 | 4.860454897 | 6.390688554 | 2.510841497 | -1.180907266 | High |
| 46 | GSM1045253 | 0 | 2829 | 6.498120154 | 2.312811642 | 4.337783593 | 8.019981978 | 8.215619816 | 2.55500121 | -1.561448192 | Low |
| 47 | GSM1045254 | 0 | 2785 | 5.387409864 | 2.312811642 | 3.117909414 | 8.955514439 | 4.358710131 | 2.395755038 | -1.405683598 | High |
| 48 | GSM1045255 | 0 | 2740 | 5.773437457 | 2.312811642 | 2.408435651 | 9.095901511 | 5.223083187 | 2.419594857 | -1.423749564 | High |
| 49 | GSM1045256 | 0 | 1235 | 5.094029046 | 2.312811642 | 2.408435651 | 9.781152155 | 4.617701167 | 2.814929386 | -1.440958461 | High |
| 50 | GSM1045257 | 1 | 2092 | 4.927264264 | 2.312811642 | 2.400573424 | 5.59587118 | 3.990710077 | 2.466770443 | -1.218016601 | High |
| 51 | GSM1045258 | 0 | 2535 | 4.907160535 | 2.323974267 | 10.24553993 | 4.565983673 | 8.049174398 | 4.120475588 | -1.762101354 | Low |
| 52 | GSM1045259 | 0 | 2211 | 4.927264264 | 2.312811642 | 5.350048896 | 6.772115344 | 5.246126353 | 5.766974246 | -1.879612042 | Low |
| 53 | GSM1045260 | 1 | 2275 | 6.232529101 | 2.314973242 | 2.408435651 | 10.72096059 | 4.77825679 | 2.519164745 | -1.541622166 | High |
| 54 | GSM1045261 | 1 | 390 | 4.731959587 | 2.312811642 | 2.400573424 | 5.367175591 | 3.272720367 | 2.519164745 | -1.197123516 | High |
| 55 | GSM1045262 | 0 | 2184 | 7.066526982 | 2.312811642 | 5.554030353 | 12.97455742 | 3.591418084 | 2.55500121 | -1.839261542 | Low |
| 56 | GSM1045263 | 1 | 919 | 5.79628149 | 2.312811642 | 6.141492123 | 10.31184841 | 5.980455876 | 2.395755038 | -1.624729832 | Low |
| 57 | GSM1045264 | 0 | 917 | 4.927264264 | 2.312811642 | 5.687162717 | 3.300583344 | 7.043171656 | 5.972163335 | -1.800890482 | Low |
| 58 | GSM1045265 | 0 | 2758 | 5.955090473 | 2.312811642 | 2.615306508 | 14.78446819 | 4.065644206 | 2.510841497 | -1.669523383 | Low |
| 59 | GSM1045266 | 0 | 977 | 4.655075733 | 2.312811642 | 9.479657208 | 2.816067349 | 11.16801887 | 8.261610255 | -2.265682249 | Low |
| 60 | GSM1045267 | 0 | 1997 | 4.927264264 | 2.312811642 | 2.760599391 | 9.439578344 | 5.246126353 | 3.17555362 | -1.482179177 | High |
| 61 | GSM1045268 | 0 | 1854 | 4.927264264 | 2.312811642 | 5.835444293 | 8.022761642 | 5.982750434 | 2.510841497 | -1.461309517 | High |
| 62 | GSM1045269 | 0 | 1793 | 4.754794057 | 2.446614243 | 9.599807306 | 3.79904278 | 8.753664083 | 3.781161796 | -1.646264448 | Low |
| 63 | GSM1045270 | 1 | 545 | 4.927264264 | 2.312811642 | 5.043902814 | 4.171740116 | 5.246126353 | 2.576008609 | -1.295375964 | High |
| 64 | GSM1045271 | 1 | 1409 | 4.907160535 | 3.001317027 | 8.99548885 | 8.324678437 | 8.120446755 | 2.584665525 | -1.636020213 | Low |
| 65 | GSM1045272 | 0 | 2943 | 7.070486832 | 2.312811642 | 3.119934518 | 13.61996561 | 5.463203342 | 2.519164745 | -1.759869982 | Low |
| 66 | GSM1045273 | 1 | 1044 | 4.927264264 | 2.312811642 | 3.388749662 | 11.66088124 | 4.045275351 | 2.484054226 | -1.484166277 | High |
| 67 | GSM1045274 | 0 | 2489 | 5.044881186 | 2.323032927 | 6.916179275 | 5.008136605 | 5.060329365 | 2.55500121 | -1.412488797 | High |
| 68 | GSM1045275 | 1 | 1332 | 4.655075733 | 2.312811642 | 5.227058136 | 3.587254907 | 5.44301254 | 2.43121981 | -1.234135036 | High |
| 69 | GSM1045276 | 1 | 1915 | 5.145021956 | 2.312811642 | 2.408435651 | 8.092838847 | 4.232547995 | 2.587416254 | -1.349321135 | High |
| 70 | GSM1045277 | 0 | 1950 | 4.907160535 | 2.312811642 | 12.50220025 | 9.96710412 | 6.880877592 | 3.097995472 | -1.899078727 | Low |
| 71 | GSM1045278 | 1 | 1080 | 5.198340513 | 2.312811642 | 6.596144764 | 7.248132293 | 6.75869444 | 2.519164745 | -1.493803075 | High |
| 72 | GSM1045279 | 0 | 2266 | 5.71810867 | 2.312811642 | 2.849122964 | 10.4453765 | 4.264242444 | 2.395755038 | -1.480749736 | High |
| 73 | GSM1045280 | 0 | 1692 | 4.927264264 | 2.313397249 | 2.408435651 | 11.13984178 | 5.345779267 | 2.55500121 | -1.437312852 | High |
| 74 | GSM1045281 | 0 | 2302 | 4.927264264 | 3.703584733 | 8.170841381 | 9.119276814 | 6.897559981 | 2.55500121 | -1.643916758 | Low |
| 75 | GSM1045282 | 1 | 365 | 4.927264264 | 2.312811642 | 2.844604915 | 2.816067349 | 4.974895563 | 3.153240802 | -1.239263185 | High |
| 76 | GSM1045283 | 0 | 2211 | 4.854896795 | 2.746896793 | 8.967047303 | 4.904185366 | 8.469006676 | 4.951887093 | -1.851874306 | Low |
| 77 | GSM1045284 | 0 | 2344 | 4.927264264 | 2.312811642 | 2.408435651 | 8.674355643 | 4.309264743 | 2.55500121 | -1.344895427 | High |
| 78 | GSM1045285 | 0 | 1864 | 5.047142685 | 2.312811642 | 8.333876483 | 11.27314837 | 7.359422573 | 2.55500121 | -1.705899934 | Low |
| 79 | GSM1045286 | 0 | 2484 | 4.944522012 | 2.628780222 | 6.717280365 | 13.75769003 | 7.52865717 | 2.696021596 | -1.748924455 | Low |
| 80 | GSM1045287 | 1 | 526 | 4.655075733 | 2.312811642 | 2.408435651 | 4.063783801 | 3.977176754 | 2.395755038 | -1.125317337 | High |
| 81 | GSM1045288 | 0 | 2955 | 6.143873697 | 2.312811642 | 2.697328455 | 5.691609268 | 5.082818488 | 2.519164745 | -1.361543271 | High |
| 82 | GSM1045289 | 0 | 2650 | 4.927264264 | 2.312811642 | 9.367243466 | 8.833362423 | 6.030448204 | 2.510841497 | -1.638922663 | Low |
| 83 | GSM1045290 | 0 | 2384 | 4.759163405 | 2.526185905 | 5.911606909 | 9.093111325 | 6.236315488 | 2.519164745 | -1.494771277 | High |
| 84 | GSM1045291 | 0 | 2689 | 4.927264264 | 2.312811642 | 12.95948955 | 3.699736615 | 4.182314783 | 2.519164745 | -1.598761139 | Low |
| 85 | GSM1045292 | 0 | 2641 | 5.643658946 | 2.312811642 | 5.305121057 | 12.75977946 | 7.348331773 | 3.368441391 | -1.812568415 | Low |
| 86 | GSM1045293 | 0 | 423 | 4.927264264 | 2.382211174 | 3.111586108 | 2.931720857 | 5.082220597 | 3.202564201 | -1.264061862 | High |
| 87 | GSM1045294 | 0 | 1682 | 4.907160535 | 2.373335658 | 2.400573424 | 2.555630549 | 2.914748519 | 2.519164745 | -1.111940405 | High |
| 88 | GSM1045295 | 0 | 2052 | 4.927264264 | 2.312811642 | 2.408435651 | 12.25472882 | 4.50006029 | 2.586727574 | -1.481130603 | High |
| 89 | GSM1045296 | 0 | 475 | 4.655075733 | 2.312811642 | 4.093409794 | 3.186597574 | 7.093265399 | 5.876242773 | -1.689494912 | Low |
| 90 | GSM1045297 | 0 | 2243 | 5.946106225 | 2.312811642 | 2.760599391 | 12.45622776 | 4.752588555 | 2.457851841 | -1.582974167 | Low |
| 91 | GSM1045298 | 0 | 2197 | 4.655075733 | 2.312811642 | 7.837659353 | 3.509026168 | 9.45923141 | 6.90869287 | -2.016936612 | Low |
| 92 | GSM1045299 | 0 | 2576 | 4.973746158 | 2.312811642 | 2.816040658 | 7.768695272 | 4.770863938 | 2.944624782 | -1.392371059 | High |
| 93 | GSM1045300 | 0 | 2556 | 5.773646313 | 2.312811642 | 3.111586108 | 12.1932278 | 4.363881829 | 2.55500121 | -1.585075283 | Low |
| 94 | GSM1045301 | 0 | 2225 | 5.885202155 | 3.705563809 | 2.844604915 | 9.224273215 | 4.232547995 | 2.948325335 | -1.570678541 | Low |
| 95 | GSM1045302 | 1 | 2022 | 5.064015904 | 2.312811642 | 3.994907269 | 3.186597574 | 5.866876021 | 2.752800363 | -1.256256953 | High |
| 96 | GSM1045303 | 0 | 2713 | 4.927264264 | 2.333933343 | 4.317518633 | 12.74210283 | 6.750968092 | 2.55500121 | -1.57930206 | Low |
| 97 | GSM1045304 | 0 | 2790 | 4.907160535 | 2.337660645 | 5.035197594 | 7.823870095 | 5.977723181 | 2.55500121 | -1.42581488 | High |
| 98 | GSM1045305 | 0 | 2622 | 7.390288832 | 2.312811642 | 4.492621099 | 11.29429894 | 5.558199608 | 3.854250813 | -1.96249533 | Low |
| 99 | GSM1045306 | 0 | 2909 | 5.586493627 | 2.312811642 | 7.474734953 | 11.26960619 | 5.695658941 | 2.395755038 | -1.694756726 | Low |
| 100 | GSM1045307 | 0 | 2952 | 4.927264264 | 2.358588416 | 4.205570571 | 11.64446406 | 5.840821264 | 2.542213122 | -1.531295882 | High |
| 101 | GSM1045308 | 0 | 2989 | 6.724945316 | 2.312811642 | 10.17844033 | 13.34054712 | 4.220398635 | 2.55500121 | -2.014511236 | Low |
| 102 | GSM1045309 | 0 | 2105 | 4.927264264 | 3.557465954 | 2.401261791 | 11.94168189 | 4.176578876 | 2.50901053 | -1.489540976 | High |
| 103 | GSM1045310 | 1 | 927 | 4.927264264 | 2.312811642 | 2.844604915 | 12.60028856 | 3.330526419 | 2.510841497 | -1.498327363 | High |
| 104 | GSM1045311 | 0 | 2962 | 6.000485514 | 2.312811642 | 2.400573424 | 9.892420937 | 4.835454014 | 2.556993259 | -1.494238252 | High |
