# Supplementary material for: Identification of NETs and inflammation-related prognostic genes in breast cancer and PCR experimental validation
Source: Front Genet. 2026 Aug 21;17:1900298. doi: 10.3389/fgene.2026.1900298 (PMC13541293; doi:10.3389/fgene.2026.1900298)
Supplement: Supplementary file 2 [file DataSheet2.zip › Supplementary Table0713/supplementary table 5.docx]

| Raw CT Value | | |  |  | |  | |  |  |  |  |  |  |  |  |  |  |  |  |
| --- | --- | --- | --- | --- | --- | --- | --- | --- | --- | --- | --- | --- | --- | --- | --- | --- | --- | --- | --- |
| samples | | | ZMYND10 | | | | | IL12B | | | CXCL13 | | | TFF1 | | | SPIB | | |
| Control 1 | | | 26.13 | 27.58 | | 27.10 | | 27.30 | 27.89 | 27.56 | 26.79 | 26.97 | 26.82 | 22.48 | 22.30 | 22.19 | 32.42 | 30.81 | 32.17 |
| Control 2 | | | 27.25 | 27.30 | | 27.00 | | 26.31 | 26.28 | 26.74 | 26.41 | 26.40 | 26.22 | 22.07 | 22.51 | 22.97 | 32.42 | 33.17 | 34.22 |
| Control 3 | | | 26.37 | 26.50 | | 27.53 | | 26.03 | 26.17 | 25.85 | 26.82 | 26.91 | 26.88 | 22.43 | 20.76 | 22.30 | 33.14 | 31.42 | 30.86 |
| Control 4 | | | 27.36 | 28.04 | | 27.91 | | 25.50 | 25.50 | 26.04 | 26.23 | 26.57 | 27.33 | 22.53 | 22.53 | 22.22 | 32.85 | 32.47 | 32.02 |
| Control 5 | | | 28.73 | 29.62 | | 28.11 | | 27.20 | 27.56 | 27.35 | 28.02 | 26.67 | 26.74 | 22.99 | 22.96 | 22.72 | 33.19 | 32.51 | 31.80 |
| BRCA1 | | | 27.70 | 27.33 | | 27.83 | | 28.05 | 27.65 | 27.43 | 26.06 | 32.40 | 25.36 | 22.46 | 22.68 | 22.40 | 30.01 | 30.66 | 31.69 |
| BRCA2 | | | 27.59 | 27.82 | | 27.05 | | 26.93 | 27.05 | 26.97 | 26.65 | 26.99 | 26.90 | 22.42 | 21.40 | 21.12 | 29.51 | 31.08 | 32.73 |
| BRCA3 | | | 26.62 | 26.63 | | 26.82 | | 27.64 | 27.50 | 27.52 | 26.19 | 26.92 | 26.81 | 21.03 | 21.22 | 21.04 | 30.52 | 30.75 | 30.94 |
| BRCA4 | | | 27.04 | 27.28 | | 27.65 | | 26.53 | 26.75 | 26.70 | 25.91 | 24.07 | 25.20 | 21.06 | 21.99 | 21.15 | 31.37 | 31.71 | 31.04 |
| BRCA5 | | | 27.83 | 27.94 | | 27.46 | | 27.47 | 27.43 | 27.27 | 25.77 | 25.65 | 25.42 | 21.28 | 22.95 | 23.10 | 31.51 | 31.18 | 31.86 |
| H-GAPDH | | | | | GAPDH-average | |  |  |  |  |  |  |  |  |  |  |  |  |  |
| 27.81 | 27.50 | 27.48 | | | 27.5936787 | |  |  |  |  |  |  |  |  |  |  |  |  |  |
| 28.62 | 27.89 | 28.33 | | | 28.27980402 | |  |  |  |  |  |  |  |  |  |  |  |  |  |
| 27.91 | 27.64 | 28.17 | | | 27.90370344 | |  |  |  |  |  |  |  |  |  |  |  |  |  |
| 28.21 | 28.19 | 28.32 | | | 28.24293037 | |  |  |  |  |  |  |  |  |  |  |  |  |  |
| 28.04 | 27.54 | 28.55 | | | 28.04502436 | |  |  |  |  |  |  |  |  |  |  |  |  |  |
| 29.04 | 29.25 | 29.36 | | | 29.21743454 | |  |  |  |  |  |  |  |  |  |  |  |  |  |
| 28.17 | 28.55 | 28.83 | | | 28.51787872 | |  |  |  |  |  |  |  |  |  |  |  |  |  |
| 28.95 | 28.31 | 28.05 | | | 28.43637632 | |  |  |  |  |  |  |  |  |  |  |  |  |  |
| 29.34 | 29.24 | 29.22 | | | 29.26632537 | |  |  |  |  |  |  |  |  |  |  |  |  |  |
| 28.37 | 28.25 | 28.82 | | | 28.47951631 | |  |  |  |  |  |  |  |  |  |  |  |  |  |
