# Supplementary material for: Identification of NETs and inflammation-related prognostic genes in breast cancer and PCR experimental validation
Source: Front Genet. 2026 Aug 21;17:1900298. doi: 10.3389/fgene.2026.1900298 (PMC13541293; doi:10.3389/fgene.2026.1900298)
Supplement: Supplementary file 2 [file DataSheet2.zip › Supplementary Table0713/supplementary table 4.docx]

| RNA Concentration Test Result | | | | | |
| --- | --- | --- | --- | --- | --- |
| ID | Sample Name | Group | Concentration (ng/μL) | A260/A280 | A260/A230 |
| 1 | 2C | Control | 930.84 | 1.923 | 0.156 |
| 2 | 3C | Control | 802.04 | 1.3 | 0.203 |
| 3 | 4C | Control | 705.44 | 1.472 | 0.244 |
| 4 | 5C | Control | 992.56 | 1.317 | 0.248 |
| 5 | 6C | Control | 886.84 | 1.32 | 0.167 |
| 6 | 2T | BRCA | 2989.3 | 1.676 | 0.418 |
| 7 | 3T | BRCA | 220.32 | 1.447 | 0.375 |
| 8 | 4T | BRCA | 436.92 | 1.703 | 0.642 |
| 9 | 5T | BRCA | 610.16 | 1.582 | 0.591 |
| 10 | 6T | BRCA | 1688.6 | 1.671 | 1.171 |
