# Supplementary material for: Identification of NETs and inflammation-related prognostic genes in breast cancer and PCR experimental validation
Source: Front Genet. 2026 Aug 21;17:1900298. doi: 10.3389/fgene.2026.1900298 (PMC13541293; doi:10.3389/fgene.2026.1900298)
Supplement: Supplementary file 2 [file DataSheet2.zip › Supplementary Table0713/supplementary table 8.docx]

| category | subcategory | ID | Description | GeneRatio | BgRatio | pvalue | p.adjust | qvalue | geneID | Count |
| --- | --- | --- | --- | --- | --- | --- | --- | --- | --- | --- |
| Human Diseases | Endocrine and metabolic disease | hsa04934 | Cushing syndrome | 5/54 | 155/8846 | 0.00245235179495544 | 0.148654401551878 | 0.132947743374454 | 90993/51176/50940/1589/8912 | 5 |
| Human Diseases | Substance dependence | hsa05030 | Cocaine addiction | 3/54 | 49/8846 | 0.00324911020959807 | 0.148654401551878 | 0.132947743374454 | 4128/90993/2891 | 3 |
| Organismal Systems | Endocrine system | hsa04927 | Cortisol synthesis and secretion | 3/54 | 65/8846 | 0.0071925091445433 | 0.148654401551878 | 0.132947743374454 | 90993/1589/8912 | 3 |
| Organismal Systems | Endocrine system | hsa04929 | GnRH secretion | 3/54 | 65/8846 | 0.0071925091445433 | 0.148654401551878 | 0.132947743374454 | 84634/3760/8912 | 3 |
| Organismal Systems | Nervous system | hsa04728 | Dopaminergic synapse | 4/54 | 132/8846 | 0.0084211899300027 | 0.148654401551878 | 0.132947743374454 | 4128/90993/3760/2891 | 4 |
| Human Diseases | Substance dependence | hsa05031 | Amphetamine addiction | 3/54 | 69/8846 | 0.00848106571337907 | 0.148654401551878 | 0.132947743374454 | 4128/90993/2891 | 3 |
| Human Diseases | Cancer: overview | hsa05230 | Central carbon metabolism in cancer | 3/54 | 71/8846 | 0.00917299568560646 | 0.148654401551878 | 0.132947743374454 | 9123/5979/2261 | 3 |
| Environmental Information Processing | Signal transduction | hsa04010 | MAPK signaling pathway | 6/54 | 300/8846 | 0.00951778171937313 | 0.148654401551878 | 0.132947743374454 | 23542/5979/2261/786/27092/8912 | 6 |
| Organismal Systems | Endocrine system | hsa04915 | Estrogen signaling pathway | 4/54 | 139/8846 | 0.0100593204057662 | 0.148654401551878 | 0.132947743374454 | 90993/3760/8688/7031 | 4 |
| Human Diseases | Cardiovascular disease | hsa05412 | Arrhythmogenic right ventricular cardiomyopathy | 3/54 | 86/8846 | 0.0154069922462617 | 0.180869434716294 | 0.161758972085011 | 51176/786/27092 | 3 |
| Organismal Systems | Circulatory system | hsa04260 | Cardiac muscle contraction | 3/54 | 87/8846 | 0.015889455231367 | 0.180869434716294 | 0.161758972085011 | 1345/786/27092 | 3 |
| Organismal Systems | Immune system | hsa04610 | Complement and coagulation cascades | 3/54 | 88/8846 | 0.0163803950078173 | 0.180869434716294 | 0.161758972085011 | 2161/629/2155 | 3 |
| Cellular Processes | Cellular community - eukaryotes | hsa04530 | Tight junction | 4/54 | 170/8846 | 0.0197603111788239 | 0.180869434716294 | 0.161758972085011 | 113457/84612/112714/644672 | 4 |
| Organismal Systems | Environmental adaptation | hsa04713 | Circadian entrainment | 3/54 | 97/8846 | 0.0211823920701559 | 0.180869434716294 | 0.161758972085011 | 3760/8912/2891 | 3 |
| Human Diseases | Cancer: specific types | hsa05216 | Thyroid cancer | 2/54 | 37/8846 | 0.0212530074904222 | 0.180869434716294 | 0.161758972085011 | 51176/5979 | 2 |
| Organismal Systems | Endocrine system | hsa04925 | Aldosterone synthesis and secretion | 3/54 | 98/8846 | 0.0217587289884263 | 0.180869434716294 | 0.161758972085011 | 90993/1589/8912 | 3 |
| Organismal Systems | Nervous system | hsa04724 | Glutamatergic synapse | 3/54 | 116/8846 | 0.0335978417988849 | 0.262853703485394 | 0.23508087450613 | 2914/3760/2891 | 3 |
| Metabolism | Amino acid metabolism | hsa00330 | Arginine and proline metabolism | 2/54 | 50/8846 | 0.037178309929002 | 0.267097783728203 | 0.238876529966655 | 5831/4128 | 2 |
| Organismal Systems | Endocrine system | hsa04935 | Growth hormone synthesis, secretion and action | 3/54 | 122/8846 | 0.0381568262468861 | 0.267097783728203 | 0.238876529966655 | 90993/3483/2691 | 3 |
| Human Diseases | Neurodegenerative disease | hsa05016 | Huntington disease | 5/54 | 311/8846 | 0.0404121307146208 | 0.268740669252229 | 0.240345830039587 | 1345/90993/113457/112714/2891 | 5 |
