# Supplementary material for: Identification of NETs and inflammation-related prognostic genes in breast cancer and PCR experimental validation
Source: Front Genet. 2026 Aug 21;17:1900298. doi: 10.3389/fgene.2026.1900298 (PMC13541293; doi:10.3389/fgene.2026.1900298)
Supplement: Supplementary file 2 [file DataSheet2.zip › Supplementary Table0713/supplementary table 12.docx]

|  | Activated B cell | Activated CD4 T cell | Activated CD8 T cell | Activated dendritic cell | CD56bright natural killer cell | CD56dim natural killer cell | Central memory CD4 T cell | Central memory CD8 T cell |
| --- | --- | --- | --- | --- | --- | --- | --- | --- |
| CXCL13 | 0.674408722049519 | 0.57867011976139 | 0.650395890962284 | 0.556184180590165 | 0.234734236622415 | 0.00164586195632019 | 0.395406617757161 | 0.0306727139780286 |
| IL12B | 0.719770355591553 | 0.607618872371113 | 0.752072983673009 | 0.559224708854746 | 0.199022551157514 | 0.0382899419799706 | 0.457782592126641 | 0.11652872904406 |
| LTB | 0.791794057059797 | 0.516507191221865 | 0.79336846687483 | 0.63991825118959 | 0.361978292375287 | 0.148649120711846 | 0.580938876763395 | 0.0635281962663756 |
| SPIB | 0.863975682608219 | 0.61534067544294 | 0.73633672026267 | 0.622872654633254 | 0.240423491362436 | -0.022216078 | 0.481363536461553 | 0.00374386950941764 |
| TFF1 | -0.164897803 | -0.323999739 | -0.125341842 | -0.241059415 | 0.0285350595785187 | 0.233475313362466 | 0.0236659620835544 | 0.171542567253035 |
| ZMYND10 | -0.257029098 | -0.458797273 | -0.21832301 | -0.270277454 | -0.12323166 | 0.153237611862779 | -0.131446547 | 0.119757338823673 |

| Effector memory CD4 T cell | Effector memory CD8 T cell | Eosinophil | Immature B cell | Macrophage | Mast cell | MDSC | Memory B cell | Monocyte |
| --- | --- | --- | --- | --- | --- | --- | --- | --- |
| 0.339102200077047 | 0.566181405917237 | 0.156273502142586 | 0.630252473163664 | 0.37254222151619 | 0.200984666667352 | 0.55472058905313 | 0.211598715441866 | 0.316377259819148 |
| 0.395091284343885 | 0.662511923489976 | 0.34302199819666 | 0.685906598711559 | 0.438409751184999 | 0.354231456996132 | 0.583441765779417 | 0.216440910549949 | 0.310310636178338 |
| 0.337937581687104 | 0.688451045377755 | 0.298452156544753 | 0.652692517639971 | 0.55466259792322 | 0.398920779704353 | 0.683669962683518 | 0.11862589903151 | 0.506500473774723 |
| 0.411849419207316 | 0.66366694778755 | 0.349100424663497 | 0.694100870120348 | 0.447652577897774 | 0.382509645363925 | 0.605634950216722 | 0.233384449046385 | 0.380574417323201 |
| -0.193418287 | -0.162214489 | 0.170931342559664 | -0.12811148 | -0.017284766 | 0.11729747940337 | -0.176375551 | -0.102560056 | -0.083424047 |
| -0.331344168 | -0.274087754 | 0.10446013262926 | -0.217903022 | -0.114638117 | 0.0926699019172384 | -0.277163051 | -0.094389925 | -0.16346607 |

| Natural killer cell | Natural killer T cell | Neutrophil | Plasmacytoid dendritic cell | Regulatory T cell | T follicular helper cell | Type 1 T helper cell | Type 17 T helper cell |
| --- | --- | --- | --- | --- | --- | --- | --- |
| 0.34868821924576 | 0.433640795046129 | -0.005481143 | 0.197147765635642 | 0.446181412927456 | 0.49465119139446 | 0.551531801674153 | 0.22054005975425 |
| 0.454989575311884 | 0.472449714100101 | -0.004436273 | 0.278486862426034 | 0.489534075251515 | 0.590069450677676 | 0.650059209001554 | 0.206684988061704 |
| 0.498124503668833 | 0.523881514783738 | 0.00791249519391498 | 0.356280993070015 | 0.53050817959636 | 0.622923852150494 | 0.685658657465193 | 0.236831758085923 |
| 0.438698618232144 | 0.52688168523641 | -0.033355981 | 0.347773243202312 | 0.516101961166338 | 0.598065480559893 | 0.701739428336191 | 0.20879876002852 |
| 0.0445420640007225 | -0.224714189 | 0.273755094550516 | -0.007614299 | -0.199657046 | -0.179369745 | -0.166014592 | -0.029335029 |
| -0.014041131 | -0.303718242 | 0.0736625186367014 | -0.009649978 | -0.26487519 | -0.302346712 | -0.301441977 | -0.116534571 |
