# Supplementary material for: Identification of NETs and inflammation-related prognostic genes in breast cancer and PCR experimental validation
Source: Front Genet. 2026 Aug 21;17:1900298. doi: 10.3389/fgene.2026.1900298 (PMC13541293; doi:10.3389/fgene.2026.1900298)
Supplement: Supplementary file 2 [file DataSheet2.zip › Supplementary Table0713/supplementary table 11.docx]

| Cell_type | median_high | median_low | median_diff | statistic | p | p.adjust | p.adj.signif |
| --- | --- | --- | --- | --- | --- | --- | --- |
| Activated B cells | -0.209669646 | -0.195606956 | -0.014062691 | 139024 | 0.204 | 0.287 | ns |
| Activated CD4 T cells | 0.127899767108376 | 0.0937342898472723 | 0.0341654772611036 | 169034 | 4.57e-06 | 4.26533333333333e-05 | **** |
| Activated CD8 T cells | 0.215152227032388 | 0.214650138931554 | 0.00050208810083488 | 143238 | 0.653 | 0.703230769230769 | ns |
| Activated dendritic cells | 0.207993782769306 | 0.201498559646049 | 0.00649522312325762 | 157180 | 0.0232 | 0.0588 | ns |
| CD56bright natural killer cells | 0.258627789929788 | 0.26237356485883 | -0.003745775 | 135268 | 0.0451 | 0.0971384615384615 | ns |
| CD56dim natural killer cells | 0.32386929045344 | 0.330146819872427 | -0.006277529 | 137478 | 0.116 | 0.191058823529412 | ns |
| Central memory CD4 T cells | 0.451526220050441 | 0.453935450357703 | -0.00240923 | 143206 | 0.649 | 0.703230769230769 | ns |
| Central memory CD8 T cells | 0.334312319890271 | 0.336515903398554 | -0.002203584 | 135984 | 0.0623 | 0.116293333333333 | ns |
| Effector memory CD4 T cells | 0.0920377782504704 | 0.0883678234217259 | 0.00366995482874451 | 150653 | 0.319 | 0.406 | ns |
| Effector memory CD8 T cells | 0.155104391076094 | 0.146699035809065 | 0.00840535526702882 | 149791 | 0.407 | 0.495478260869565 | ns |
| Eosinophils | -0.115193083 | -0.09874767 | -0.016445412 | 126616 | 0.000222 | 0.0012432 | ** |
| Gamma delta T cells | 0.235574813107889 | 0.216312545586931 | 0.0192622675209579 | 176791 | 1.08e-09 | 3.024e-08 | **** |
| Immature B cells | -0.025557795 | -0.031802966 | 0.00624517091336014 | 152039 | 0.205 | 0.287 | ns |
| Immature dendritic cells | 0.314386776895915 | 0.317258585455405 | -0.002871809 | 138831 | 0.191 | 0.287 | ns |
| MDSCs | 0.268430034425606 | 0.247075035571017 | 0.0213549988545886 | 158386 | 0.0122 | 0.0379555555555556 | * |
| Macrophages | 0.0327518256579774 | 0.0319226177512455 | 0.000829207906731973 | 145855 | 0.951 | 0.951 | ns |
| Mast cells | 0.0187309652580914 | 0.0417348178687031 | -0.023003853 | 121142 | 1.93e-06 | 2.702e-05 | **** |
| Memory B cells | 0.0634227508358113 | 0.0673485523816541 | -0.003925802 | 139661 | 0.251 | 0.334666666666667 | ns |
| Monocytes | 0.321601072485465 | 0.317456233280779 | 0.00414483920468556 | 160299 | 0.00398 | 0.0185733333333333 | * |
| Natural killer T cells | 0.0694658328995317 | 0.0635669180666988 | 0.00589891483283292 | 157014 | 0.0252 | 0.0588 | ns |
| Natural killer cells | 0.267479242643571 | 0.273894701161469 | -0.006415459 | 133167 | 0.0158 | 0.04424 | * |
| Neutrophils | -0.219949103 | -0.220615561 | 0.0006664581911765 | 147651 | 0.681 | 0.706222222222222 | ns |
| Plasmacytoid dendritic cells | 0.361029133805818 | 0.366171626053011 | -0.005142492 | 132286 | 0.00971 | 0.03535 | * |
| Regulatory T cells | 0.162601957451263 | 0.136983844444624 | 0.0256181130066391 | 164503 | 0.000216 | 0.0012432 | ** |
| T follicular helper cells | 0.118226885010616 | 0.103973537791746 | 0.0142533472188706 | 158719 | 0.0101 | 0.03535 | * |
| Type 1 T helper cells | 0.0953971735601235 | 0.0902138514684351 | 0.00518332209168836 | 155493 | 0.0522 | 0.1044 | ns |
| Type 17 T helper cells | -0.07880708 | -0.080469536 | 0.0016624561778113 | 154213 | 0.0907 | 0.158725 | ns |
| Type 2 T helper cells | 0.11723819686818 | 0.116100631586248 | 0.00113756528193187 | 148534 | 0.559 | 0.652166666666667 | ns |
