# Supplementary material for: Identification of NETs and inflammation-related prognostic genes in breast cancer and PCR experimental validation
Source: Front Genet. 2026 Aug 21;17:1900298. doi: 10.3389/fgene.2026.1900298 (PMC13541293; doi:10.3389/fgene.2026.1900298)
Supplement: Supplementary file 2 [file DataSheet2.zip › Supplementary Table0713/supplementary table 7.docx]

| ONTOLOGY | ID | Description | GeneRatio | BgRatio | pvalue | p.adjust | qvalue | geneID | Count |
| --- | --- | --- | --- | --- | --- | --- | --- | --- | --- |
| BP | GO:0042391 | regulation of membrane potential | 10/113 | 440/18870 | 0.000313605341889873 | 0.238205619326982 | 0.221932667661103 | PYCR1/MAPK8IP2/HSH2D/KCNJ3/KCNH1/KCNK15/RIMS4/KCNE4/CACNA1H/GRIA2 | 10 |
| BP | GO:0035988 | chondrocyte proliferation | 3/113 | 25/18870 | 0.000436859086900634 | 0.238205619326982 | 0.221932667661103 | LEF1/BMPR1B/FGFR3 | 3 |
| BP | GO:0035249 | synaptic transmission | glutamatergic | 5/113 | 111/18870 | 0.000545201275942388 | 0.238205619326982 | 0.221932667661103 | GRM4/MAPK8IP2/CACNG4/SYT1/GRIA2 |
| BP | GO:0048799 | animal organ maturation | 3/113 | 33/18870 | 0.00100083233512906 | 0.238205619326982 | 0.221932667661103 | RET/FGFR3/PAX2 | 3 |
| BP | GO:0003002 | regionalization | 9/113 | 430/18870 | 0.00112634557017538 | 0.238205619326982 | 0.221932667661103 | HES6/CFAP45/LEF1/BMPR1B/DNAAF3/EMX1/PAX2/DLX2/HOXB1 | 9 |
| BP | GO:0051966 | regulation of synaptic transmission | glutamatergic | 4/113 | 79/18870 | 0.00129671885117104 | 0.238205619326982 | 0.221932667661103 | GRM4/MAPK8IP2/CACNG4/SYT1 |
| BP | GO:0021604 | cranial nerve structural organization | 2/113 | 10/18870 | 0.00155008939942294 | 0.238205619326982 | 0.221932667661103 | PAX2/HOXB1 | 2 |
| BP | GO:0090184 | positive regulation of kidney development | 2/113 | 10/18870 | 0.00155008939942294 | 0.238205619326982 | 0.221932667661103 | RET/PAX2 | 2 |
| BP | GO:0071277 | cellular response to calcium ion | 4/113 | 87/18870 | 0.00185199830838218 | 0.238205619326982 | 0.221932667661103 | CPNE7/KCNH1/SYT1/SYT9 | 4 |
| BP | GO:0031340 | positive regulation of vesicle fusion | 2/113 | 11/18870 | 0.00188715029774686 | 0.238205619326982 | 0.221932667661103 | SYT1/SYT9 | 2 |
| BP | GO:0071805 | potassium ion transmembrane transport | 6/113 | 217/18870 | 0.00196387241506056 | 0.238205619326982 | 0.221932667661103 | KCNJ3/KCNF1/HPN/KCNH1/KCNK15/KCNE4 | 6 |
| BP | GO:0007389 | pattern specification process | 9/113 | 475/18870 | 0.00222626407295148 | 0.238205619326982 | 0.221932667661103 | HES6/CFAP45/LEF1/BMPR1B/DNAAF3/EMX1/PAX2/DLX2/HOXB1 | 9 |
| BP | GO:0006705 | mineralocorticoid biosynthetic process | 2/113 | 12/18870 | 0.00225573503150551 | 0.238205619326982 | 0.221932667661103 | CYP21A2/CACNA1H | 2 |
| BP | GO:0031630 | regulation of synaptic vesicle fusion to presynaptic active zone membrane | 2/113 | 12/18870 | 0.00225573503150551 | 0.238205619326982 | 0.221932667661103 | SYT1/SYT9 | 2 |
| BP | GO:1901632 | regulation of synaptic vesicle membrane organization | 2/113 | 12/18870 | 0.00225573503150551 | 0.238205619326982 | 0.221932667661103 | SYT1/SYT9 | 2 |
| BP | GO:0008212 | mineralocorticoid metabolic process | 2/113 | 14/18870 | 0.00308594784164166 | 0.252433794217557 | 0.235188848679568 | CYP21A2/CACNA1H | 2 |
| BP | GO:0060019 | radial glial cell differentiation | 2/113 | 14/18870 | 0.00308594784164166 | 0.252433794217557 | 0.235188848679568 | LEF1/EMX1 | 2 |
| BP | GO:0007520 | myoblast fusion | 3/113 | 49/18870 | 0.00315292378349986 | 0.252433794217557 | 0.235188848679568 | KCNH1/GDF15/CACNA1H | 3 |
| BP | GO:0006813 | potassium ion transport | 6/113 | 241/18870 | 0.0033073367668621 | 0.252433794217557 | 0.235188848679568 | KCNJ3/KCNF1/HPN/KCNH1/KCNK15/KCNE4 | 6 |
| BP | GO:0006704 | glucocorticoid biosynthetic process | 2/113 | 16/18870 | 0.00403770519186099 | 0.252433794217557 | 0.235188848679568 | CYP21A2/CACNA1H | 2 |
| BP | GO:0048532 | anatomical structure arrangement | 2/113 | 16/18870 | 0.00403770519186099 | 0.252433794217557 | 0.235188848679568 | PAX2/HOXB1 | 2 |
| BP | GO:0098915 | membrane repolarization during ventricular cardiac muscle cell action potential | 2/113 | 16/18870 | 0.00403770519186099 | 0.252433794217557 | 0.235188848679568 | KCNJ3/KCNE4 | 2 |
| BP | GO:0072348 | sulfur compound transport | 3/113 | 54/18870 | 0.00415387656896723 | 0.252433794217557 | 0.235188848679568 | SLC44A4/SLC16A6/SLC7A13 | 3 |
| BP | GO:0043923 | positive regulation by host of viral transcription | 2/113 | 17/18870 | 0.00455823309741742 | 0.252433794217557 | 0.235188848679568 | LEF1/HPN | 2 |
| BP | GO:0072224 | metanephric glomerulus development | 2/113 | 17/18870 | 0.00455823309741742 | 0.252433794217557 | 0.235188848679568 | RET/PAX2 | 2 |
| BP | GO:0010755 | regulation of plasminogen activation | 2/113 | 18/18870 | 0.00510803776411821 | 0.252433794217557 | 0.235188848679568 | F12/HPN | 2 |
| BP | GO:0036003 | positive regulation of transcription from RNA polymerase II promoter in response to stress | 2/113 | 18/18870 | 0.00510803776411821 | 0.252433794217557 | 0.235188848679568 | CREB3L1/MUC1 | 2 |
| BP | GO:0072189 | ureter development | 2/113 | 18/18870 | 0.00510803776411821 | 0.252433794217557 | 0.235188848679568 | RET/PAX2 | 2 |
| BP | GO:0060350 | endochondral bone morphogenesis | 3/113 | 61/18870 | 0.00584761032712846 | 0.252433794217557 | 0.235188848679568 | BMPR1B/SCX/FGFR3 | 3 |
| BP | GO:0034765 | regulation of monoatomic ion transmembrane transport | 8/113 | 454/18870 | 0.00599082956873593 | 0.252433794217557 | 0.235188848679568 | KCNJ3/KCNF1/KCNH1/KCNK15/CACNG1/KCNE4/CACNG4/CACNA1H | 8 |
| BP | GO:0000768 | syncytium formation by plasma membrane fusion | 3/113 | 63/18870 | 0.0063967457697907 | 0.252433794217557 | 0.235188848679568 | KCNH1/GDF15/CACNA1H | 3 |
| BP | GO:0140253 | cell-cell fusion | 3/113 | 63/18870 | 0.0063967457697907 | 0.252433794217557 | 0.235188848679568 | KCNH1/GDF15/CACNA1H | 3 |
| BP | GO:1902074 | response to salt | 7/113 | 366/18870 | 0.00650870470575857 | 0.252433794217557 | 0.235188848679568 | CPNE7/KCNH1/F7/CACNG4/SYT1/CACNA1H/SYT9 | 7 |
| BP | GO:0048791 | calcium ion-regulated exocytosis of neurotransmitter | 2/113 | 21/18870 | 0.00692949876948319 | 0.252433794217557 | 0.235188848679568 | RIMS4/SYT1 | 2 |
| BP | GO:1902075 | cellular response to salt | 5/113 | 199/18870 | 0.00694990029764246 | 0.252433794217557 | 0.235188848679568 | CPNE7/KCNH1/SYT1/CACNA1H/SYT9 | 5 |
| BP | GO:2000117 | negative regulation of cysteine-type endopeptidase activity | 3/113 | 65/18870 | 0.0069756495321748 | 0.252433794217557 | 0.235188848679568 | CST2/CST5/PAX2 | 3 |
| BP | GO:0071248 | cellular response to metal ion | 5/113 | 200/18870 | 0.00709480914474389 | 0.252433794217557 | 0.235188848679568 | CPNE7/KCNH1/SYT1/CACNA1H/SYT9 | 5 |
| BP | GO:0017156 | calcium-ion regulated exocytosis | 3/113 | 66/18870 | 0.00727638539851396 | 0.252433794217557 | 0.235188848679568 | RIMS4/SYT1/SYT9 | 3 |
| BP | GO:0042446 | hormone biosynthetic process | 3/113 | 66/18870 | 0.00727638539851396 | 0.252433794217557 | 0.235188848679568 | DIO1/CYP21A2/CACNA1H | 3 |
| BP | GO:0042531 | positive regulation of tyrosine phosphorylation of STAT protein | 3/113 | 66/18870 | 0.00727638539851396 | 0.252433794217557 | 0.235188848679568 | TNFRSF18/FGFR3/IL20 | 3 |
| BP | GO:0044458 | motile cilium assembly | 3/113 | 66/18870 | 0.00727638539851396 | 0.252433794217557 | 0.235188848679568 | NEURL1/ZMYND10/DNAAF3 | 3 |
| BP | GO:0010951 | negative regulation of endopeptidase activity | 4/113 | 128/18870 | 0.00736383919974412 | 0.252433794217557 | 0.235188848679568 | CST2/CST5/SERPINA9/PAX2 | 4 |
| BP | GO:0006949 | syncytium formation | 3/113 | 67/18870 | 0.00758470503365866 | 0.252433794217557 | 0.235188848679568 | KCNH1/GDF15/CACNA1H | 3 |
| BP | GO:0014902 | myotube differentiation | 4/113 | 130/18870 | 0.00776989690371764 | 0.252433794217557 | 0.235188848679568 | CEACAM5/KCNH1/GDF15/CACNA1H | 4 |
| BP | GO:0051216 | cartilage development | 5/113 | 207/18870 | 0.00816887869874034 | 0.252433794217557 | 0.235188848679568 | MATN3/BMPR1B/SCX/FGFR3/DLX2 | 5 |
| BP | GO:0006590 | thyroid hormone generation | 2/113 | 23/18870 | 0.00828362113836319 | 0.252433794217557 | 0.235188848679568 | HPN/DIO1 | 2 |
| BP | GO:0031629 | synaptic vesicle fusion to presynaptic active zone membrane | 2/113 | 23/18870 | 0.00828362113836319 | 0.252433794217557 | 0.235188848679568 | SYT1/SYT9 | 2 |
| BP | GO:0007586 | digestion | 4/113 | 134/18870 | 0.00862564214318944 | 0.252433794217557 | 0.235188848679568 | CLPSL2/CAPN9/TFF3/TFF1 | 4 |
| BP | GO:0023061 | signal release | 8/113 | 484/18870 | 0.00866527947771161 | 0.252433794217557 | 0.235188848679568 | GRM4/TMEM132A/SLC44A4/RIMS4/SYT1/TRH/SYT9/GHRH | 8 |
| BP | GO:0030277 | maintenance of gastrointestinal epithelium | 2/113 | 24/18870 | 0.00900157620852673 | 0.252433794217557 | 0.235188848679568 | TFF3/TFF1 | 2 |
| BP | GO:0031338 | regulation of vesicle fusion | 2/113 | 24/18870 | 0.00900157620852673 | 0.252433794217557 | 0.235188848679568 | SYT1/SYT9 | 2 |
| BP | GO:0090103 | cochlea morphogenesis | 2/113 | 24/18870 | 0.00900157620852673 | 0.252433794217557 | 0.235188848679568 | HPN/PAX2 | 2 |
| BP | GO:0099500 | vesicle fusion to plasma membrane | 2/113 | 24/18870 | 0.00900157620852673 | 0.252433794217557 | 0.235188848679568 | SYT1/SYT9 | 2 |
| BP | GO:0010466 | negative regulation of peptidase activity | 4/113 | 137/18870 | 0.00930641876659768 | 0.252433794217557 | 0.235188848679568 | CST2/CST5/SERPINA9/PAX2 | 4 |
| BP | GO:0008211 | glucocorticoid metabolic process | 2/113 | 25/18870 | 0.00974633421910291 | 0.252433794217557 | 0.235188848679568 | CYP21A2/CACNA1H | 2 |
| BP | GO:0010954 | positive regulation of protein processing | 2/113 | 25/18870 | 0.00974633421910291 | 0.252433794217557 | 0.235188848679568 | F12/HPN | 2 |
| BP | GO:0086013 | membrane repolarization during cardiac muscle cell action potential | 2/113 | 25/18870 | 0.00974633421910291 | 0.252433794217557 | 0.235188848679568 | KCNJ3/KCNE4 | 2 |
| BP | GO:2000311 | regulation of AMPA receptor activity | 2/113 | 25/18870 | 0.00974633421910291 | 0.252433794217557 | 0.235188848679568 | MAPK8IP2/CACNG4 | 2 |
| BP | GO:0001707 | mesoderm formation | 3/113 | 75/18870 | 0.0103291976023257 | 0.252433794217557 | 0.235188848679568 | LEF1/SCX/PAX2 | 3 |
| BP | GO:0032967 | positive regulation of collagen biosynthetic process | 2/113 | 26/18870 | 0.0105175542913001 | 0.252433794217557 | 0.235188848679568 | CREB3L1/SCX | 2 |
| BP | GO:0060561 | apoptotic process involved in morphogenesis | 2/113 | 26/18870 | 0.0105175542913001 | 0.252433794217557 | 0.235188848679568 | LEF1/PAX2 | 2 |
| BP | GO:0090218 | positive regulation of lipid kinase activity | 2/113 | 26/18870 | 0.0105175542913001 | 0.252433794217557 | 0.235188848679568 | EEF1A2/FGFR3 | 2 |
| BP | GO:0007269 | neurotransmitter secretion | 4/113 | 144/18870 | 0.0110287052146727 | 0.252433794217557 | 0.235188848679568 | GRM4/RIMS4/SYT1/SYT9 | 4 |
| BP | GO:0051592 | response to calcium ion | 4/113 | 144/18870 | 0.0110287052146727 | 0.252433794217557 | 0.235188848679568 | CPNE7/KCNH1/SYT1/SYT9 | 4 |
| BP | GO:0099643 | signal release from synapse | 4/113 | 144/18870 | 0.0110287052146727 | 0.252433794217557 | 0.235188848679568 | GRM4/RIMS4/SYT1/SYT9 | 4 |
| BP | GO:0048332 | mesoderm morphogenesis | 3/113 | 77/18870 | 0.0110938577409011 | 0.252433794217557 | 0.235188848679568 | LEF1/SCX/PAX2 | 3 |
| BP | GO:0010714 | positive regulation of collagen metabolic process | 2/113 | 27/18870 | 0.0113148986044486 | 0.252433794217557 | 0.235188848679568 | CREB3L1/SCX | 2 |
| BP | GO:0021895 | cerebral cortex neuron differentiation | 2/113 | 27/18870 | 0.0113148986044486 | 0.252433794217557 | 0.235188848679568 | EMX1/DLX2 | 2 |
| BP | GO:0031639 | plasminogen activation | 2/113 | 27/18870 | 0.0113148986044486 | 0.252433794217557 | 0.235188848679568 | F12/HPN | 2 |
| BP | GO:0042133 | neurotransmitter metabolic process | 2/113 | 27/18870 | 0.0113148986044486 | 0.252433794217557 | 0.235188848679568 | MAOA/SLC44A4 | 2 |
| BP | GO:1903319 | positive regulation of protein maturation | 2/113 | 27/18870 | 0.0113148986044486 | 0.252433794217557 | 0.235188848679568 | F12/HPN | 2 |
| BP | GO:0042509 | regulation of tyrosine phosphorylation of STAT protein | 3/113 | 78/18870 | 0.0114881435892238 | 0.252448729521782 | 0.235202763682575 | TNFRSF18/FGFR3/IL20 | 3 |
| BP | GO:0030194 | positive regulation of blood coagulation | 2/113 | 28/18870 | 0.0121380323721305 | 0.252448729521782 | 0.235202763682575 | F12/F7 | 2 |
| BP | GO:0048499 | synaptic vesicle membrane organization | 2/113 | 28/18870 | 0.0121380323721305 | 0.252448729521782 | 0.235202763682575 | SYT1/SYT9 | 2 |
| BP | GO:0099625 | ventricular cardiac muscle cell membrane repolarization | 2/113 | 28/18870 | 0.0121380323721305 | 0.252448729521782 | 0.235202763682575 | KCNJ3/KCNE4 | 2 |
| BP | GO:1900048 | positive regulation of hemostasis | 2/113 | 28/18870 | 0.0121380323721305 | 0.252448729521782 | 0.235202763682575 | F12/F7 | 2 |
| BP | GO:0071241 | cellular response to inorganic substance | 5/113 | 229/18870 | 0.0122718132406422 | 0.252448729521782 | 0.235202763682575 | CPNE7/KCNH1/SYT1/CACNA1H/SYT9 | 5 |
| BP | GO:0048705 | skeletal system morphogenesis | 5/113 | 230/18870 | 0.0124861583295616 | 0.253565061461867 | 0.236242833711814 | BMPR1B/SCX/FGFR3/DLX2/HOXB1 | 5 |
| BP | GO:0042403 | thyroid hormone metabolic process | 2/113 | 29/18870 | 0.0129866238184829 | 0.257069716580867 | 0.239508069275901 | HPN/DIO1 | 2 |
| BP | GO:0097066 | response to thyroid hormone | 2/113 | 29/18870 | 0.0129866238184829 | 0.257069716580867 | 0.239508069275901 | HPN/F7 | 2 |
| BP | GO:0007260 | tyrosine phosphorylation of STAT protein | 3/113 | 82/18870 | 0.0131456105069761 | 0.257069716580867 | 0.239508069275901 | TNFRSF18/FGFR3/IL20 | 3 |
| BP | GO:0021602 | cranial nerve morphogenesis | 2/113 | 30/18870 | 0.0138603441546707 | 0.262423721160145 | 0.244496316498221 | PAX2/HOXB1 | 2 |
| BP | GO:0050820 | positive regulation of coagulation | 2/113 | 30/18870 | 0.0138603441546707 | 0.262423721160145 | 0.244496316498221 | F12/F7 | 2 |
| BP | GO:0060485 | mesenchyme development | 6/113 | 327/18870 | 0.0139164094554622 | 0.262423721160145 | 0.244496316498221 | LEF1/SCX/RET/HPN/LRG1/PAX2 | 6 |
| BP | GO:0060325 | face morphogenesis | 2/113 | 31/18870 | 0.0147588675555306 | 0.271837746604192 | 0.253267225371529 | LEF1/SCX | 2 |
| BP | GO:0086011 | membrane repolarization during action potential | 2/113 | 31/18870 | 0.0147588675555306 | 0.271837746604192 | 0.253267225371529 | KCNJ3/KCNE4 | 2 |
| BP | GO:0060041 | retina development in camera-type eye | 4/113 | 159/18870 | 0.0153801185432513 | 0.276000932000345 | 0.257146003897185 | BMPR1B/RET/PAX2/DLX2 | 4 |
| BP | GO:0001958 | endochondral ossification | 2/113 | 32/18870 | 0.0156818711363832 | 0.276000932000345 | 0.257146003897185 | SCX/FGFR3 | 2 |
| BP | GO:0036075 | replacement ossification | 2/113 | 32/18870 | 0.0156818711363832 | 0.276000932000345 | 0.257146003897185 | SCX/FGFR3 | 2 |
| BP | GO:0061036 | positive regulation of cartilage development | 2/113 | 32/18870 | 0.0156818711363832 | 0.276000932000345 | 0.257146003897185 | BMPR1B/SCX | 2 |
| BP | GO:0043618 | regulation of transcription from RNA polymerase II promoter in response to stress | 2/113 | 33/18870 | 0.0166290349300125 | 0.289454849770768 | 0.269680820958676 | CREB3L1/MUC1 | 2 |
| BP | GO:0010669 | epithelial structure maintenance | 2/113 | 34/18870 | 0.0176000418638123 | 0.29604993590957 | 0.275825365593579 | TFF3/TFF1 | 2 |
| BP | GO:0060074 | synapse maturation | 2/113 | 34/18870 | 0.0176000418638123 | 0.29604993590957 | 0.275825365593579 | NEURL1/SEZ6L | 2 |
| BP | GO:0090183 | regulation of kidney development | 2/113 | 34/18870 | 0.0176000418638123 | 0.29604993590957 | 0.275825365593579 | RET/PAX2 | 2 |
| BP | GO:0030509 | BMP signaling pathway | 4/113 | 167/18870 | 0.0180870735983606 | 0.29604993590957 | 0.275825365593579 | LEF1/BMPR1B/SCX/TMPRSS6 | 4 |
| BP | GO:0050731 | positive regulation of peptidyl-tyrosine phosphorylation | 4/113 | 168/18870 | 0.0184449420130903 | 0.29604993590957 | 0.275825365593579 | TNFRSF18/FGFR3/IL20/DOK7 | 4 |
| BP | GO:0086005 | ventricular cardiac muscle cell action potential | 2/113 | 35/18870 | 0.0185945777370976 | 0.29604993590957 | 0.275825365593579 | KCNJ3/KCNE4 | 2 |
| BP | GO:0045927 | positive regulation of growth | 5/113 | 256/18870 | 0.0189833533619126 | 0.29604993590957 | 0.275825365593579 | HPN/SLC44A4/SYT1/AGR2/GHRH | 5 |
| BP | GO:0032094 | response to food | 2/113 | 36/18870 | 0.0196123311985801 | 0.29604993590957 | 0.275825365593579 | CLPSL2/GHRH | 2 |
| BP | GO:0016079 | synaptic vesicle exocytosis | 3/113 | 96/18870 | 0.0199744561094059 | 0.29604993590957 | 0.275825365593579 | RIMS4/SYT1/SYT9 | 3 |
| BP | GO:0035082 | axoneme assembly | 3/113 | 96/18870 | 0.0199744561094059 | 0.29604993590957 | 0.275825365593579 | NEURL1/ZMYND10/DNAAF3 | 3 |
| BP | GO:0021675 | nerve development | 3/113 | 97/18870 | 0.020524041705341 | 0.29604993590957 | 0.275825365593579 | RET/PAX2/HOXB1 | 3 |
| BP | GO:0051952 | regulation of amine transport | 3/113 | 97/18870 | 0.020524041705341 | 0.29604993590957 | 0.275825365593579 | SYT1/TRH/SYT9 | 3 |
| BP | GO:0014059 | regulation of dopamine secretion | 2/113 | 37/18870 | 0.0206529937240069 | 0.29604993590957 | 0.275825365593579 | SYT1/SYT9 | 2 |
| BP | GO:0042755 | eating behavior | 2/113 | 37/18870 | 0.0206529937240069 | 0.29604993590957 | 0.275825365593579 | GDF15/TRH | 2 |
| BP | GO:0048854 | brain morphogenesis | 2/113 | 37/18870 | 0.0206529937240069 | 0.29604993590957 | 0.275825365593579 | EMX1/PAX2 | 2 |
| BP | GO:0060323 | head morphogenesis | 2/113 | 37/18870 | 0.0206529937240069 | 0.29604993590957 | 0.275825365593579 | LEF1/SCX | 2 |
| BP | GO:0007259 | receptor signaling pathway via JAK-STAT | 4/113 | 174/18870 | 0.0206846794959961 | 0.29604993590957 | 0.275825365593579 | TNFRSF18/RET/FGFR3/IL20 | 4 |
| BP | GO:0048762 | mesenchymal cell differentiation | 5/113 | 262/18870 | 0.0207477408708371 | 0.29604993590957 | 0.275825365593579 | LEF1/RET/HPN/LRG1/PAX2 | 5 |
| BP | GO:0010038 | response to metal ion | 6/113 | 359/18870 | 0.0210196983013086 | 0.29604993590957 | 0.275825365593579 | CPNE7/KCNH1/SYT1/CACNA1H/TFF1/SYT9 | 6 |
| BP | GO:0032965 | regulation of collagen biosynthetic process | 2/113 | 38/18870 | 0.0217162595939611 | 0.29604993590957 | 0.275825365593579 | CREB3L1/SCX | 2 |
| BP | GO:0060416 | response to growth hormone | 2/113 | 38/18870 | 0.0217162595939611 | 0.29604993590957 | 0.275825365593579 | F7/GDF15 | 2 |
| BP | GO:0086091 | regulation of heart rate by cardiac conduction | 2/113 | 38/18870 | 0.0217162595939611 | 0.29604993590957 | 0.275825365593579 | KCNJ3/KCNE4 | 2 |
| BP | GO:0060349 | bone morphogenesis | 3/113 | 100/18870 | 0.0222225023589634 | 0.29604993590957 | 0.275825365593579 | BMPR1B/SCX/FGFR3 | 3 |
| BP | GO:0071772 | response to BMP | 4/113 | 179/18870 | 0.0226737281609321 | 0.29604993590957 | 0.275825365593579 | LEF1/BMPR1B/SCX/TMPRSS6 | 4 |
| BP | GO:0071773 | cellular response to BMP stimulus | 4/113 | 179/18870 | 0.0226737281609321 | 0.29604993590957 | 0.275825365593579 | LEF1/BMPR1B/SCX/TMPRSS6 | 4 |
| BP | GO:0002385 | mucosal immune response | 2/113 | 39/18870 | 0.022801825871823 | 0.29604993590957 | 0.275825365593579 | BPIFB1/GP2 | 2 |
| BP | GO:0017158 | regulation of calcium ion-dependent exocytosis | 2/113 | 39/18870 | 0.022801825871823 | 0.29604993590957 | 0.275825365593579 | SYT1/SYT9 | 2 |
| BP | GO:0043550 | regulation of lipid kinase activity | 2/113 | 39/18870 | 0.022801825871823 | 0.29604993590957 | 0.275825365593579 | EEF1A2/FGFR3 | 2 |
| BP | GO:0043620 | regulation of DNA-templated transcription in response to stress | 2/113 | 39/18870 | 0.022801825871823 | 0.29604993590957 | 0.275825365593579 | CREB3L1/MUC1 | 2 |
| BP | GO:0045746 | negative regulation of Notch signaling pathway | 2/113 | 39/18870 | 0.022801825871823 | 0.29604993590957 | 0.275825365593579 | NEURL1/DLX2 | 2 |
| BP | GO:0099622 | cardiac muscle cell membrane repolarization | 2/113 | 39/18870 | 0.022801825871823 | 0.29604993590957 | 0.275825365593579 | KCNJ3/KCNE4 | 2 |
| BP | GO:0070286 | axonemal dynein complex assembly | 2/113 | 40/18870 | 0.0239093923818909 | 0.307906321405814 | 0.286871785360813 | ZMYND10/DNAAF3 | 2 |
| BP | GO:0014046 | dopamine secretion | 2/113 | 41/18870 | 0.0250386616876611 | 0.310333269476512 | 0.289132937138537 | SYT1/SYT9 | 2 |
| BP | GO:0051954 | positive regulation of amine transport | 2/113 | 41/18870 | 0.0250386616876611 | 0.310333269476512 | 0.289132937138537 | SYT1/TRH | 2 |
| BP | GO:0072210 | metanephric nephron development | 2/113 | 41/18870 | 0.0250386616876611 | 0.310333269476512 | 0.289132937138537 | RET/PAX2 | 2 |
| BP | GO:1902742 | apoptotic process involved in development | 2/113 | 41/18870 | 0.0250386616876611 | 0.310333269476512 | 0.289132937138537 | LEF1/PAX2 | 2 |
| BP | GO:0042472 | inner ear morphogenesis | 3/113 | 105/18870 | 0.0252190089529544 | 0.310333269476512 | 0.289132937138537 | HPN/SLC44A4/PAX2 | 3 |
| BP | GO:0018108 | peptidyl-tyrosine phosphorylation | 5/113 | 276/18870 | 0.0252733533853978 | 0.310333269476512 | 0.289132937138537 | TNFRSF18/SRMS/FGFR3/IL20/DOK7 | 5 |
| BP | GO:0032963 | collagen metabolic process | 3/113 | 106/18870 | 0.0258431578607493 | 0.313990313595542 | 0.292540151289839 | CREB3L1/SCX/TMPRSS6 | 3 |
| BP | GO:0018212 | peptidyl-tyrosine modification | 5/113 | 278/18870 | 0.0259676332582172 | 0.313990313595542 | 0.292540151289839 | TNFRSF18/SRMS/FGFR3/IL20/DOK7 | 5 |
| BP | GO:0001505 | regulation of neurotransmitter levels | 4/113 | 188/18870 | 0.0265396838257753 | 0.316081647970136 | 0.29448861672922 | GRM4/RIMS4/SYT1/SYT9 | 4 |
| BP | GO:0097696 | receptor signaling pathway via STAT | 4/113 | 188/18870 | 0.0265396838257753 | 0.316081647970136 | 0.29448861672922 | TNFRSF18/RET/FGFR3/IL20 | 4 |
| BP | GO:0015837 | amine transport | 3/113 | 108/18870 | 0.0271162706711722 | 0.317369270809526 | 0.295688275966876 | SYT1/TRH/SYT9 | 3 |
| BP | GO:0002251 | organ or tissue specific immune response | 2/113 | 43/18870 | 0.0273611325070608 | 0.317369270809526 | 0.295688275966876 | BPIFB1/GP2 | 2 |
| BP | GO:0010712 | regulation of collagen metabolic process | 2/113 | 43/18870 | 0.0273611325070608 | 0.317369270809526 | 0.295688275966876 | CREB3L1/SCX | 2 |
| BP | GO:0017157 | regulation of exocytosis | 4/113 | 190/18870 | 0.0274492361748138 | 0.317369270809526 | 0.295688275966876 | RAB26/RIMS4/SYT1/SYT9 | 4 |
| BP | GO:0050804 | modulation of chemical synaptic transmission | 7/113 | 489/18870 | 0.027736129460803 | 0.31836252946313 | 0.296613680427504 | GRM4/MAPK8IP2/NEURL1/RIMS4/CACNG4/SYT1/GRIA2 | 7 |
| BP | GO:0099177 | regulation of trans-synaptic signaling | 7/113 | 490/18870 | 0.0280034163644442 | 0.318515099987408 | 0.296755828138189 | GRM4/MAPK8IP2/NEURL1/RIMS4/CACNG4/SYT1/GRIA2 | 7 |
| BP | GO:0061448 | connective tissue development | 5/113 | 285/18870 | 0.0284936117151676 | 0.318515099987408 | 0.296755828138189 | MATN3/BMPR1B/SCX/FGFR3/DLX2 | 5 |
| BP | GO:0120178 | steroid hormone biosynthetic process | 2/113 | 44/18870 | 0.0285537526503864 | 0.318515099987408 | 0.296755828138189 | CYP21A2/CACNA1H | 2 |
| BP | GO:2000300 | regulation of synaptic vesicle exocytosis | 2/113 | 44/18870 | 0.0285537526503864 | 0.318515099987408 | 0.296755828138189 | RIMS4/SYT1 | 2 |
| BP | GO:0010717 | regulation of epithelial to mesenchymal transition | 3/113 | 111/18870 | 0.0290878789298313 | 0.322204197376593 | 0.300192905849271 | LEF1/HPN/LRG1 | 3 |
| BP | GO:0048483 | autonomic nervous system development | 2/113 | 46/18870 | 0.0310003289144293 | 0.337778342452915 | 0.314703107468758 | RET/HOXB1 | 2 |
| BP | GO:0018958 | phenol-containing compound metabolic process | 3/113 | 114/18870 | 0.0311336098473015 | 0.337778342452915 | 0.314703107468758 | MAOA/HPN/DIO1 | 3 |
| BP | GO:2001243 | negative regulation of intrinsic apoptotic signaling pathway | 3/113 | 114/18870 | 0.0311336098473015 | 0.337778342452915 | 0.314703107468758 | PYCR1/CREB3L1/MUC1 | 3 |
| BP | GO:0090102 | cochlea development | 2/113 | 47/18870 | 0.0322537195255989 | 0.344123720742064 | 0.320615002977388 | HPN/PAX2 | 2 |
| BP | GO:0048562 | embryonic organ morphogenesis | 5/113 | 295/18870 | 0.0323650421423837 | 0.344123720742064 | 0.320615002977388 | HPN/SLC44A4/PAX2/DLX2/HOXB1 | 5 |
| BP | GO:0002062 | chondrocyte differentiation | 3/113 | 116/18870 | 0.0325384684864988 | 0.344123720742064 | 0.320615002977388 | BMPR1B/SCX/FGFR3 | 3 |
| BP | GO:2001233 | regulation of apoptotic signaling pathway | 6/113 | 398/18870 | 0.0325874735551197 | 0.344123720742064 | 0.320615002977388 | PYCR1/MAPK8IP2/CREB3L1/BMPR1B/MUC1/RET | 6 |
| BP | GO:0032964 | collagen biosynthetic process | 2/113 | 48/18870 | 0.03352680578277 | 0.349384607630971 | 0.325516493818861 | CREB3L1/SCX | 2 |
| BP | GO:0099601 | regulation of neurotransmitter receptor activity | 2/113 | 48/18870 | 0.03352680578277 | 0.349384607630971 | 0.325516493818861 | MAPK8IP2/CACNG4 | 2 |
| BP | GO:0010171 | body morphogenesis | 2/113 | 49/18870 | 0.0348193113997601 | 0.358141488683247 | 0.333675150939602 | LEF1/SCX | 2 |
| BP | GO:0033628 | regulation of cell adhesion mediated by integrin | 2/113 | 49/18870 | 0.0348193113997601 | 0.358141488683247 | 0.333675150939602 | MUC1/RET | 2 |
| BP | GO:0006836 | neurotransmitter transport | 4/113 | 207/18870 | 0.0359321541474902 | 0.366868236047689 | 0.341805732947142 | GRM4/RIMS4/SYT1/SYT9 | 4 |
| BP | GO:0007215 | glutamate receptor signaling pathway | 2/113 | 50/18870 | 0.0361309626410603 | 0.366868236047689 | 0.341805732947142 | GRM4/GRIA2 | 2 |
| BP | GO:0007178 | transmembrane receptor protein serine/threonine kinase signaling pathway | 6/113 | 410/18870 | 0.0368436011136592 | 0.371721427796408 | 0.346327380230306 | LEF1/BMPR1B/SCX/GDF15/TMPRSS6/LRG1 | 6 |
| BP | GO:0019083 | viral transcription | 2/113 | 51/18870 | 0.0374614883016408 | 0.373201241948422 | 0.347706101283684 | LEF1/HPN | 2 |
| BP | GO:0035036 | sperm-egg recognition | 2/113 | 51/18870 | 0.0374614883016408 | 0.373201241948422 | 0.347706101283684 | CLGN/SPACA4 | 2 |
| BP | GO:0060324 | face development | 2/113 | 52/18870 | 0.038810619686906 | 0.384225134900369 | 0.357976900006856 | LEF1/SCX | 2 |
| BP | GO:0001578 | microtubule bundle formation | 3/113 | 125/18870 | 0.0392633941444659 | 0.385709669690812 | 0.359360019209542 | NEURL1/ZMYND10/DNAAF3 | 3 |
| BP | GO:0042471 | ear morphogenesis | 3/113 | 126/18870 | 0.0400509879429497 | 0.385709669690812 | 0.359360019209542 | HPN/SLC44A4/PAX2 | 3 |
| BP | GO:0021872 | forebrain generation of neurons | 2/113 | 53/18870 | 0.0401780905927929 | 0.385709669690812 | 0.359360019209542 | LEF1/DLX2 | 2 |
| BP | GO:0086002 | cardiac muscle cell action potential involved in contraction | 2/113 | 53/18870 | 0.0401780905927929 | 0.385709669690812 | 0.359360019209542 | KCNJ3/KCNE4 | 2 |
| BP | GO:2001238 | positive regulation of extrinsic apoptotic signaling pathway | 2/113 | 53/18870 | 0.0401780905927929 | 0.385709669690812 | 0.359360019209542 | BMPR1B/RET | 2 |
| BP | GO:1903305 | regulation of regulated secretory pathway | 3/113 | 127/18870 | 0.0408465882890214 | 0.389765035239818 | 0.363138343571388 | RIMS4/SYT1/SYT9 | 3 |
| BP | GO:0086009 | membrane repolarization | 2/113 | 54/18870 | 0.041563637286017 | 0.390377964728922 | 0.363709400950258 | KCNJ3/KCNE4 | 2 |
| BP | GO:0001704 | formation of primary germ layer | 3/113 | 128/18870 | 0.041650174267164 | 0.390377964728922 | 0.363709400950258 | LEF1/SCX/PAX2 | 3 |
| BP | GO:0048706 | embryonic skeletal system development | 3/113 | 128/18870 | 0.041650174267164 | 0.390377964728922 | 0.363709400950258 | SCX/DLX2/HOXB1 | 3 |
| BP | GO:0051346 | negative regulation of hydrolase activity | 4/113 | 218/18870 | 0.0421448292323288 | 0.392690644141228 | 0.365864090301702 | CST2/CST5/SERPINA9/PAX2 | 4 |
| BP | GO:0015872 | dopamine transport | 2/113 | 55/18870 | 0.04296699848446 | 0.398010091224472 | 0.370820140813868 | SYT1/SYT9 | 2 |
| BP | GO:0030198 | extracellular matrix organization | 5/113 | 321/18870 | 0.0439169124477384 | 0.401202977890698 | 0.373794906301674 | MATN3/CREB3L1/SCX/HPN/TMPRSS6 | 5 |
| BP | GO:0050433 | regulation of catecholamine secretion | 2/113 | 56/18870 | 0.0443879153377018 | 0.401202977890698 | 0.373794906301674 | SYT1/SYT9 | 2 |
| BP | GO:0043062 | extracellular structure organization | 5/113 | 322/18870 | 0.0444048213653946 | 0.401202977890698 | 0.373794906301674 | MATN3/CREB3L1/SCX/HPN/TMPRSS6 | 5 |
| BP | GO:0045229 | external encapsulating structure organization | 5/113 | 323/18870 | 0.0448959908047734 | 0.401202977890698 | 0.373794906301674 | MATN3/CREB3L1/SCX/HPN/TMPRSS6 | 5 |
| BP | GO:0048638 | regulation of developmental growth | 5/113 | 328/18870 | 0.0474008248326562 | 0.401202977890698 | 0.373794906301674 | FGFR3/GDF15/SYT1/AGR2/GHRH | 5 |
| BP | GO:0045055 | regulated exocytosis | 4/113 | 227/18870 | 0.0476528731432128 | 0.401202977890698 | 0.373794906301674 | RAB26/RIMS4/SYT1/SYT9 | 4 |
| BP | GO:0007498 | mesoderm development | 3/113 | 136/18870 | 0.0483636322830639 | 0.401202977890698 | 0.373794906301674 | LEF1/SCX/PAX2 | 3 |
| BP | GO:0021700 | developmental maturation | 5/113 | 330/18870 | 0.0484256560390027 | 0.401202977890698 | 0.373794906301674 | NEURL1/RET/FGFR3/PAX2/SEZ6L | 5 |
| BP | GO:0010718 | positive regulation of epithelial to mesenchymal transition | 2/113 | 59/18870 | 0.0487534473926261 | 0.401202977890698 | 0.373794906301674 | LEF1/LRG1 | 2 |
| BP | GO:0031638 | zymogen activation | 2/113 | 59/18870 | 0.0487534473926261 | 0.401202977890698 | 0.373794906301674 | F12/HPN | 2 |
| BP | GO:0033619 | membrane protein proteolysis | 2/113 | 59/18870 | 0.0487534473926261 | 0.401202977890698 | 0.373794906301674 | RET/TMPRSS6 | 2 |
| CC | GO:0016324 | apical plasma membrane | 10/122 | 403/19886 | 0.000191784606245134 | 0.0341376599116339 | 0.029878022867663 | CEACAM5/SLC16A3/MUC1/ZMYND10/HPN/SLC44A4/PARD6B/KCNE4/SLC7A13/GP2 | 10 |
| CC | GO:0045177 | apical part of cell | 10/122 | 469/19886 | 0.000633279970909839 | 0.0517041629857426 | 0.0452526086451207 | CEACAM5/SLC16A3/MUC1/ZMYND10/HPN/SLC44A4/PARD6B/KCNE4/SLC7A13/GP2 | 10 |
| CC | GO:0043025 | neuronal cell body | 10/122 | 489/19886 | 0.000871418477287796 | 0.0517041629857426 | 0.0452526086451207 | MAPK8IP2/NEURL1/BMPR1B/RET/HPN/KCNH1/UNC5A/SEZ6L/GRIA2/GHRH | 10 |
| CC | GO:0034703 | cation channel complex | 6/122 | 193/19886 | 0.00123115572921041 | 0.0547864299498633 | 0.0479502757692476 | KCNJ3/KCNF1/KCNH1/CACNG1/CACNG4/CACNA1H | 6 |
| CC | GO:1990454 | L-type voltage-gated calcium channel complex | 2/122 | 12/19886 | 0.0023667082999448 | 0.0752672959435594 | 0.0658755753970833 | CACNG1/CACNG4 | 2 |
| CC | GO:0034702 | monoatomic ion channel complex | 7/122 | 300/19886 | 0.00253709986326605 | 0.0752672959435594 | 0.0658755753970833 | KCNJ3/KCNF1/KCNH1/CACNG1/CACNG4/CACNA1H/GRIA2 | 7 |
| CC | GO:0030315 | T-tubule | 3/122 | 51/19886 | 0.00378562980363737 | 0.0962631578639216 | 0.0842516106674181 | KCNJ3/CACNG1/CACNG4 | 3 |
| CC | GO:0005891 | voltage-gated calcium channel complex | 3/122 | 55/19886 | 0.00468514705918577 | 0.104244522066883 | 0.0912370743104597 | CACNG1/CACNG4/CACNA1H | 3 |
| CC | GO:0120293 | dynein axonemal particle | 2/122 | 20/19886 | 0.00659837275014539 | 0.113607182388042 | 0.0994314783762876 | ZMYND10/DNAAF3 | 2 |
| CC | GO:0060076 | excitatory synapse | 3/122 | 63/19886 | 0.00684474521418103 | 0.113607182388042 | 0.0994314783762876 | KCNJ3/SYT1/GRIA2 | 3 |
| CC | GO:0098984 | neuron to neuron synapse | 7/122 | 362/19886 | 0.00702066857454193 | 0.113607182388042 | 0.0994314783762876 | MAPK8IP2/NEURL1/KCNH1/CACNG4/SYT1/GRIA2/SYT9 | 7 |
| CC | GO:1902495 | transmembrane transporter complex | 7/122 | 394/19886 | 0.010885822718138 | 0.150713190319714 | 0.131907464029081 | KCNJ3/KCNF1/KCNH1/CACNG1/CACNG4/CACNA1H/GRIA2 | 7 |
| CC | GO:0032281 | AMPA glutamate receptor complex | 2/122 | 26/19886 | 0.0110195399074651 | 0.150713190319714 | 0.131907464029081 | CACNG4/GRIA2 | 2 |
| CC | GO:0008076 | voltage-gated potassium channel complex | 3/122 | 79/19886 | 0.0127005497460433 | 0.150713190319714 | 0.131907464029081 | KCNJ3/KCNF1/KCNH1 | 3 |
| CC | GO:0034704 | calcium channel complex | 3/122 | 79/19886 | 0.0127005497460433 | 0.150713190319714 | 0.131907464029081 | CACNG1/CACNG4/CACNA1H | 3 |
| CC | GO:1990351 | transporter complex | 7/122 | 421/19886 | 0.0151947991179353 | 0.169042140187031 | 0.147949359832528 | KCNJ3/KCNF1/KCNH1/CACNG1/CACNG4/CACNA1H/GRIA2 | 7 |
| CC | GO:0034705 | potassium channel complex | 3/122 | 90/19886 | 0.0179768133167935 | 0.186442030266054 | 0.163178122290811 | KCNJ3/KCNF1/KCNH1 | 3 |
| CC | GO:0042734 | presynaptic membrane | 4/122 | 165/19886 | 0.0188536884538707 | 0.186442030266054 | 0.163178122290811 | KCNJ3/KCNH1/RIMS4/SYT1 | 4 |
| CC | GO:0031045 | dense core granule | 2/122 | 37/19886 | 0.0216152629750679 | 0.202500884713794 | 0.177233181180613 | SYT1/SYT9 | 2 |
| CC | GO:0098839 | postsynaptic density membrane | 3/122 | 99/19886 | 0.0230722394124498 | 0.205342930770803 | 0.179720601739083 | KCNH1/CACNG4/GRIA2 | 3 |
| CC | GO:0008328 | ionotropic glutamate receptor complex | 2/122 | 41/19886 | 0.0261950185807356 | 0.221659883373015 | 0.19400155375048 | CACNG4/GRIA2 | 2 |
| CC | GO:0098686 | hippocampal mossy fiber to CA3 synapse | 2/122 | 42/19886 | 0.0273961653607097 | 0.221659883373015 | 0.19400155375048 | SYT1/SYT9 | 2 |
| CC | GO:0098878 | neurotransmitter receptor complex | 2/122 | 46/19886 | 0.0324162363362079 | 0.247398675924785 | 0.216528705126364 | CACNG4/GRIA2 | 2 |
| CC | GO:0097060 | synaptic membrane | 6/122 | 393/19886 | 0.0343002669538833 | 0.247398675924785 | 0.216528705126364 | KCNJ3/KCNH1/RIMS4/CACNG4/SYT1/GRIA2 | 6 |
| CC | GO:0030666 | endocytic vesicle membrane | 4/122 | 202/19886 | 0.0359570010136638 | 0.247398675924785 | 0.216528705126364 | CACNG4/SYT1/GRIA2/SYT9 | 4 |
| CC | GO:0034451 | centriolar satellite | 3/122 | 118/19886 | 0.0361368852474405 | 0.247398675924785 | 0.216528705126364 | ZMYND10/CCNO/PAX2 | 3 |
| CC | GO:0099634 | postsynaptic specialization membrane | 3/122 | 122/19886 | 0.0392819157894296 | 0.258969667056239 | 0.226655888375656 | KCNH1/CACNG4/GRIA2 | 3 |
| CC | GO:0030672 | synaptic vesicle membrane | 3/122 | 126/19886 | 0.0425617337642981 | 0.261240986553278 | 0.228643796628535 | RAB26/SYT1/SYT9 | 3 |
| CC | GO:0099501 | exocytic vesicle membrane | 3/122 | 126/19886 | 0.0425617337642981 | 0.261240986553278 | 0.228643796628535 | RAB26/SYT1/SYT9 | 3 |
| CC | GO:0014069 | postsynaptic density | 5/122 | 315/19886 | 0.044852877968297 | 0.266127075945229 | 0.232920208396771 | MAPK8IP2/NEURL1/KCNH1/CACNG4/GRIA2 | 5 |
| MF | GO:0022843 | voltage-gated monoatomic cation channel activity | 8/111 | 153/18496 | 3.92159500968755e-06 | 0.0009764771574122 | 0.000883390875866459 | KCNJ3/KCNF1/KCNH1/KCNK15/CACNG1/KCNE4/CACNG4/CACNA1H | 8 |
| MF | GO:0005244 | voltage-gated monoatomic ion channel activity | 8/111 | 200/18496 | 2.76946940763855e-05 | 0.00229865960833999 | 0.00207953141485842 | KCNJ3/KCNF1/KCNH1/KCNK15/CACNG1/KCNE4/CACNG4/CACNA1H | 8 |
| MF | GO:0022832 | voltage-gated channel activity | 8/111 | 200/18496 | 2.76946940763855e-05 | 0.00229865960833999 | 0.00207953141485842 | KCNJ3/KCNF1/KCNH1/KCNK15/CACNG1/KCNE4/CACNG4/CACNA1H | 8 |
| MF | GO:0004869 | cysteine-type endopeptidase inhibitor activity | 4/111 | 37/18496 | 6.96467338055721e-05 | 0.00433550917939686 | 0.00392221079852432 | CST2/CST5/CST9/CST9L | 4 |
| MF | GO:0022839 | monoatomic ion gated channel activity | 9/111 | 325/18496 | 0.000150194356010214 | 0.00637829508661315 | 0.00577026061524081 | KCNJ3/KCNF1/KCNH1/KCNK15/CACNG1/KCNE4/CACNG4/CACNA1H/GRIA2 | 9 |
| MF | GO:0022836 | gated channel activity | 9/111 | 326/18496 | 0.000153693857508751 | 0.00637829508661315 | 0.00577026061524081 | KCNJ3/KCNF1/KCNH1/KCNK15/CACNG1/KCNE4/CACNG4/CACNA1H/GRIA2 | 9 |
| MF | GO:0005249 | voltage-gated potassium channel activity | 5/111 | 99/18496 | 0.000324810124326868 | 0.01155396013677 | 0.0104525363317218 | KCNJ3/KCNF1/KCNH1/KCNK15/KCNE4 | 5 |
| MF | GO:0004866 | endopeptidase inhibitor activity | 6/111 | 168/18496 | 0.000529485005128499 | 0.0163431326914464 | 0.0147851633733652 | CST2/CST5/SPINK4/SERPINA9/CST9/CST9L | 6 |
| MF | GO:0004252 | serine-type endopeptidase activity | 6/111 | 174/18496 | 0.000636913735249968 | 0.0163431326914464 | 0.0147851633733652 | F12/CFB/HPN/F7/TMPRSS6/TPSG1 | 6 |
| MF | GO:0030414 | peptidase inhibitor activity | 6/111 | 175/18496 | 0.000656350710499855 | 0.0163431326914464 | 0.0147851633733652 | CST2/CST5/SPINK4/SERPINA9/CST9/CST9L | 6 |
| MF | GO:0005267 | potassium channel activity | 5/111 | 122/18496 | 0.000844163281614232 | 0.0172052289232258 | 0.0155650771066173 | KCNJ3/KCNF1/KCNH1/KCNK15/KCNE4 | 5 |
| MF | GO:0061135 | endopeptidase regulator activity | 6/111 | 184/18496 | 0.000852740238749002 | 0.0172052289232258 | 0.0155650771066173 | CST2/CST5/SPINK4/SERPINA9/CST9/CST9L | 6 |
| MF | GO:0005261 | monoatomic cation channel activity | 8/111 | 333/18496 | 0.000898264963863196 | 0.0172052289232258 | 0.0155650771066173 | KCNJ3/KCNF1/KCNH1/KCNK15/CACNG1/KCNE4/CACNG4/CACNA1H | 8 |
| MF | GO:0008236 | serine-type peptidase activity | 6/111 | 192/18496 | 0.00106284844572463 | 0.0189035187846737 | 0.0171014712319601 | F12/CFB/HPN/F7/TMPRSS6/TPSG1 | 6 |
| MF | GO:0017171 | serine hydrolase activity | 6/111 | 196/18496 | 0.00118177536652028 | 0.0196174710842367 | 0.0177473633989713 | F12/CFB/HPN/F7/TMPRSS6/TPSG1 | 6 |
| MF | GO:0005216 | monoatomic ion channel activity | 9/111 | 443/18496 | 0.00140063981143958 | 0.0217974570655285 | 0.0197195341873731 | KCNJ3/KCNF1/KCNH1/KCNK15/CACNG1/KCNE4/CACNG4/CACNA1H/GRIA2 | 9 |
| MF | GO:0002162 | dystroglycan binding | 2/111 | 11/18496 | 0.00189489894951273 | 0.0277546963781571 | 0.0251088777210975 | AGR2/AGR3 | 2 |
| MF | GO:1902282 | voltage-gated potassium channel activity involved in ventricular cardiac muscle cell action potential repolarization | 2/111 | 12/18496 | 0.00226498075873219 | 0.0313322338291286 | 0.028345373237935 | KCNJ3/KCNE4 | 2 |
| MF | GO:0061134 | peptidase regulator activity | 6/111 | 228/18496 | 0.00253794708498315 | 0.0320345852517856 | 0.028980770424359 | CST2/CST5/SPINK4/SERPINA9/CST9/CST9L | 6 |
| MF | GO:0015079 | potassium ion transmembrane transporter activity | 5/111 | 157/18496 | 0.00257305905636832 | 0.0320345852517856 | 0.028980770424359 | KCNJ3/KCNF1/KCNH1/KCNK15/KCNE4 | 5 |
| MF | GO:0086008 | voltage-gated potassium channel activity involved in cardiac muscle cell action potential repolarization | 2/111 | 15/18496 | 0.00356128087971083 | 0.0422266161451427 | 0.0382012084339908 | KCNJ3/KCNE4 | 2 |
| MF | GO:0005245 | voltage-gated calcium channel activity | 3/111 | 52/18496 | 0.00375432430302052 | 0.042492125066005 | 0.0384414067390618 | CACNG1/CACNG4/CACNA1H | 3 |
| MF | GO:0005544 | calcium-dependent phospholipid binding | 3/111 | 53/18496 | 0.00396237377097593 | 0.0428970029988264 | 0.0388076881917094 | CPNE7/SYT1/SYT9 | 3 |
| MF | GO:0046873 | metal ion transmembrane transporter activity | 8/111 | 441/18496 | 0.00510456651654615 | 0.0529598776091663 | 0.0479112822167051 | KCNJ3/KCNF1/KCNH1/KCNK15/CACNG1/KCNE4/CACNG4/CACNA1H | 8 |
| MF | GO:0030276 | clathrin binding | 3/111 | 70/18496 | 0.00860286621347159 | 0.085684547486177 | 0.0775163524077019 | EPN3/SYT1/SYT9 | 3 |
| MF | GO:0004175 | endopeptidase activity | 7/111 | 389/18496 | 0.00903780592737598 | 0.0865543721506392 | 0.0783032578323263 | F12/CFB/CAPN9/HPN/F7/TMPRSS6/TPSG1 | 7 |
| MF | GO:0008066 | glutamate receptor activity | 2/111 | 27/18496 | 0.011360052028383 | 0.104634809987172 | 0.0946601113390605 | GRM4/GRIA2 | 2 |
| MF | GO:0019199 | transmembrane receptor protein kinase activity | 3/111 | 79/18496 | 0.0119553456347919 | 0.104634809987172 | 0.0946601113390605 | BMPR1B/RET/FGFR3 | 3 |
| MF | GO:0005251 | delayed rectifier potassium channel activity | 2/111 | 28/18496 | 0.0121863834924819 | 0.104634809987172 | 0.0946601113390605 | KCNH1/KCNE4 | 2 |
| MF | GO:1902936 | phosphatidylinositol bisphosphate binding | 3/111 | 108/18496 | 0.0272585401914163 | 0.226245883588755 | 0.204678161437301 | KCNH1/SYT1/SYT9 | 3 |
| MF | GO:0004857 | enzyme inhibitor activity | 6/111 | 386/18496 | 0.0289005076425303 | 0.232136335580324 | 0.210007084397334 | CST2/CST5/SPINK4/SERPINA9/CST9/CST9L | 6 |
| MF | GO:0005246 | calcium channel regulator activity | 2/111 | 45/18496 | 0.0298818751883755 | 0.232518341309547 | 0.210352674023433 | CACNG1/CACNG4 | 2 |
| MF | GO:0005262 | calcium channel activity | 3/111 | 128/18496 | 0.0418626331546303 | 0.285414992181724 | 0.258206756824726 | CACNG1/CACNG4/CACNA1H | 3 |
| MF | GO:0099106 | ion channel regulator activity | 3/111 | 134/18496 | 0.0468740111477191 | 0.285414992181724 | 0.258206756824726 | CACNG1/KCNE4/CACNG4 | 3 |
| MF | GO:1901682 | sulfur compound transmembrane transporter activity | 2/111 | 58/18496 | 0.0474596508478026 | 0.285414992181724 | 0.258206756824726 | SLC44A4/SLC16A6 | 2 |
| ONTOLOGY | ID | Description | GeneRatio | BgRatio | pvalue | p.adjust | qvalue | geneID | Count |
| BP | GO:0042391 | regulation of membrane potential | 10/113 | 440/18870 | 0.000313605341889873 | 0.238205619326982 | 0.221932667661103 | PYCR1/MAPK8IP2/HSH2D/KCNJ3/KCNH1/KCNK15/RIMS4/KCNE4/CACNA1H/GRIA2 | 10 |
