# Supplementary material for: Identification of NETs and inflammation-related prognostic genes in breast cancer and PCR experimental validation
Source: Front Genet. 2026 Aug 21;17:1900298. doi: 10.3389/fgene.2026.1900298 (PMC13541293; doi:10.3389/fgene.2026.1900298)
Supplement: Supplementary file 2 [file DataSheet2.zip › Supplementary Table0713/supplementary table 6.docx]

gene

SEMA3F

LASP1

ICA1

SLC7A2

ZMYND10

CROT

PDK2

ABCC8

ZNF263

SPATA20

ARSD

PNPLA4

CACNA2D2

BAIAP3

RHOBTB2

POMT2

GIPR

PHF7

NISCH

MKS1

SEMA3B

ELOVL5

CALCOCO1

MAN2B2

GPRC5A

GLT8D1

ZNF582

SERPINB1

ZNF839

RABEP1

IFT88

ALG1

FUT8

DNAH5

PARP3

RETSAT

JADE2

GEMIN8

FAM214A

FAM184B

LETMD1

SLC4A8

PTGER3

HHAT

POLR3E

YIPF1

GATB

PIGV

MED29

SPA17

ERBB3

REEP1

MAST4

SIDT1

CA12

NTN4

CELSR1

RGS11

SLC46A1

DGKD

RAPGEF3

IGSF9B

LRP2

KIAA0141

PGR

ZNF671

KIAA1467

TAF9

TTC39A

ACOX3

SULT2B1

C3orf18

KIF9

CFAP61

IGBP1

ANKRD24

SLC9A1

MUL1

CHRD

RAB11FIP3

CERS4

ESR1

SLC7A8

EFHC1

TSPAN15

CIRBP

IGFALS

MISP

PALM

MKNK2

CARD10

PATZ1

CBX7

CGRRF1

PIGH

GSTZ1

SAMD15

ITPK1

C14orf93

DCAF11

GMPR2

RIMS4

SPEF1

MROH8

CST3

APMAP

CACNA1F

SLC25A14

ITIH6

MAGED2

SYTL4

CAB39L

ZNF629

LYRM1

TSC2

ZNF500

METRN

RHBDL1

ZNF174

CLUAP1

TOX3

KAT8

SYT17

TMC5

IKBKB

ASAH1

VRK3

TJP3

NAPA

MEIS3

TMEM205

HPN

SCN1B

CFAP69

IQCE

CHN2

TSPAN13

AGR2

URGCP

SPATA6L

PTGR1

TBC1D13

ABCA2

GATA3

DNMBP

OBFC1

SORCS1

DNAJC12

CAMTA2

RAB5C

SLC16A6

TBC1D9

INPP4B

WFS1

GLRB

SIAE

CCND1

TRIM3

SLC22A18

COQ5

LIN7A

KRT18

TNS2

SCNN1A

COPZ1

TBC1D30

PRKAB1

PEX7

ENPP5

NUDT12

NME5

MRPS30

IK

THG1L

SKP1

TXNDC15

ERGIC1

STC2

TUSC2

NPRL2

CYB561D2

C3orf52

EFCC1

NEK11

HEMK1

CISH

ABHD14B

VIPR1

LMAN2L

REEP6

PCSK4

DOK1

IGFBP2

PLCD4

MLPH

TPO

SLC1A4

SPR

DHCR24

MORN1

RALGPS2

KIAA1324

TRIM62

MEF2D

OSCP1

ECE1

HMGCL

TSPAN1

SLC19A2

FAAH

SYF2

MAN1C1

IFT46

MREG

CASC1

USP35

MYB

CCDC176

DNAL1

IRF2BPL

ACOT2

COQ6

PPP1R3C

CCDC170

MRPS14

MTERF2

CRY2

LIAS

RAMP3

ATP7B

PREX1

SDC4

PARD6B

BCAS4

KCNK15

IQSEC2

SPDEF

RAB17

SLC25A35

SRMS

PSD4

PSPN

EML2

MAX

NECAB3

GRPR

MCF2L

PRRG2

WNK4

FGD3

PPCS

COX7C

KDM4B

IFT22

IVD

ANAPC13

LRRC6

FOXA1

EAPP

REEP5

GAMT

ERMARD

NXNL2

PVRL2

SLC27A1

NDUFA10

PGPEP1

TRPM4

MED18

CASZ1

POLN

TTLL9

SH3BGRL

RAMP2

NINJ1

RARA

FMO5

RAI2

MCCC2

MATN3

HSD17B7

ARFIP2

TMEM128

DLG4

RGS22

MTL5

RSG1

CASP9

TCEAL4

TMEM254

SARAF

C14orf159

BHLHE40

VAV3

TRIM45

IL6ST

RERG

DDB2

ELP2

C5AR2

TBX3

P2RX4

ATP5G2

GLS2

TSPAN31

ZC3H10

GLUL

ISCU

CHAD

CCDC115

KIF12

C9orf156

XPA

LMX1B

TMEM8B

ARRB1

ANKRD42

NUMA1

SYTL2

RAB30

RMDN3

TMEM62

DYNC2LI1

ARL3

MDH1B

APH1B

SUOX

CCDC65

CERS5

MAP3K12

TMBIM6

TTC6

STON2

EFCAB11

PTGR2

AK7

C14orf79

CDAN1

TLE3

IGF1R

BBS4

SCAMP2

WDR93

LRRC46

ARSG

G6PC3

SLC39A6

CCDC40

FAM134C

IGFBP4

CTB-55O6.8

NFIC

CIB3

SIRT3

LMTK3

MMEL1

KIAA0319L

CELSR2

FAM63A

ANXA9

SELENBP1

CREB3L4

SCCPDH

SOX13

PLEKHA6

RHOB

SFXN5

NPHP1

AFF3

PHOSPHO2

SUMF1

ANKMY1

FAM134A

SLC25A38

LRIG1

NICN1

ILDR1

SLC26A1

BTF3

PCYOX1L

RMND5B

BOD1

ZMAT2

DCDC2

TRIM41

TPBG

SYTL5

SYBU

UGCG

ALAD

ASTN2

ST8SIA6

ANKRD30A

LRRC27

TTC12

TMEM25

TM7SF2

TMEM218

PDCD4

SPATA4

FSIP1

PRSS23

PIH1D2

FBXO4

GFRA1

CCDC74B

SPEF2

GPD1L

WDR78

HHEX

STK32B

CLGN

CETN3

FBXO36

DNAAF1

C16orf74

FAM134B

CCDC173

NCAM2

ZNF18

PPM1J

LRGUK

KDM8

MED7

TMBIM4

CFAP70

FAM161B

CDK20

SFXN2

PCDH1

SUSD3

CACNA1D

ZNF19

TSC22D3

LCA5L

C12orf43

CLSTN2

RIBC1

PINK1

CCDC24

C2orf81

CYB5R1

BTG2

AMFR

TPPP3

ZFYVE28

IQCC

ABCG1

TFF3

TFF1

RSPH1

C9orf116

C9orf117

ZER1

SPTBN4

LRRC56

ZNF385A

ASB16

TNFSF13

ZG16B

ADCY9

AKR7A3

SYNC

KIAA1522

UBXN10

KLHDC9

DENND2D

SPATA17

CAPN13

CFAP36

CCDC74A

NOSTRIN

BBS5

STK36

NEK10

GLB1L

NUAK2

KIAA1407

SMIM14

PRRT3

GRIK3

DNALI1

IFT122

PRKCD

APEH

FAM198B

MOCS2

CMBL

C5orf63

TIGD6

CMYA5

ANKRA2

NSA2

CCDC127

GALNT10

CAMLG

IQUB

ADCY1

LETM2

GKAP1

FBP1

TTC8

TMEM63C

FAAH2

AK8

VWA2

SALL2

FRAT1

AGBL2

MOAP1

ADAMTS15

C16orf71

WBP1L

C10orf32

CYB5A

IQCD

C16orf45

PEX11A

STAT6

MBD6

FAM102A

DOLPP1

COQ7

PRRT2

ZNF646

SERTAD3

TMC4

DNAAF3

CYB5D2

SPRYD3

HID1

GHDC

RAB26

ABCA3

VPS37C

BSCL2

ULK4

KIF5C

PDHB

KCTD6

DEGS2

COG7

HEXIM2

ING2

DYNLRB2

AXIN2

NPNT

TCTN2

PPIP5K1

ABHD15

LCMT2

STX18

CPLX1

MFSD7

IRS1

AR

FAM110B

NSMCE1

TAPT1

BTD

REPS2

ZNF764

ZNF747

MYRIP

ALCAM

FRMPD2

KRT8

SPATA24

LONRF2

SYT9

GPR27

NUDT6

DCDC1

MORN4

RBKS

TRIM8

KCND3

NAT1

BCL2L1

CXXC5

BCL2

CFAP46

ZNF540

ATPAF2

LRRC48

LAMB2

AZU1

STARD5

CFAP53

TTC36

TCEAL1

EFCAB12

SSH3

DCAKD

TADA2B

CCDC96

ABLIM3

AGR3

GPR160

FAM174A

ADCY6

ZBTB4

TNK1

GTF2IRD2B

C12orf76

ZNF497

C4orf32

PCP2

TP53I11

PHYHD1

TMEM9B

SCUBE2

PAAF1

TOM1L2

ORAI3

SLC35A4

SPRYD4

WDR25

PRR15

CNTD1

ZNF843

RUFY1

MTHFR

ACOT4

BET1L

DALRD3

WDR6

DNAJC22

P4HTM

DTX3

SLC25A20

ERBB4

MPI

ZNF552

ZBTB7A

ERICH3

CTC1

MRFAP1L1

MRFAP1

TMEM107

FUCA1

ZBTB42

GLIPR1L2

ANKRD30B

RPH3AL

ZNF467

RNF135

HKR1

C5orf30

SIAH2

TMEM30B

KBTBD3

NXPH3

CCDC87

ABAT

GDPGP1

PRR36

EPHA10

CCDC125

CCDC159

FBXL7

ZNF703

MAATS1

C1orf64

COA3

LRTOMT

LRRC26

FAM110C

SEMA4B

INPP5J

RBM11

FAM174B

ADAMTSL5

RTN4RL1

RXRA

CCDC30

ZNF396

BCDIN3D

TPCN1

HEXIM1

MAPT

SERPINA11

TMEM232

EFCAB6

TMPRSS6

LYPD6

BCAM

EPOR

MAGI2

KCNJ11

IFT140

C17orf97

THSD4

C1orf168

TPRG1

COL4A5

ENO4

JAKMIP3

C19orf54

DUSP28

ZNF563

FAM120AOS

C9orf152

KCTD21

FAM47E

PTPRT

ACADSB

GREB1

EVL

PPP1R26

C20orf96

SULT1A1

TCEAL3

KIAA1211L

HCAR1

TMEM26

ANXA6

ZNF763

ZNF785

NEK5

CYSRT1

SLC22A4

LRP10

SLC22A5

SLC2A10

ENPP1

CXorf40A

CFAP43

KLHDC1

MYO1C

KIF13B

ZNF311

ELOVL2

TMEM229B

SLC29A3

NUDT16

ECI2

RUNDC1

ZFP2

EFCAB2

CAPN8

FANK1

SYS1

TCEA3

HSD17B8

SLC44A4

HSPA1L

ZNF814

DDR1

TTC25

TCTN1

ATP6AP1L

RGL3

TTC23L

SLC48A1

CRIP1

EMP2

AP1G2

SH3D21

ANG

PLIN5

STARD10

VAMP2

RAB6C

CT62

SCAMP4

PROB1

LYRM9

KIAA0040

ARHGEF38

KLHL41

C1orf226

EIF4EBP3

ZNF487

CFAP57

TMEM199

DBNDD2

C4A

CCDC153

SYS1-DBNDD2

EID1

FDXACB1

DYX1C1

NHLRC4

FNTB

THTPA

CTB-50L17.14

SMIM22

ZNHIT3

C18orf65

DACH1

SRCIN1

FGR

LAP3

TMEM176A

CASP10

CFLAR

CD38

ITGAL

MAP3K14

CEACAM21

MATK

CD79B

SELE

IL32

TRAF3IP3

ETV7

CD4

BTK

FYN

ZBTB32

TYROBP

ALOX5

CD6

WAS

CD74

RUNX3

GRAMD1B

BIRC3

DEF6

PLEKHO1

NR1H3

TYMP

SLAMF7

BTN3A1

SH2D2A

TNFRSF1B

POU2F2

STAP1

MSR1

ADAM28

LCP2

ARAP2

TNFRSF17

CELF2

UTS2

TNFRSF9

FOXP3

TNIP3

HERPUD1

RELT

TRAF1

PRDM1

TBXAS1

GNA15

LTK

TSPAN32

DGKA

ANKRD44

PRKCQ

CD84

SPI1

SP100

RASGRP2

KCNAB2

CLEC2D

RAB27A

DAPP1

SLC12A3

FCGR2B

STK10

ACAP1

DHRS9

ST6GAL1

TBX21

IPCEF1

TXK

ARHGAP15

PAG1

ICAM3

NFKB2

APBB1IP

CST7

LAMP3

P2RY10

SLC1A3

SP140

TCF7

PTPRC

STK17B

FYB

P2RX5

ITGAE

SMAP2

FCN1

PILRA

AKR1B1

NME8

LAT2

ACHE

FTL

CETP

CASS4

REM1

SIGLEC1

SIRPG

P2RX7

GMIP

LAG3

ARHGAP4

RGS1

ICAM1

LYZ

FLT3LG

CD209

NLRC4

SEL1L3

NLRP1

CEBPE

DAZL

UNC13D

CECR1

IL12RB1

MYO9B

DERL3

OSM

CYTH4

MFNG

LGALS2

SH3BP1

HMOX1

APOBEC3H

APOL4

APOL1

GRAP2

NCF4

CSF2RB

IL2RB

GZMH

GZMB

RIN3

LGMN

ASB2

TCL1A

PSME2

PLTP

MMP9

CD40

SLA2

NFATC2

CTSZ

RASSF2

SIRPB1

HCK

TLDC2

SAMHD1

ASIP

PPP1R16B

TLR8

RENBP

PIM2

CD40LG

KIAA0226L

TNFSF13B

IRG1

CORO1A

CCL22

CCL17

COTL1

MEFV

IL21R

CSK

CTSH

IL7

MAN2B1

MAP4K1

RELB

CD37

FCER2

IL4I1

LILRB1

LILRA1

RASAL3

EBI3

SHD

DENND3

CEACAM4

CD79A

NKG7

CD33

SIGLEC5

LILRB5

JAK3

HAMP

PBX4

PIK3CG

TFEC

CPVL

CCL24

GIMAP2

TMEM176B

AKNA

TNFSF8

DOCK8

PTGDS

RASSF4

C10orf54

SPOCK2

P2RX1

ICAM2

CYTH1

CCL2

CCL8

ABI3

FAM20A

IL2

CLNK

CTSC

CRTAM

LPXN

MS4A6A

MS4A4A

IL10RA

SLC15A3

CD5

C11orf21

POU2AF1

CD69

CLEC2B

SELPLG

BIN2

IL23A

IL26

IFNG

PTPN6

CLEC4A

AICDA

KLRB1

BTN3A3

FAM65B

CD83

TREML2

VNN1

VNN2

TRIM38

TFEB

LY86

GZMK

ITK

IL12B

ST8SIA4

CDX1

CD86

ZAP70

CYTIP

ITGA4

IFIH1

STAT1

GNLY

IL18R1

IL18RAP

WIPF1

PLEK

RNF19B

NCF2

AMPD1

CD2

KIF21B

SLAMF1

CD48

PLA2G2D

GBP1

CR2

FASLG

HSD11B1

C1orf54

LRMP

TNFAIP3

NPC2

FLVCR2

CD274

CXorf21

GPR31

KCNJ5

PTK2B

TNFRSF8

ADCY7

CSTA

CD80

CCRL2

CCR2

TMEM156

CXCR4

SASH3

LAX1

CD244

LY9

SRGN

HVCN1

ARHGAP9

NCKAP1L

NMI

BATF3

TOX2

GTSF1L

ZNF831

ZBP1

IL9R

BTN2A2

CPNE5

GPR18

IRF1

SEPT6

HS3ST3B1

IL1B

TNFSF9

CD70

C3

TNFSF14

SIRPD

S1PR4

IGFLR1

HCST

CCR7

TSKS

STAT5A

CFP

EVI2A

FAM78A

BCL11B

FGL2

ASPHD2

VPREB3

APOL3

IGLL1

APOL2

RAC2

APOBEC3A

IRF5

WDFY4

CD68

SIGLEC9

RNASE1

APOC1

FCHO1

KLHDC7B

LSP1

GMFG

THEMIS2

ANGPTL6

C19orf66

MPP1

LILRB2

CCL25

IDO1

RFTN1

MGAT1

TRIM21

FCRLA

EMILIN2

TRIM22

CLEC10A

XAF1

FCRL2

ACY3

ALOX5AP

CHIT1

EPSTI1

EPHB2

PRAM1

GIMAP6

GIMAP4

ADAMDEC1

CD180

IRAK2

PTPN22

CD101

AP4B1

FKBP11

IL2RA

IL15RA

DOCK2

KLRD1

KLRC1

RAB33A

CTSL

ADAM19

HAVCR2

TESPA1

AGAP2

MICAL1

STX11

NPL

GPR55

SP110

DOCK10

WNT10A

CYP27A1

DRAM1

LCP1

AOAH

MYO1G

LIMD2

RTP4

BLK

IL10

BIN1

SIT1

CD72

IRF4

TLR2

SLCO2B1

IL18BP

FXYD2

CASP1

CASP5

PLCB2

IFI44

STAMBPL1

STAT4

SEMA7A

HERC5

IL21

CXCL9

PARVG

KLRG1

TAPBPL

CD27

GPR84

ITGB7

RHOF

GPR65

SLC24A4

WARS

PSTPIP1

BCL2A1

PML

ITGAX

IGSF6

NLRC5

IRF8

ARRB2

ZMYND15

PIK3R5

SECTM1

VAV1

TRPM2

EMP3

MYO1F

SIGLEC10

SLC2A5

SH3BGRL3

MOB3C

C1orf162

CD53

GPA33

XCL1

XCL2

FCGR2A

FCRL5

PTPN7

GALM

GRIP2

PLA1A

PLAC8

1-Mar

GZMA

TNFAIP8

ARHGAP26

TIMD4

PLA2G7

DOK3

PPP1R18

FGD2

NFKBIE

SCML4

RSPO3

SLC22A3

TMEM140

GPR174

IL2RG

ATP6V1B2

DOK2

GBGT1

CDHR1

FUOM

RGS10

FERMT3

KLRF1

FCGR1A

CD226

RGS18

IL18

PIP4K2A

PTPRO

ADAM8

FLI1

GLT1D1

CYSLTR2

PDE3B

CAMK4

RASGRP3

PRDM8

JAKMIP1

BANK1

MERTK

CD96

CD8A

MCOLN2

GRAP

GPR15

GBP5

LY96

PLCL2

SAMSN1

SLC7A7

PIK3AP1

VSIG4

ELMO1

SLA

CLIC2

CXCL13

PPP2R2B

MS4A1

ITGAD

GHRL

NECAP2

DUSP2

CD1D

CD1C

CD1B

CD1E

HMHB1

NCF1

SLAMF8

RNF166

FCER1G

C1QC

ARHGAP27

C1R

RGL4

RLTPR

TNFRSF13C

UBASH3A

GAB3

ITGB2

SLC2A6

AMICA1

CD3G

CXCR5

IL6R

CCR5

FCRL3

HK3

IKZF3

TREML1

SCIMP

ASGR2

CCDC42

CYB561A3

LAPTM5

GBP2

GBP4

GFI1

VCAM1

NLRP3

SLAMF6

OXER1

HAAO

FCAMR

CTSS

TNFAIP8L2

ARHGAP25

KBTBD8

ARPC2

EOMES

FCRL4

TRAT1

FCRL1

MNDA

PYHIN1

IFI16

AIM2

CTLA4

ICOS

CD200R1

CCR1

IL15

ANKRD33B

CSF2

SAMD3

STK17A

TAGAP

CTSB

SYK

CYBB

FOLR2

IFI27

C2

PLEKHF1

PRKCB

CLEC4E

CLEC4D

TVP23A

B2M

SLFN5

MEI1

GNGT2

SNX20

DPEP2

CD3D

GNG8

JSRP1

FAM129C

LAIR1

LAIR2

KIR3DL1

TMIGD2

SLC43A2

CD300C

CD300A

TMC8

GSDMA

NLRC3

BATF2

CCDC88B

PNOC

PTGDR

XIRP1

TAP1

MLKL

IL7R

VAMP5

INPP5D

LGALS9

SIGLEC7

CXCL10

CXCL11

RNASE2

PTAFR

RNASE6

CD52

HTRA4

PLEKHA2

GPR183

C15orf27

ITGAM

GPR25

ZNF804A

CD14

MZB1

SERPINB9

STAT2

GTSF1

OSCAR

CEACAM3

FPR2

FPR1

GIMAP8

CHST11

PTGER4

PIK3CD

PTCRA

P2RY6

RGS19

TMEM51

C3AR1

MAL

MOB3A

CD8B

CCL11

ISG20

CXCR6

CLEC7A

CLEC12A

IL16

CTSW

KLHL6

THEMIS

SLFN11

CCL19

MYD88

HPSE

PARP14

PARP15

ABCD2

C1QB

C1QA

OLR1

EHBP1L1

NABP1

XCR1

CD7

NRROS

TLR10

TLR1

ZNF80

CMKLR1

P2RY14

GPR171

CATSPER1

TBC1D10C

LRRC25

GAPT

ZNF683

HSF5

RHOG

KCNA3

SAMD9L

CD19

ZBED2

CD163

ANXA2R

KCNJ10

GPR150

ZC3H12D

CD28

CD300LB

GIMAP7

HLA-DQB1

CIITA

LACC1

GPBAR1

CCR8

TMEM150B

SEPT1

HCLS1

HMHA1

FUT7

PRF1

FCRL6

OR52N4

CCL13

P2RY13

SLC9A9

TIGIT

P2RY8

FAM159A

SPNS3

CSF1R

LCK

SLC8A1

PMCH

GPR132

MX2

KLRC4

IZUMO1R

CCR4

SH2D1A

ADAP2

CLECL1

CSF1

LPAR5

FMNL1

MIXL1

GP1BA

SOCS1

TCN2

STAC3

PDE6G

SNAI3

IKZF1

EVI2B

TRIM69

FFAR3

C16orf54

CD300LF

FFAR4

BTLA

CD300E

BTN3A2

ARHGAP30

CXCR3

LILRB4

TNFRSF4

TRABD2A

LILRA5

FNBP1

FPR3

TRPV2

SEMA4D

CARD9

SOWAHD

TTC24

CLEC17A

RTP5

RAB42

RUFY4

C19orf35

PLSCR1

PDCD1

SELL

CERKL

IDO2

FAM26F

CNR2

KIR2DL4

NUGGC

FAM179A

NCR1

PAX5

HLA-DRB1

S100A4

SIRPB2

GIMAP5

TLR7

HLA-DQA1

GM2A

ADA

CASP4

DTHD1

GAL3ST4

ACSL5

IL27

C5AR1

SPN

GZMM

MPEG1

PDCD1LG2

CR1L

PSAP

MDS2

CLEC9A

FCGR1B

TOR4A

CLEC4C

CSF2RA

CARD11

HLA-DRB5

SH2D1B

CCDC69

EGFL6

RCSD1

GRIN3A

CD247

SUCNR1

TOX

CD3E

ITPRIPL1

TGM2

CR1

FCGR3A

C10orf128

CXorf65

HLA-DOA

HLA-DMA

PSMB8

TAP2

HLA-DRA

CARD16

AIF1

NCR3

LST1

HLA-C

LILRB3

HLA-E

HLA-G

HLA-F

SLFN12L

PSMB10

TMSB4X

DENND1C

ARRDC5

CLEC6A

HLA-A

GIMAP1

GPSM3

KLRK1

UBD

C19orf38

LCNL1

C10orf105

IFI30

CKLF

FXYD7

APOL6

HLA-DPB1

LTA

LTB

PATL2

HLA-DPA1

HLA-DQB2

TNF

HLA-B

NFAM1

SLFN14

HLA-DQA2

APOBEC3G

LILRA4

PSMB9

KIR3DL2

TNFRSF13B

HLA-DOB

PLEKHO2

HLA-DMB

IL10RB

KIR2DL3

APOBEC3D

LILRA6

APOBEC3C

INSL3

XXbac-BPG181M17.5

SIGLEC14

SIGLEC12

IGLL5

CARD17

KLRC4-KLRK1

TIFAB

GH1

RP11-812E19.9

MRC1

CTC-479C5.12

C19orf84

RASSF5

SPIB

CCL5

MILR1

CCL23

CCL4

CCL18

PIK3R6

CCL3

SSTR3
