# Supplementary material for: Identification of NETs and inflammation-related prognostic genes in breast cancer and PCR experimental validation
Source: Front Genet. 2026 Aug 21;17:1900298. doi: 10.3389/fgene.2026.1900298 (PMC13541293; doi:10.3389/fgene.2026.1900298)
Supplement: Supplementary file 2 [file DataSheet2.zip › Supplementary Table0713/supplementary table 10.docx]

| id | OS | OS.time | ZMYND10 | IL12B | CXCL13 | TFF1 | LTB | SPIB | riskScore_out | risk_out |
| --- | --- | --- | --- | --- | --- | --- | --- | --- | --- | --- |
| GSM519117 | 0 | 7 | 5.78177565170352 | 5.92174672426215 | 13.4278226580247 | 11.9016366545372 | 11.8986519844589 | 8.96694900758395 | -3.073305452 | Low |
| GSM519118 | 0 | 7.4 | 6.29247612439692 | 4.42741326765665 | 9.84116800478124 | 9.85371050509239 | 9.26523117753068 | 6.65841098214898 | -2.508697504 | High |
| GSM519119 | 0 | 7.2 | 5.73503066919634 | 7.48374377225525 | 11.2433190658149 | 13.3604574682515 | 10.6962031556592 | 7.2065611808233 | -2.806309602 | Low |
| GSM519120 | 0 | 6.3 | 6.49390660276963 | 6.47142537705471 | 10.9890494246196 | 8.78839138973447 | 9.17342268454352 | 5.58672049362594 | -2.430339331 | High |
| GSM519121 | 0 | 7 | 4.78190677744618 | 5.90257504047295 | 11.1110799148278 | 10.0016563807409 | 9.78780413751072 | 7.71547576743948 | -2.618575377 | Low |
| GSM519122 | 0 | 7.3 | 6.95454990570379 | 6.76008936513249 | 11.1970480292577 | 7.16006147839331 | 9.21370592586164 | 6.69991526279591 | -2.597654579 | Low |
| GSM519123 | 0 | 6.8 | 5.61713886793501 | 4.24760045641933 | 7.18286373363821 | 9.7835717179542 | 8.09997586127755 | 5.98222418620833 | -2.221632188 | High |
| GSM519124 | 0 | 7.1 | 5.12965662840046 | 5.26410041967286 | 11.6430291614661 | 8.38823618244805 | 9.98229858112743 | 8.589062817 | -2.729486943 | Low |
| GSM519125 | 0 | 6.5 | 5.56985048900734 | 7.07262850344365 | 12.6882273222244 | 7.75111026657475 | 11.5236227327918 | 8.99792563840165 | -2.903728378 | Low |
| GSM519126 | 1 | 5.4 | 4.96269614057839 | 6.25435014273888 | 11.8775493806505 | 6.64696714585844 | 9.97747870241674 | 10.5697584316731 | -2.980786925 | Low |
| GSM519127 | 0 | 7.1 | 4.8095844182991 | 7.36778517822938 | 12.5344509850079 | 10.7384885301207 | 9.27175824649915 | 6.96361056686466 | -2.632733296 | Low |
| GSM519128 | 0 | 6.9 | 6.5447593390879 | 4.8604983035932 | 8.81270269664358 | 14.0734547362192 | 7.60123875943816 | 4.02455200576177 | -2.259374724 | High |
| GSM519129 | 0 | 7 | 6.24353161946338 | 5.14172092754158 | 9.52860430307815 | 6.57634380243191 | 9.19617253875149 | 12.1491519105069 | -3.208743096 | Low |
| GSM519130 | 0 | 6.4 | 6.59422556105032 | 5.35801899326747 | 13.6460465062782 | 6.99899115980911 | 10.1799109122432 | 9.84187077081225 | -3.093773383 | Low |
| GSM519131 | 0 | 7 | 8.51338675974011 | 5.61326659205413 | 6.1819610992923 | 15.2484667111119 | 8.44701491676729 | 5.62192013292808 | -2.642219748 | Low |
| GSM519132 | 0 | 8 | 7.57379079578262 | 5.01696458380463 | 14.2836780180618 | 11.8512239430409 | 8.75075879010628 | 6.98910220357601 | -2.955259359 | Low |
| GSM519133 | 1 | 1.8 | 6.12823369970801 | 5.69387795560023 | 12.1945429837456 | 8.15208519369852 | 8.63135534565025 | 6.1623899691165 | -2.486705853 | High |
| GSM519134 | 0 | 6.1 | 6.71003830340738 | 5.71707608840465 | 7.6709958794925 | 14.3733814521266 | 6.98076569267896 | 5.03301083252497 | -2.410086506 | High |
| GSM519135 | 1 | 3.5 | 5.67313095849341 | 4.27256461349141 | 7.23812705150501 | 12.1184991880683 | 8.06108411443286 | 6.3633705913958 | -2.372309426 | High |
| GSM519136 | 0 | 7 | 5.84201667215341 | 6.09866202318987 | 10.8057399843705 | 15.0063276152283 | 10.3524962394313 | 7.91289967156474 | -2.927215728 | Low |
| GSM519137 | 0 | 7.2 | 6.08903289522528 | 2.92950393499092 | 9.99258657866289 | 6.59628349216397 | 8.84314958851265 | 4.94627031762673 | -2.080691249 | High |
| GSM519138 | 1 | 5.2 | 7.06097586048048 | 2.15511058901393 | 4.94867326902922 | 14.7265371060099 | 7.4601364152292 | 5.39261783706639 | -2.304804322 | High |
| GSM519139 | 0 | 7.1 | 6.80859357341341 | 4.65540927601193 | 8.11056966535549 | 11.952301876276 | 8.47846562168247 | 6.81234941781106 | -2.59028166 | Low |
| GSM519140 | 0 | 6.8 | 6.6713606523892 | 7.13663144951773 | 10.2171284480381 | 12.8140538093037 | 11.2397286796884 | 8.64852146886303 | -3.041069074 | Low |
| GSM519141 | 1 | 1.9 | 6.87586581874385 | 6.04018608503061 | 9.32798577978747 | 8.18485678801904 | 8.18354523856984 | 6.38263118242527 | -2.481131917 | High |
| GSM519142 | 0 | 6.9 | 4.96870746249748 | 1.62571741402529 | 9.52491267748371 | 7.26597309139896 | 8.3334335694941 | 3.61428871371341 | -1.74360195 | High |
| GSM519143 | 0 | 5.4 | 7.17903707604151 | 5.46758228672659 | 9.5551807266963 | 11.7622853949471 | 9.03101478311404 | 7.26535533067651 | -2.769479424 | Low |
| GSM519144 | 1 | 1.2 | 2.31772209691946 | 1.82971286944995 | 8.71336392434677 | 7.33904027034003 | 8.56638250652016 | 5.43981080987726 | -1.73438955 | High |
| GSM519145 | 1 | 5.8 | 6.56729549664372 | 6.02508972805374 | 13.0911326107001 | 13.1135605 | 7.95995643273722 | 5.16179188486538 | -2.606279248 | Low |
| GSM519146 | 0 | 6.9 | 7.40876241010384 | 5.73235085968968 | 6.57794215377189 | 14.0461075182752 | 7.73170126125481 | 5.92229309636753 | -2.554503092 | High |
| GSM519147 | 0 | 6.5 | 9.25230819061603 | 5.40563348468853 | 7.80369753104143 | 9.13793561161885 | 8.07587024423115 | 6.92961996670864 | -2.746716198 | Low |
| GSM519148 | 0 | 6.9 | 7.69053752434464 | 2.61868957008453 | 7.18229393185516 | 13.0847606993929 | 6.69544092164159 | 4.57866213482761 | -2.28805398 | High |
| GSM519149 | 1 | 4 | 7.17282042601254 | 5.91919329246627 | 7.46456712022135 | 12.3493429727572 | 8.35494903802216 | 3.25682528529202 | -2.115049998 | High |
| GSM519150 | 0 | 6.7 | 7.0314506298181 | 0.84849522909992 | 9.91636723093812 | 11.8577786775648 | 8.03300058991133 | 6.08989797984206 | -2.476014722 | High |
| GSM519151 | 0 | 6 | 5.58100243861514 | 4.36626717500286 | 11.1321940704098 | 8.27131814555042 | 9.60068096372852 | 7.56641865738716 | -2.570644602 | Low |
| GSM519152 | 0 | 6 | 5.45038414819154 | 4.86986129801553 | 10.2826634145026 | 6.63676634639162 | 9.1092155038056 | 7.42793740865385 | -2.454061401 | High |
| GSM519153 | 1 | 3.7 | 7.09543863301295 | 5.46320390356089 | 7.49736669583189 | 13.9530002687101 | 8.44870691678258 | 5.69878721200343 | -2.520402032 | High |
| GSM519154 | 1 | 0.8 | 3.86985194067212 | 6.45204831724258 | 12.1088862240235 | 6.20354003653706 | 9.25440612822532 | 6.0231350103255 | -2.193808795 | High |
| GSM519155 | 0 | 6.7 | 6.75116972389843 | 6.91579174246364 | 13.5928931522643 | 11.4679072881227 | 12.3358803916046 | 9.94918707190338 | -3.331373586 | Low |
| GSM519156 | 0 | 5.8 | 8.2583094893842 | 3.69738791631506 | 12.7377521136155 | 14.9636587794114 | 7.56924077089333 | 3.68359995510782 | -2.540574462 | High |
| GSM519157 | 0 | 6 | 6.86714542430547 | 4.83453230171119 | 12.1199300755138 | 9.68783255207714 | 7.3449345837226 | 5.83922539086652 | -2.537966467 | High |
| GSM519158 | 0 | 6.3 | 6.64149348813941 | 7.23978831450495 | 10.9395871734939 | 10.796638095456 | 10.0403570831281 | 7.74406879703945 | -2.859810455 | Low |
| GSM519159 | 0 | 6.4 | 6.76106310237792 | 7.1146237646042 | 10.5878440077221 | 10.2750740004446 | 9.90891034313552 | 7.12615590257515 | -2.74179776 | Low |
| GSM519160 | 0 | 5.1 | 7.54109445149071 | 5.15652965260917 | 15.381676347473 | 14.1921377265304 | 10.1259669984065 | 7.93709412294613 | -3.231700616 | Low |
| GSM519161 | 0 | 6.4 | 6.16465966072403 | 6.85613996381543 | 13.1499563092288 | 10.8287203393361 | 11.2021590976156 | 8.74265557841399 | -3.048832831 | Low |
| GSM519162 | 0 | 5.9 | 7.50823657847588 | 5.98239952383469 | 9.58715901156038 | 11.4575512587768 | 9.00207034358736 | 6.5289601797852 | -2.694927519 | Low |
| GSM519163 | 0 | 5.4 | 6.63973416230553 | 5.60992167654829 | 8.85528023232526 | 14.0156598780234 | 8.02123773290529 | 5.89805190477496 | -2.568191752 | Low |
| GSM519164 | 0 | 5.9 | 4.75679522555818 | 5.32672840687123 | 5.434463255 | 8.8531716970203 | 8.46853687430544 | 6.10395564014658 | -2.078290715 | High |
| GSM519165 | 0 | 6.4 | 3.8704567225096 | 6.04166802736123 | 10.3106861803945 | 6.81254922745835 | 9.13573352952341 | 7.36229512982697 | -2.329811552 | High |
| GSM519166 | 1 | 2.3 | 6.89709102931505 | 5.50563220195239 | 8.10345695932928 | 14.3388708752957 | 8.6853423485186 | 6.94683319131131 | -2.728576204 | Low |
| GSM519167 | 0 | 7 | 7.79537986148569 | 6.04436687058627 | 10.6596617612383 | 8.70711640871804 | 9.453025292 | 6.29692316004601 | -2.634753142 | Low |
| GSM519168 | 0 | 6.5 | 6.90075938845801 | 3.27575440243837 | 7.55910207982296 | 14.6833116472097 | 7.30170350420304 | 5.50316036053231 | -2.442381694 | High |
| GSM519169 | 0 | 6 | 8.61958754013707 | 6.12041172556151 | 7.98403316231281 | 14.2506346872087 | 7.97912896060297 | 6.01425700899239 | -2.762179916 | Low |
| GSM519170 | 0 | 6.5 | 7.04260043675822 | 6.09078388836573 | 11.2533823794546 | 12.0342976881032 | 10.0837871594846 | 7.84379458703997 | -2.942052818 | Low |
| GSM519171 | 0 | 6.9 | 5.30906544686735 | 7.12969991928123 | 10.2893870055711 | 9.41541143995348 | 9.43760473240946 | 7.26114695134797 | -2.577107608 | Low |
| GSM519172 | 1 | 1.8 | 6.52219936626717 | 5.40790853650476 | 11.8403970425398 | 6.80133084979844 | 8.18170699802411 | 6.86234254142074 | -2.556569183 | High |
| GSM519173 | 0 | 6.8 | 6.92236166812814 | 6.2993326446263 | 10.5077018007093 | 12.9918628152658 | 10.5517005523356 | 7.37028317805688 | -2.870015216 | Low |
| GSM519174 | 0 | 6.8 | 7.23869595792076 | 7.23019528573882 | 13.2100836422561 | 10.1548815080175 | 11.5983888839433 | 8.90862293699971 | -3.165772206 | Low |
| GSM519175 | 0 | 5.3 | 7.30395906486874 | 4.23861594093921 | 8.3619251620282 | 11.9039205518298 | 9.17418487658752 | 6.80282454 | -2.636032681 | Low |
| GSM519176 | 0 | 5.7 | 4.64022761280902 | 5.2135586669341 | 6.84497626201584 | 10.4920177229783 | 7.8008357348167 | 6.77426580987731 | -2.281801009 | High |
| GSM519177 | 0 | 6.6 | 6.52438968084015 | 6.84329733924412 | 10.1614903830181 | 6.32552907801677 | 8.76603595405856 | 6.77938735733114 | -2.495300055 | High |
| GSM519178 | 0 | 5.2 | 7.72700046867506 | 5.23982857756545 | 9.20004765949962 | 14.6915992422217 | 8.40709404063907 | 6.4234610497443 | -2.781980091 | Low |
| GSM519179 | 0 | 4.9 | 8.05164680818878 | 6.43010330184657 | 12.4023247882933 | 11.2611825462003 | 9.15472797947326 | 6.78777604676175 | -2.9086787 | Low |
| GSM519180 | 0 | 5.1 | 6.37247938333978 | 6.72631608212244 | 11.7632949534055 | 11.360420338002 | 10.7094628242163 | 9.18182701407397 | -3.091490087 | Low |
| GSM519181 | 0 | 6.1 | 5.58235785416191 | 7.04207908968243 | 10.3152245261359 | 7.05932829693367 | 9.20194522888417 | 6.49004344158979 | -2.40047769 | High |
| GSM519182 | 0 | 9.2 | 8.12789649657148 | 5.23852092290403 | 8.22623860176802 | 13.0754062455343 | 6.18576314334279 | 6.41914838302756 | -2.715553435 | Low |
| GSM519183 | 0 | 6.5 | 8.7202291437519 | 5.03434229708788 | 5.07018045960604 | 10.8630607197945 | 7.35132712911364 | 6.94616994108572 | -2.635375784 | Low |
| GSM519184 | 0 | 5 | 6.53462065701738 | 6.33685008286555 | 10.0681183739904 | 6.15181567504157 | 10.0154523456391 | 7.44250363820611 | -2.574333758 | Low |
| GSM519185 | 0 | 6.1 | 8.45529018687442 | 1.86798836209941 | 14.5940574979641 | 14.2214046866418 | 7.61498231962283 | 5.50500569733907 | -2.834363283 | Low |
| GSM519186 | 0 | 6.6 | 5.77115817018339 | 6.37600912442011 | 12.4475416363281 | 9.3519567895675 | 11.4765586936578 | 9.48597613792461 | -3.026310812 | Low |
| GSM519187 | 0 | 6.5 | 7.44118321380513 | 5.41040950613131 | 11.5244922236571 | 13.4935102655057 | 10.7415523715634 | 7.79881386817999 | -3.022249448 | Low |
| GSM519188 | 0 | 6.8 | 2.44778607977122 | 6.42645033560782 | 5.6530156127113 | 7.00659020483675 | 8.87296789230162 | 6.03669631466177 | -1.81616685 | High |
| GSM519189 | 0 | 6.4 | 8.18817064044827 | 5.2953116628531 | 8.94533384603918 | 12.3747203360199 | 8.90150470086694 | 7.30170350420304 | -2.864553121 | Low |
| GSM519190 | 1 | 0.9 | 4.18087507216774 | 4.33343465015671 | 13.7191608788868 | 9.20020182455933 | 8.0291955420821 | 6.45942744614251 | -2.408396741 | High |
| GSM519191 | 0 | 6.4 | 4.77252748773417 | 6.83822944183933 | 13.4662869311733 | 6.58864951373283 | 10.8934880418417 | 9.12115810916192 | -2.827684891 | Low |
| GSM519192 | 0 | 6.5 | 7.44993884155375 | 6.03669631466177 | 12.5363839213381 | 13.9041678141385 | 9.3349868 | 6.54919210537201 | -2.90744949 | Low |
| GSM519193 | 1 | 4 | 6.53067987164121 | 6.40767989131947 | 11.9564645425173 | 6.67009538528534 | 10.1664076067808 | 7.52060915935417 | -2.685783406 | Low |
| GSM519194 | 0 | 10.6 | 6.21124036792651 | 6.56963862024955 | 13.2768484530948 | 5.66659556192919 | 9.79007226649845 | 12.4101176797934 | -3.406567437 | Low |
| GSM519195 | 1 | 14.1 | 6.19992836709454 | 5.00000496993575 | 6.63588221369567 | 12.5029162137185 | 7.81553030679574 | 6.57572115707675 | -2.462220941 | High |
| GSM519196 | 1 | 2.3 | 5.88629023568908 | 6.80729752465933 | 13.3541755987644 | 6.76292799744022 | 12.7169253353216 | 9.98646086601259 | -3.069037578 | Low |
| GSM519197 | 0 | 13.8 | 6.78352950418316 | 6.05949399058818 | 9.70676165376144 | 8.59822459369175 | 8.96153518332057 | 7.068239533 | -2.60761037 | Low |
| GSM519198 | 0 | 13.8 | 4.33551259653685 | 6.90012045413113 | 8.07455760370329 | 14.8251498535396 | 9.83263915805277 | 6.35982392594951 | -2.448753976 | High |
| GSM519199 | 0 | 14.1 | 8.46475811726047 | 5.9100894128468 | 9.28760915408714 | 12.1682989842283 | 8.59508602866465 | 5.60545269935524 | -2.660301803 | Low |
| GSM519200 | 1 | 4.3 | 5.7730086597647 | 4.18157056530237 | 7.70360134860968 | 9.0550831633129 | 8.25138453435583 | 6.55524314284128 | -2.315914521 | High |
| GSM519201 | 0 | 13.2 | 4.82386967390432 | 7.10206357133598 | 12.5839785160504 | 12.815402544335 | 10.6661287185058 | 7.40690855954579 | -2.774321323 | Low |
| GSM519202 | 1 | 4.7 | 8.0595954544337 | 6.52008166163205 | 8.20116826985919 | 13.582883881085 | 8.50373466818159 | 6.51229422979573 | -2.778519624 | Low |
| GSM519203 | 1 | 9 | 6.39442415081296 | 4.79640886421953 | 7.90712121929031 | 14.451438105156 | 7.82549139478551 | 6.70766457299252 | -2.619982839 | Low |
| GSM519204 | 1 | 2.9 | 5.89262576514403 | 6.52630235781902 | 12.314638383573 | 5.51213188770174 | 11.0665767217318 | 8.0940096932915 | -2.687223571 | Low |
| GSM519205 | 0 | 11.6 | 7.02321688006728 | 3.60968524964044 | 13.7975565618172 | 14.0011179958395 | 7.95166202226373 | 4.00341704201513 | -2.476576039 | High |
| GSM519206 | 0 | 11.5 | 6.59602315057797 | 5.18948922324534 | 8.93564958997964 | 14.5644308186953 | 7.89671516203413 | 5.16603219037215 | -2.466976448 | High |
| GSM519207 | 0 | 13 | 5.19672473194916 | 7.81477362349789 | 12.4840684993699 | 12.0498503213214 | 10.2059375559583 | 7.49272686091607 | -2.8085691 | Low |
| GSM519208 | 0 | 13.1 | 7.1059198585997 | 4.85410423231357 | 7.69757950533695 | 15.3298103793667 | 8.0021557251683 | 5.71357761335408 | -2.565635879 | Low |
| GSM519209 | 0 | 12.4 | 7.49035641996194 | 5.91374921855173 | 9.51316529411552 | 11.8815700226476 | 8.3881057111212 | 4.48581630263764 | -2.397681971 | High |
| GSM519210 | 1 | 0.8 | 6.3464430176562 | 6.24707367074206 | 9.47747054025738 | 14.3604196493601 | 9.74674849863652 | 7.16398793380773 | -2.787570057 | Low |
| GSM519211 | 0 | 11.6 | 5.92821336759191 | 6.23205064391983 | 9.65031743582015 | 13.0112827532504 | 8.36502039054017 | 5.95142347132239 | -2.520821739 | High |
| GSM519212 | 1 | 6.3 | 6.80829128825933 | 5.65896665440798 | 14.4604247735168 | 11.6139747453709 | 9.68975412586272 | 7.35493495776947 | -2.953249085 | Low |
| GSM519213 | 1 | 4.9 | 7.54570444042075 | 5.29321913229275 | 5.90367620350532 | 14.4208748666648 | 7.39481276689891 | 6.56051890050335 | -2.636348346 | Low |
| GSM519214 | 1 | 8.3 | 6.82624397461477 | 5.41225510054842 | 7.84794715770442 | 15.586266213119 | 7.39550360334913 | 6.04211217992908 | -2.616631743 | Low |
| GSM519215 | 0 | 13.3 | 5.55055673798172 | 6.74821635416991 | 12.5146761365156 | 8.79412420715098 | 9.9244611459993 | 7.21033497213513 | -2.654305601 | Low |
| GSM519216 | 0 | 12.2 | 5.83855873643977 | 7.61542008442832 | 13.6245610923305 | 13.6272346417104 | 12.3210020419372 | 10.039414880776 | -3.355260947 | Low |
| GSM519217 | 1 | 1.3 | 6.00102394392044 | 5.08523882525856 | 9.2433560550447 | 6.47718690694926 | 8.62848384148386 | 5.71514718390851 | -2.206942949 | High |
| GSM519218 | 0 | 12.2 | 5.10864295254549 | 5.21952691188566 | 10.7757693459456 | 6.32078084568515 | 8.2486605651313 | 6.54686392934314 | -2.305979225 | High |
| GSM519219 | 0 | 8.1 | 6.15228754375807 | 5.68628120117111 | 10.4280109254884 | 13.6819342128436 | 9.79510989516906 | 7.4146228708104 | -2.806641006 | Low |
| GSM519220 | 0 | 11 | 6.11476820509083 | 5.35296783100277 | 6.92923490656383 | 11.9648374180024 | 8.22230356775336 | 6.04635767301335 | -2.377597728 | High |
| GSM519221 | 0 | 12.2 | 7.022541126 | 4.98625134055005 | 15.3239921343924 | 13.1482427050659 | 8.93720464332829 | 4.35599101959559 | -2.599671577 | Low |
| GSM519222 | 1 | 5 | 7.35103679802234 | 5.42886552512867 | 9.6981930172811 | 14.7659392651294 | 7.52796053460994 | 5.2356399235234 | -2.595095202 | Low |
| GSM519223 | 1 | 1.4 | 6.96613210158392 | 2.87603801930035 | 4.70656960386346 | 12.9831088609074 | 7.04299572944933 | 6.55037888864892 | -2.412373396 | High |
| GSM519224 | 1 | 6.5 | 5.60538968141445 | 5.37529568730954 | 10.2517817405452 | 13.0231058864437 | 10.4309130924779 | 8.11361044819318 | -2.819804834 | Low |
| GSM519225 | 1 | 5.7 | 5.4028686788607 | 6.98920648861551 | 11.5951804488288 | 6.94025261046357 | 9.05280686427558 | 6.48338884994781 | -2.429675065 | High |
| GSM519226 | 0 | 9.8 | 6.69729312252262 | 5.04749908148498 | 6.88547921142489 | 11.9039205518298 | 8.21201761938105 | 5.63530062276064 | -2.360494945 | High |
| GSM519227 | 0 | 11.6 | 8.03707569324661 | 5.84027707709486 | 6.52087536335933 | 13.8594956199085 | 7.73493347880146 | 5.29304189431827 | -2.514902403 | High |
| GSM519228 | 0 | 12.1 | 8.16083196339196 | 3.51582460820386 | 6.00548856379356 | 10.426489686479 | 9.55401090716513 | 4.22512741490457 | -2.163350923 | High |
| GSM519229 | 1 | 9.2 | 4.90725567804644 | 7.14459839998837 | 9.62206673022804 | 10.9950736453814 | 9.70609281262646 | 6.57115059835181 | -2.466164966 | High |
| GSM519230 | 0 | 12.9 | 4.98367740208277 | 8.03344094532833 | 12.3780930589953 | 11.9941985173398 | 12.0395939106288 | 8.22814970518685 | -2.900704633 | Low |
| GSM519231 | 0 | 12.7 | 5.82420488899685 | 5.21553590792301 | 10.2189497655543 | 8.67861924804521 | 10.1237835811505 | 8.52950438247651 | -2.737755135 | Low |
| GSM519232 | 0 | 12.1 | 3.80370526461606 | 6.0175878627738 | 10.4325091740739 | 13.9358039657433 | 9.67690007466291 | 7.39993113448736 | -2.595404503 | Low |
| GSM519233 | 0 | 12.8 | 6.2132391889427 | 4.57445188866534 | 7.05391105388861 | 11.991744169189 | 7.89287312804252 | 4.7993542960291 | -2.186262158 | High |
| GSM519234 | 0 | 10.9 | 6.62069772620237 | 4.88497644969969 | 11.2890204819472 | 5.78459168131294 | 8.96350654452426 | 7.48588869489507 | -2.586799223 | Low |
| GSM519235 | 1 | 2.5 | 6.86447090775204 | 5.15291344042962 | 11.8679326284795 | 7.00972843702972 | 8.19248571437779 | 6.78177287274309 | -2.579777183 | Low |
| GSM519236 | 1 | 2 | 4.20971099012178 | 5.79613808765765 | 12.3458840467779 | 8.67680043100604 | 8.58761060536992 | 6.82014943966771 | -2.42762207 | High |
| GSM519237 | 1 | 1.2 | 6.37385410552477 | 5.41561941676009 | 6.1226196293045 | 6.83492912672244 | 7.56600907541671 | 6.43010330184657 | -2.238573391 | High |
| GSM519238 | 1 | 11.4 | 5.72154824996905 | 6.55918272688253 | 9.54471160635451 | 14.6280890981592 | 8.67269749847106 | 6.33786753138318 | -2.622462901 | Low |
| GSM519239 | 1 | 4.6 | 4.09660961964609 | 6.53883369699178 | 10.4053776078669 | 12.9553016752393 | 8.6897537090892 | 6.49124791606808 | -2.462624043 | High |
| GSM519240 | 0 | 12.3 | 6.91880546682034 | 6.29404992053218 | 10.5656979714914 | 13.421089790379 | 9.99614563120777 | 7.51296237505567 | -2.907836859 | Low |
| GSM519241 | 0 | 11 | 5.86578941798225 | 5.29486065218444 | 11.2038992211349 | 13.6819342128436 | 9.41634168109696 | 7.06157080562135 | -2.747775636 | Low |
| GSM519242 | 0 | 11.1 | 7.51398654582318 | 4.76986238887301 | 8.63043798568055 | 11.4620770075825 | 8.65647000608183 | 6.75743566835555 | -2.657396694 | Low |
| GSM519243 | 0 | 10.8 | 6.68140518185166 | 5.56638866943703 | 11.602998051663 | 10.0248160082037 | 8.935189852 | 6.91896154317756 | -2.694175113 | Low |
| GSM519244 | 0 | 11 | 6.1929212692014 | 6.21581348781829 | 11.2175829684251 | 10.9398690355643 | 7.98433187380181 | 4.90979864334686 | -2.379500054 | High |
| GSM519245 | 1 | 7 | 6.81076402701956 | 5.53168782478314 | 10.694138283857 | 14.5858410312508 | 7.60294178325324 | 4.64593204190864 | -2.492815196 | High |
| GSM519246 | 1 | 8.9 | 7.38859887648706 | 6.29652394414674 | 12.5566822064079 | 14.4731868957961 | 8.7407106451487 | 7.05231066152693 | -3.003847798 | Low |
| GSM519247 | 0 | 12.6 | 6.36045726408842 | 4.74964730794546 | 11.0531370808402 | 13.4888534746043 | 7.87486466196523 | 7.10815234074066 | -2.771784356 | Low |
| GSM519248 | 1 | 5.9 | 5.54306267279098 | 7.02760988628402 | 8.08142632293974 | 14.9636587794114 | 8.44995119351324 | 4.88135760215715 | -2.350648167 | High |
| GSM519249 | 0 | 11.2 | 5.72982163026771 | 5.39593786370675 | 13.2981702510101 | 7.15046369794376 | 10.6380752920703 | 8.88824529321458 | -2.860851847 | Low |
| GSM519250 | 0 | 11 | 7.72184756670954 | 2.10280212624899 | 7.13649901553855 | 11.4794074948685 | 7.70743423679173 | 3.08852317418205 | -1.996622117 | High |
| GSM519251 | 0 | 11.3 | 6.02301113941864 | 5.44175320798215 | 10.7389956043771 | 6.50753667386441 | 10.4502389730729 | 8.34581519402228 | -2.678377384 | Low |
| GSM519252 | 0 | 11.6 | 8.75708783641768 | 5.60778652096582 | 8.49783259398618 | 12.9918628152658 | 8.82741852683577 | 6.626417912 | -2.830606177 | Low |
| GSM519253 | 1 | 2.4 | 4.39523267605877 | 2.31981345718633 | 8.29965183196713 | 10.0671184260859 | 8.54741768681705 | 7.03068021360381 | -2.267844991 | High |
| GSM519254 | 0 | 11.9 | 7.36974530923801 | 5.06059208440314 | 8.90071832415614 | 10.8334688386803 | 8.24015455635827 | 6.40688855919186 | -2.586166096 | Low |
| GSM519255 | 0 | 12.7 | 7.5703628841185 | 5.03470637875365 | 13.660079668443 | 13.5200302416123 | 7.96395528263829 | 6.44923231186471 | -2.908224672 | Low |
| GSM519256 | 0 | 12.8 | 7.20065666661821 | 3.5023627210874 | 12.006123167422 | 14.6957269876573 | 7.49945788487032 | 5.6478151392919 | -2.685849655 | Low |
| GSM519257 | 1 | 4.6 | 5.93964965589033 | 7.11188061035599 | 12.0191887385486 | 10.5012115786501 | 9.70115572709386 | 7.0383339692432 | -2.717024153 | Low |
| GSM519258 | 0 | 10.9 | 7.46373495698638 | 5.25520528195722 | 6.53387392066577 | 10.5529187677659 | 7.02049814087556 | 5.25914579595789 | -2.317201793 | High |
| GSM519259 | 1 | 11.4 | 6.08370098677609 | 4.49029952092848 | 6.47630661456689 | 11.2059195071914 | 7.87977076641681 | 5.75651467844458 | -2.261413177 | High |
| GSM519260 | 0 | 12.5 | 8.23243791428073 | 5.99319948734832 | 12.8759874782328 | 12.4109676552633 | 8.79633305726369 | 6.60634264132627 | -2.948884437 | Low |
| GSM519261 | 0 | 12.5 | 8.01729112632827 | 3.35932612883707 | 8.60597414900101 | 14.2300334568859 | 8.00230384656833 | 6.16203934927275 | -2.679417594 | Low |
| GSM519262 | 0 | 9.9 | 6.55865207344411 | 4.99565656027609 | 12.6521992228334 | 9.00555080921455 | 11.3623369442032 | 9.13135362731767 | -3.009156007 | Low |
| GSM519263 | 1 | 2.9 | 5.40611406840143 | 5.84157124174892 | 10.6678697675258 | 7.75528994335897 | 9.43335471350972 | 7.17728296618708 | -2.495444393 | High |
| GSM519264 | 0 | 11.1 | 5.77101989660799 | 6.97766306111941 | 12.1178119022212 | 7.19308290332611 | 10.4169257226853 | 7.81242179596307 | -2.697202478 | Low |
| GSM519265 | 1 | 5.7 | 6.9273249111442 | 3.40579028547636 | 4.95366871588128 | 9.20964783331743 | 7.45908915154828 | 4.18636173387605 | -1.942650117 | High |
| GSM519266 | 1 | 1 | 6.0823769783981 | 2.95348692224173 | 10.7020667422448 | 10.816486802424 | 8.32829800276612 | 6.65268386514644 | -2.518512398 | High |
| GSM519267 | 0 | 13.1 | 4.308166184 | 5.91124708909201 | 8.68789446525114 | 12.9215801453568 | 8.0085808960941 | 6.3219691970444 | -2.366933003 | High |
| GSM519268 | 0 | 11.1 | 5.15330039526958 | 6.17518263174008 | 13.077008617136 | 8.04576057302603 | 11.568742015584 | 9.02044574100804 | -2.870611862 | Low |
| GSM519269 | 1 | 5.5 | 3.04717136032879 | 5.65378675599068 | 10.9004940702659 | 6.44621703732643 | 10.8329361821248 | 8.14396913716332 | -2.37155059 | High |
| GSM519270 | 0 | 12.1 | 7.61142184326623 | 6.54698414333411 | 8.95818450268878 | 13.5979803566047 | 9.37202132669906 | 6.79079540534548 | -2.811493299 | Low |
| GSM519271 | 0 | 9.4 | 7.50363064029409 | 6.70425737136742 | 11.9360410557103 | 14.9873275227403 | 11.3486003834092 | 7.97780189959508 | -3.161878287 | Low |
| GSM519272 | 0 | 11.1 | 6.78730698177989 | 4.93223728622201 | 14.03094803 | 9.92020665417025 | 8.40022970498347 | 6.61958771507262 | -2.739910222 | Low |
| GSM519273 | 0 | 12.1 | 8.43396119830286 | 4.83402223128173 | 4.34785882769698 | 15.3361301256771 | 8.03139986135769 | 5.68415379191686 | -2.549117628 | High |
| GSM519274 | 0 | 10.8 | 2.42002166812755 | 5.17606520381176 | 10.6522236423895 | 8.85855639955434 | 8.80526866316406 | 8.30259126136719 | -2.395981742 | High |
| GSM519275 | 0 | 10.2 | 6.97805751128278 | 5.74629441438305 | 7.85567439635902 | 11.5384663363154 | 7.64500762572127 | 4.16258396828351 | -2.212122224 | High |
| GSM519276 | 0 | 12.4 | 7.10273236256586 | 4.97285955236855 | 9.92942091825762 | 12.1170602542537 | 8.8867312617173 | 6.93700160137873 | -2.728575693 | Low |
| GSM519277 | 0 | 7.4 | 3.72742609831678 | 5.24053407211073 | 12.1872414429033 | 6.63329261027925 | 10.2735817105753 | 8.68624306632227 | -2.567001288 | Low |
| GSM519278 | 0 | 11.6 | 8.17576994422693 | 4.97096790634789 | 8.31289432630503 | 13.280738734215 | 8.2547547337452 | 6.29370192196014 | -2.70986004 | Low |
| GSM519279 | 1 | 7.5 | 5.69140659766567 | 5.98671306915207 | 7.28116383229461 | 8.25447088764479 | 9.52844721622276 | 6.41979071304909 | -2.290463696 | High |
| GSM519280 | 1 | 2.2 | 6.16145444121033 | 5.77437447592534 | 7.26350924313402 | 14.9636587794114 | 8.51323390933913 | 6.21201175904907 | -2.542211618 | High |
| GSM519281 | 0 | 7.1 | 5.29055816443332 | 1.91239195839723 | 7.3968711662095 | 7.74909249583362 | 8.45679886319313 | 6.0805254308959 | -2.079082706 | High |
| GSM519282 | 0 | 10.2 | 6.38598190891653 | 7.31018503397626 | 12.5754456701377 | 7.24255474993881 | 10.5390844537846 | 7.48220770142148 | -2.737222778 | Low |
| GSM519283 | 0 | 10.3 | 6.90164428543002 | 3.84400779893295 | 14.0654416400525 | 15.7119305205316 | 8.39712744211246 | 4.38547670329457 | -2.602723478 | Low |
| GSM519284 | 0 | 11.8 | 4.93081934123992 | 5.62370999511105 | 8.59012985447648 | 14.267433212534 | 9.14792272007169 | 7.53707160629513 | -2.648454671 | Low |
| GSM519285 | 1 | 11.5 | 7.04649309354053 | 6.71954409194375 | 12.7215129698613 | 10.8123985139946 | 8.73909157708853 | 4.64413826337114 | -2.495278085 | High |
| GSM519286 | 0 | 10.5 | 7.88680777370004 | 5.79048908146447 | 9.19664077128209 | 12.8585081975295 | 8.81452415795678 | 6.7737742683521 | -2.797611473 | Low |
| GSM519287 | 0 | 11.7 | 6.78136434566392 | 6.89471319214697 | 8.98664482775964 | 14.451438105156 | 8.88869366945842 | 6.33216768538627 | -2.703368398 | Low |
| GSM519288 | 0 | 11.7 | 5.78408887233553 | 6.4221346905683 | 9.46348695437539 | 9.32798577978747 | 9.34736709552724 | 6.80050988473152 | -2.497868582 | High |
| GSM519289 | 1 | 1.6 | 6.95681563396652 | 5.15313318338761 | 8.92093408543075 | 10.2270820766979 | 8.45159712400273 | 6.34961135886856 | -2.519205279 | High |
| GSM519290 | 0 | 10 | 8.15754905432478 | 6.35850179147625 | 9.56712564881216 | 13.3315961522313 | 8.65691505002444 | 6.90764992171685 | -2.891060119 | Low |
| GSM519291 | 0 | 11.7 | 5.81269568928105 | 6.68374658371894 | 9.38574214507749 | 12.6927679077419 | 9.78187269247856 | 7.25231481645272 | -2.695723405 | Low |
| GSM519292 | 0 | 12.5 | 6.70125957062536 | 6.56949422701612 | 9.36534322463982 | 13.41679709 | 7.89658797615125 | 4.79466862528407 | -2.433644822 | High |
| GSM519293 | 1 | 1.3 | 4.27454820634355 | 5.60595834820653 | 10.6450095321876 | 8.57777196471857 | 8.84314958851265 | 6.95601558033367 | -2.374908208 | High |
| GSM519294 | 0 | 10.2 | 6.96792328821567 | 6.98681305990197 | 14.8724459722898 | 6.26469668954144 | 10.0527052856821 | 7.83606280403544 | -2.897174863 | Low |
| GSM519295 | 0 | 10.4 | 6.36634306671896 | 6.30958594674658 | 9.24979679518184 | 8.90631463062085 | 9.23211213129305 | 6.85830584036167 | -2.535197064 | High |
| GSM519296 | 0 | 11.2 | 7.16307087454286 | 6.61359170345853 | 10.7102758600552 | 14.0577494688059 | 7.95224964799995 | 6.94826945048675 | -2.880683119 | Low |
| GSM519297 | 0 | 9.8 | 6.28361215611678 | 7.13781289536574 | 11.9832667795304 | 13.1811139478045 | 11.0461786084799 | 8.2278384391874 | -3.027827738 | Low |
| GSM519298 | 1 | 1.2 | 3.15406334158328 | 5.09523149423177 | 11.4189372960035 | 9.13448648520127 | 9.3468940306158 | 6.88143148237482 | -2.296018234 | High |
| GSM519299 | 0 | 9.4 | 4.32223031804806 | 6.36575958564207 | 12.5640312308761 | 6.18382362885932 | 10.5274077899062 | 11.8577786775648 | -3.126921359 | Low |
| GSM519300 | 0 | 11.3 | 6.54523759421957 | 6.94979994157801 | 12.199657015609 | 10.9389698947099 | 10.5032790790126 | 7.81171875797081 | -2.911950544 | Low |
| GSM519301 | 0 | 10 | 5.20523332575898 | 5.91208951408184 | 10.2005635332265 | 7.01713551164915 | 9.10552585025259 | 12.0857648155474 | -3.163120033 | Low |
| GSM519302 | 0 | 11.5 | 6.7138898618898 | 5.37054678637746 | 9.91838578461055 | 6.59791756863042 | 7.94002573307436 | 6.19011832349565 | -2.385405557 | High |
| GSM519303 | 1 | 7.1 | 3.44347552157701 | 5.19494517111476 | 8.64716243973121 | 6.89709102931505 | 9.27635552541777 | 6.35982392594951 | -2.050643162 | High |
| GSM519304 | 1 | 2.1 | 3.9635640975586 | 1.8469072411458 | 7.45184157817092 | 6.41336232257028 | 7.34468897086229 | 4.6456839671961 | -1.686151211 | High |
| GSM519305 | 1 | 1.8 | 7.26512135193993 | 5.2462826298013 | 9.80319460674422 | 9.38344885431955 | 8.3588400227904 | 6.28582707310867 | -2.547748785 | High |
| GSM519306 | 1 | 5.8 | 7.79537986148569 | 4.75741471020543 | 5.93527984726479 | 13.9641338717934 | 7.19922893632491 | 3.26264532159476 | -2.138555878 | High |
| GSM519307 | 0 | 11.3 | 7.52263974104799 | 5.63518533166364 | 12.8679427671305 | 15.3551333034792 | 10.0595678487669 | 8.41417076537146 | -3.250819872 | Low |
| GSM519308 | 0 | 11.1 | 7.6455852740999 | 4.93045509982995 | 9.82957627696364 | 12.7806108171239 | 9.36930911410177 | 7.19988330519956 | -2.84025744 | Low |
| GSM519309 | 1 | 1.6 | 6.78315495511807 | 5.89216737150697 | 8.85690040397338 | 14.2169332634044 | 7.5802510459013 | 6.17094036910318 | -2.636665099 | Low |
| GSM519310 | 1 | 3.1 | 7.23356305693927 | 5.89347971162207 | 9.77922508148896 | 13.7721403091253 | 8.55436369596498 | 6.45495745362931 | -2.746911027 | Low |
| GSM519311 | 0 | 9.5 | 5.88488217048738 | 6.87281501214112 | 14.7797866214155 | 7.37773521888454 | 9.50109417234088 | 7.41554338052254 | -2.762558395 | Low |
| GSM519312 | 0 | 11.1 | 4.67942057486831 | 5.07122741951643 | 10.7400109488713 | 11.3170180449502 | 11.6364919445309 | 10.6624112566327 | -3.063203143 | Low |
| GSM519313 | 0 | 11.4 | 7.19151741483754 | 5.61274448463308 | 9.9938886176636 | 14.0695119675065 | 9.16856235095642 | 6.82770846311789 | -2.812289724 | Low |
| GSM519314 | 0 | 9.4 | 6.04513035927994 | 4.4086502789369 | 10.7138555228343 | 15.3755131059101 | 9.20568191208199 | 7.23412303373559 | -2.808836551 | Low |
| GSM519315 | 0 | 10.4 | 6.09513261142012 | 4.33586890522692 | 10.5202634326834 | 6.85970553964416 | 9.97424676559442 | 6.49733810671378 | -2.38344281 | High |
| GSM519316 | 0 | 11 | 7.42444839573647 | 4.15792293710392 | 9.29980753895876 | 11.2257121643207 | 8.69303841730878 | 6.2556667759449 | -2.577356402 | Low |
| GSM519317 | 0 | 11.1 | 7.25796543577372 | 6.07484793811426 | 7.72457784670731 | 12.0946975102593 | 8.01484179779368 | 5.15019708200148 | -2.410750043 | High |
| GSM519318 | 0 | 6 | 5.73897546953584 | 5.82248249787469 | 9.66586947466639 | 14.03094803 | 8.35378931930858 | 5.16522930529171 | -2.412506286 | High |
| GSM519319 | 0 | 9.7 | 6.95776916089854 | 5.54317793728753 | 13.7101888552185 | 14.8584355246055 | 7.32476501969989 | 4.12761162893807 | -2.565625698 | Low |
| GSM519320 | 0 | 11 | 8.57994279677401 | 5.38483963157185 | 7.08576404610425 | 15.2323680894573 | 8.2977140977258 | 5.81280384488611 | -2.708120544 | Low |
| GSM519321 | 0 | 11.2 | 5.64359324623482 | 6.95022955878624 | 11.3122337070048 | 13.8068928302844 | 10.5016486887019 | 7.82766217095654 | -2.895096676 | Low |
| GSM519322 | 1 | 3.4 | 5.89316240650125 | 6.14247022169088 | 13.5500042936837 | 12.6984677683885 | 11.3195689066446 | 9.51944679409473 | -3.205380624 | Low |
| GSM519323 | 1 | 10.3 | 9.06695544130366 | 5.08388946228415 | 7.51591082473989 | 12.6499058884677 | 6.86740634943193 | 5.89727891300913 | -2.680454632 | Low |
| GSM519324 | 1 | 4.7 | 5.30977372141586 | 5.66669507712993 | 6.18498183454263 | 7.19380376313937 | 7.62736871909989 | 6.5936837539614 | -2.1825933 | High |
| GSM519325 | 0 | 11 | 5.49893937020982 | 6.09429015090545 | 12.7500931227368 | 11.1236957946173 | 9.83227616692644 | 7.39315154558351 | -2.754533499 | Low |
| GSM519326 | 0 | 11.2 | 6.19938289806635 | 4.74093035871227 | 11.3266602527472 | 11.6501866086976 | 6.97189788774056 | 3.00131200380515 | -2.08539101 | High |
| GSM519327 | 1 | 6.2 | 7.48797674202435 | 5.87272359113691 | 9.25736592169162 | 13.8133789630408 | 7.65725882566307 | 3.2507352695959 | -2.270609117 | High |
| GSM519328 | 0 | 7 | 6.14587160072179 | 5.33368782491815 | 9.62091767870495 | 14.9967620120626 | 8.52387980581912 | 6.12141332045003 | -2.615611383 | Low |
| GSM519329 | 0 | 9.1 | 5.55230369292905 | 6.45158095656281 | 9.77606927598965 | 14.7307240755868 | 8.62879502892902 | 5.29634107716194 | -2.461229116 | High |
| GSM519330 | 0 | 10.2 | 10.0698241554105 | 6.02673220691332 | 7.18082411806504 | 13.241047892898 | 9.1070567910163 | 6.24605931230665 | -2.866167505 | Low |
| GSM519331 | 0 | 11.1 | 8.0947471674227 | 5.24590991602969 | 12.3797354655016 | 13.6379681617647 | 9.02223220050434 | 6.67648418274395 | -2.951196964 | Low |
| GSM519332 | 0 | 11 | 6.37385410552477 | 5.88230536723543 | 10.9873288419831 | 13.3419456313817 | 8.37543826377534 | 6.51315549307603 | -2.706688805 | Low |
| GSM519333 | 1 | 7.5 | 7.13639464345412 | 5.92241508826142 | 10.7362034396792 | 14.0120009080859 | 8.43705826352467 | 7.02152433687001 | -2.871427633 | Low |
| GSM519334 | 0 | 9.5 | 8.25740592319427 | 3.84011035525018 | 5.42555818187799 | 14.556257386746 | 7.6806259651828 | 5.60225964332834 | -2.509772873 | High |
| GSM519335 | 0 | 11 | 6.85331210087544 | 5.08021175305305 | 7.22418876560956 | 14.8724459722898 | 8.46620384787811 | 4.84029860221455 | -2.381183493 | High |
| GSM519336 | 0 | 11 | 6.82578628802024 | 5.90145542019034 | 6.23213212381478 | 10.6434869021721 | 8.49294510748331 | 5.33705068005476 | -2.277723919 | High |
| GSM519337 | 1 | 2.7 | 6.55144781547962 | 5.52058174452253 | 9.26313844398019 | 9.8265855169936 | 8.6458205432501 | 6.10883604309213 | -2.453736602 | High |
| GSM519338 | 0 | 10 | 7.10356130487977 | 5.06316048204641 | 15.2484667111119 | 13.3236507032449 | 9.62490427534409 | 7.20213916176214 | -3.038910499 | Low |
| GSM519339 | 0 | 11 | 7.89458802352171 | 5.28447882746496 | 9.910709664 | 7.44151564291366 | 8.41728276438995 | 5.52873393223383 | -2.430087433 | High |
| GSM519340 | 0 | 11 | 8.54332268187348 | 5.08785097683062 | 7.16124513603345 | 15.493878273281 | 7.44969799833419 | 5.44586021761662 | -2.653086344 | Low |
| GSM519341 | 0 | 10.8 | 5.96060443158438 | 7.09728994039505 | 13.450329089996 | 7.69557505881925 | 11.1427968251833 | 9.53880985995584 | -3.051931413 | Low |
| GSM519342 | 1 | 5.5 | 6.72730524266162 | 5.12681958720873 | 10.6130076706979 | 13.4685325017468 | 9.69026751026214 | 7.89417341872389 | -2.918900388 | Low |
| GSM519343 | 0 | 10 | 7.68335854885055 | 5.88608294168185 | 9.09063413058895 | 11.0807589877706 | 8.82741852683577 | 6.64092961827416 | -2.691109598 | Low |
| GSM519344 | 0 | 9.9 | 5.58312680579318 | 6.86915905431822 | 12.7806108171239 | 14.1720252120856 | 10.5714385383516 | 7.42557035326533 | -2.902112447 | Low |
| GSM519345 | 0 | 9.2 | 7.94996201688067 | 4.76093430307693 | 6.91102802319629 | 14.1155319125012 | 7.76826227587609 | 5.41806844540425 | -2.522792889 | High |
| GSM519346 | 0 | 10.6 | 4.43572896426392 | 5.53564092908705 | 11.6463443130311 | 7.25758417996823 | 10.0920005299082 | 7.56724882496231 | -2.475986069 | High |
| GSM519347 | 1 | 10.1 | 6.00896565600518 | 7.45715922272607 | 12.2571651923432 | 12.0803727465973 | 10.4867042176397 | 7.44178003764611 | -2.862320113 | Low |
| GSM519348 | 0 | 10.5 | 5.82902439819999 | 7.6269690191542 | 13.6300278669807 | 7.20844195825768 | 12.3588872503794 | 9.64161413691854 | -3.060530256 | Low |
| GSM519349 | 1 | 1.9 | 6.88046097845989 | 3.97345191521906 | 11.1777869703613 | 13.1516770169131 | 8.29505435448884 | 6.8912414998442 | -2.763159796 | Low |
| GSM519350 | 0 | 9.2 | 6.6052483630236 | 5.57026632473139 | 9.92980130966663 | 14.0882346057544 | 9.27795530663424 | 7.28306754466071 | -2.820718646 | Low |
| GSM519351 | 0 | 7.9 | 7.64679348101381 | 5.44272302090037 | 11.3403473856734 | 15.092898877071 | 7.22282620284864 | 5.36624930416577 | -2.723609558 | Low |
| GSM519352 | 1 | 8.1 | 5.44530430266632 | 6.86848952633001 | 11.7680052874453 | 9.30347138784307 | 9.64673740809999 | 6.4928580160459 | -2.527029602 | High |
| GSM519353 | 0 | 7.5 | 7.5210689598721 | 4.66765182186268 | 8.00230384656833 | 9.69548235877963 | 7.48588869489507 | 6.07508777801481 | -2.460213442 | High |
| GSM519354 | 0 | 9.4 | 6.94989660015936 | 4.82493639748945 | 15.0747409557633 | 15.2211738333386 | 9.35657273384906 | 6.58090772251444 | -2.98681344 | Low |
| GSM519355 | 0 | 10.6 | 7.18229393185516 | 5.9432579994609 | 9.85766403812079 | 12.07571734 | 8.40756722486295 | 6.39473670258886 | -2.6751291 | Low |
| GSM519356 | 0 | 10.2 | 7.32643192474078 | 2.10363857987459 | 14.4121389707812 | 14.4427796965559 | 8.1121306069546 | 6.6568633274624 | -2.904836359 | Low |
| GSM519357 | 1 | 5.3 | 7.6219441150782 | 6.07968498967071 | 8.38648543834315 | 10.2295336949089 | 8.45545502822284 | 5.26026408570456 | -2.422774467 | High |
| GSM519358 | 0 | 8.9 | 7.78747210234128 | 7.22502554960811 | 11.1855286519907 | 13.7911102237707 | 10.8841060348088 | 7.95514514897509 | -3.123323889 | Low |
| GSM519359 | 1 | 1.3 | 5.92263840830026 | 5.7996438881079 | 9.73062267385304 | 10.3102426927912 | 9.10535700349799 | 4.91985298265997 | -2.261034873 | High |
| GSM519360 | 0 | 9 | 6.48991297434204 | 5.13002049684466 | 9.76552137008676 | 15.2373393337441 | 8.84329104891769 | 6.2887835961674 | -2.684037898 | Low |
| GSM519361 | 0 | 8.4 | 6.70855454640283 | 4.91103322537122 | 7.18404574366147 | 14.0918205643322 | 7.5753022606937 | 5.94710029861319 | -2.495873469 | High |
| GSM519362 | 0 | 9.1 | 6.27914149441913 | 3.25932687768225 | 9.24773271504262 | 3.60982398196887 | 7.94864218253297 | 6.48562449530888 | -2.195009314 | High |
| GSM519363 | 0 | 8.7 | 6.0962915023783 | 7.0542588167552 | 12.9787288572071 | 10.6538861580315 | 11.2644921597191 | 9.05093638623788 | -3.079953267 | Low |
| GSM519364 | 0 | 10.3 | 6.65398596088996 | 5.18086222600089 | 6.85724747239138 | 9.43487117862697 | 7.54316978013479 | 2.07804704910321 | -1.735962647 | High |
| GSM519365 | 0 | 9.6 | 5.21671432786303 | 5.68736958127368 | 7.51231435435123 | 6.95979599201468 | 7.92070265864011 | 5.39629403696344 | -2.043039322 | High |
| GSM519366 | 0 | 10.1 | 6.97934118799676 | 6.82115070142148 | 9.81925153737395 | 14.5214813194427 | 9.24724109896754 | 5.05988288436617 | -2.56886137 | Low |
| GSM519367 | 1 | 3.7 | 6.60247379475606 | 4.23971602734081 | 7.53333627323278 | 6.27267194464453 | 7.53263619539155 | 5.64532432817557 | -2.151297409 | High |
| GSM519368 | 1 | 3.4 | 7.80426294935632 | 2.90410450734364 | 6.06276153850168 | 8.43453626083984 | 6.95483823343735 | 5.53040007364398 | -2.231891615 | High |
| GSM519369 | 0 | 10.4 | 6.70793264363917 | 5.67016420345558 | 12.7437551103352 | 13.9248634969573 | 9.91693292977198 | 7.13649901553855 | -2.924478206 | Low |
| GSM519370 | 1 | 5 | 5.52132375099245 | 4.5846517276971 | 14.3861901430013 | 11.3985061403658 | 8.13406732312936 | 6.11230053904177 | -2.601289263 | Low |
| GSM519371 | 1 | 7.5 | 8.54963992291618 | 4.75169698037585 | 6.43051552398485 | 12.4023247882933 | 7.3077172119993 | 6.2420161673979 | -2.619663011 | Low |
| GSM519372 | 0 | 10.1 | 5.848096195 | 7.66656611832197 | 12.0763974767665 | 12.5393980392214 | 10.519558686159 | 8.10999454195522 | -2.961263142 | Low |
| GSM519373 | 0 | 7.2 | 6.35888390376367 | 7.34150874692311 | 11.1838033696262 | 8.28268185709639 | 9.94900388698158 | 7.81523823551439 | -2.763752446 | Low |
| GSM519374 | 0 | 10.1 | 7.74166078183289 | 5.53040007364398 | 6.84975882563859 | 14.1035935637956 | 6.9277813697508 | 5.95001768856987 | -2.597382873 | Low |
| GSM519375 | 0 | 8.9 | 6.69788474174629 | 6.0117706423666 | 11.1757015213542 | 13.6432044819452 | 9.75097652769527 | 7.08987505941503 | -2.849163149 | Low |
| GSM519376 | 0 | 9.4 | 6.52008166163205 | 6.86421091278805 | 7.81452605687626 | 14.900580183464 | 7.83984152137204 | 5.89490886310625 | -2.577317247 | Low |
| GSM519377 | 0 | 10.2 | 5.93041662138999 | 6.97316646199768 | 10.1397189970548 | 13.8633486446566 | 9.32337823012684 | 6.81115370516455 | -2.722292034 | Low |
| GSM519378 | 0 | 9.4 | 6.2501577489163 | 5.4220108340775 | 6.89656573168847 | 13.3156984129497 | 8.14881788029122 | 5.78327780895626 | -2.401167957 | High |
| GSM519379 | 0 | 9.2 | 7.56448890741876 | 5.66412026466976 | 8.45560749123316 | 10.47432851 | 7.86849905223297 | 5.53428767230575 | -2.457945979 | High |
| GSM519380 | 0 | 9.2 | 4.49541009935017 | 6.7424602707489 | 12.522502500585 | 8.10287918269339 | 11.483937995839 | 11.4715502464203 | -3.166322547 | Low |
| GSM519381 | 0 | 9.9 | 6.16684278236299 | 6.34666669887709 | 12.7878383072414 | 9.4237015437024 | 10.5447857944477 | 8.44037058260647 | -2.92270575 | Low |
| GSM519382 | 1 | 3.1 | 6.46042216916897 | 5.49854191448916 | 9.03964019573336 | 8.62090217406198 | 8.20319637018719 | 2.04156963889806 | -1.78298589 | High |
| GSM519383 | 0 | 9.8 | 8.57424144618968 | 3.93866267673009 | 15.1773217805082 | 15.3361301256771 | 8.72401067287008 | 6.54168775725105 | -3.122021505 | Low |
| GSM519384 | 0 | 8.3 | 5.79576686383482 | 6.44714115553048 | 12.4971848177095 | 9.09033609865376 | 10.2019888683131 | 7.79208854461165 | -2.76765132 | Low |
| GSM519385 | 0 | 9.8 | 6.626417912 | 8.0703057156023 | 12.1886612725155 | 11.5336440520999 | 11.2696396517637 | 8.91987489172689 | -3.137247957 | Low |
| GSM519386 | 0 | 10.4 | 7.25907987583507 | 6.15378617502845 | 14.8251498535396 | 10.9010530887779 | 8.70919143444567 | 5.72419966646359 | -2.753494924 | Low |
| GSM519387 | 0 | 8.9 | 4.86676889464546 | 7.84289377656177 | 13.2391789610303 | 6.09417776509086 | 11.4312536401749 | 7.56794697122871 | -2.60466487 | Low |
| GSM519388 | 0 | 9.5 | 6.63746823073449 | 6.64809721956462 | 13.0098361136044 | 12.3217324587229 | 11.3630873496797 | 8.8834206375279 | -3.159238107 | Low |
| GSM519389 | 0 | 9.5 | 6.218871237 | 4.29575664364857 | 7.04770473167257 | 5.61301732658326 | 10.0459538307428 | 8.88689735330486 | -2.560095821 | Low |
| GSM519390 | 0 | 9.6 | 8.84483001028193 | 4.87400945782175 | 14.1840649654825 | 11.794569407918 | 7.70474370847595 | 2.58034378816934 | -2.408028174 | High |
| GSM519391 | 0 | 9.6 | 3.83225002617113 | 5.57354033726024 | 8.68914497227792 | 14.806544254604 | 9.09496469573346 | 6.81643980718054 | -2.45723944 | High |
| GSM519392 | 0 | 8.3 | 6.00548856379356 | 6.94282637791679 | 11.7685443004965 | 8.01381310963261 | 11.0792296065042 | 8.11787607010376 | -2.781321337 | Low |
| GSM519393 | 0 | 9.4 | 5.15921371190173 | 7.87647020269889 | 13.7101888552185 | 8.19248571437779 | 12.1617980561046 | 8.93396391428168 | -2.935697287 | Low |
| GSM519394 | 0 | 9.4 | 6.16344316782341 | 7.35356313039323 | 13.8803376315257 | 11.7790460343172 | 12.5344509850079 | 10.6591965502595 | -3.415782756 | Low |
| GSM519395 | 0 | 8.6 | 6.8189847949166 | 4.80227982934158 | 15.092898877071 | 14.254793404002 | 9.05960309970468 | 7.16638923300021 | -3.025712665 | Low |
| GSM519396 | 1 | 6.6 | 6.73640465796745 | 4.58736674874322 | 13.9248634969573 | 14.0616742132756 | 9.32019170263886 | 6.45784746217523 | -2.850965264 | Low |
| GSM519397 | 0 | 9.3 | 7.60467876123959 | 1.41339214513548 | 6.77798953633743 | 14.1396006096841 | 6.66730729509417 | 6.06784329338686 | -2.492232724 | High |
| GSM519398 | 1 | 7 | 5.00634711583072 | 2.85098739102094 | 11.0909270769942 | 9.76497484459877 | 8.56961774981763 | 4.10950218751334 | -2.010612522 | High |
| GSM519399 | 0 | 9.1 | 6.05934854410248 | 6.86458513677986 | 12.8401264688818 | 7.23572924052657 | 10.4251580854715 | 7.94659225516589 | -2.77394968 | Low |
| GSM519400 | 1 | 3.3 | 7.86252841078629 | 3.94131754672191 | 8.68207535696614 | 15.1825907771662 | 7.98752343065631 | 5.90535872395353 | -2.679361805 | Low |
| GSM519401 | 0 | 9 | 6.24848768620654 | 6.6659201640376 | 11.7115370744043 | 14.4561685442753 | 10.2230527812449 | 7.1618038751832 | -2.886685679 | Low |
| GSM519402 | 0 | 8.1 | 6.98709032304763 | 6.2577800882538 | 9.82444204731092 | 13.6870994736323 | 9.4912395143507 | 6.61008569825166 | -2.756432732 | Low |
| GSM519403 | 1 | 0.4 | 7.11907052074624 | 1.95519052043844 | 6.05022849728152 | 12.418390725736 | 8.3160529712797 | 6.07076354469171 | -2.369744146 | High |
| GSM519404 | 0 | 9.1 | 4.11332806152694 | 6.46416501780722 | 12.5333943433192 | 9.86964416756291 | 11.3885936174338 | 9.53456379380288 | -2.898050189 | Low |
| GSM519405 | 0 | 8.9 | 5.95881554450636 | 4.8600735998508 | 9.95869457839237 | 14.7265371060099 | 8.66046167252293 | 6.51497046231854 | -2.648450441 | Low |
| GSM519406 | 0 | 8.9 | 6.42488921628059 | 3.73019210739173 | 6.63316936338259 | 11.9607049570681 | 8.65571273804179 | 5.58262561491606 | -2.284413016 | High |
| GSM519407 | 0 | 8.2 | 7.16600970179147 | 4.82513274052157 | 4.66117791163267 | 11.7919021675631 | 7.76240082074337 | 3.84421207699355 | -2.034574075 | High |
| GSM519408 | 0 | 7.7 | 8.6897537090892 | 1.15178924950654 | 13.2709721686203 | 10.618348978719 | 7.72126983971924 | 4.47337240736781 | -2.497295568 | High |
| GSM519409 | 0 | 8.7 | 6.03228081561473 | 4.94287117075251 | 7.96930437802885 | 10.0653768042304 | 8.13243406669025 | 6.40969732450106 | -2.386957944 | High |
| GSM519410 | 0 | 8.1 | 6.42161675987122 | 6.02365001429663 | 12.7661144574101 | 14.7222396240997 | 10.1427957409398 | 6.7276747753926 | -2.875612044 | Low |
| GSM519411 | 0 | 8.6 | 6.24828927201761 | 7.37432003787323 | 12.2286948574173 | 10.1363942928164 | 10.5750263895943 | 7.447212041 | -2.811897283 | Low |
| GSM519412 | 0 | 9 | 5.60381994037094 | 5.23120406346912 | 10.7397564199177 | 11.2376079322559 | 9.22834731536792 | 7.43747148845946 | -2.667580734 | Low |
| GSM519413 | 0 | 7.8 | 6.79861242927303 | 5.60881420421046 | 11.308879670158 | 14.5300492054243 | 10.1871822044362 | 7.12411776568168 | -2.892446794 | Low |
| GSM519414 | 0 | 8.7 | 7.13622452491878 | 5.82951109944701 | 7.78241883027992 | 13.0008122901142 | 8.32530996000368 | 7.00919164051051 | -2.706254675 | Low |
| GSM519415 | 0 | 8.4 | 8.35631381548777 | 5.20491627818901 | 6.31057553520814 | 13.9390342740506 | 7.79395825449338 | 2.84939974294359 | -2.158749408 | High |
| GSM519416 | 0 | 8.3 | 5.0465239083093 | 4.60078205320445 | 11.4794074948685 | 9.16730339064276 | 9.52326545468443 | 6.34049648112535 | -2.389351618 | High |
| GSM519417 | 1 | 2.4 | 5.79211142348454 | 5.57496908855064 | 8.81331267422187 | 10.5695240531921 | 8.2914995593605 | 5.00897502860337 | -2.225304301 | High |
| GSM519418 | 0 | 8.5 | 6.23139962925554 | 7.35466524982829 | 12.2781567521533 | 12.451325321904 | 10.6139810140722 | 7.33199249551825 | -2.879479735 | Low |
| GSM519419 | 1 | 2.9 | 6.26113633321212 | 5.9478452441091 | 6.59686579798879 | 11.2069336575591 | 7.76913556944924 | 5.24996742155182 | -2.245801392 | High |
| GSM519420 | 0 | 8.1 | 6.33338334234625 | 5.61230980162552 | 8.62120240312145 | 10.316307974943 | 8.50358047014481 | 5.79746637647597 | -2.379363616 | High |
| GSM519421 | 0 | 8.1 | 7.30964543581578 | 5.97427897572153 | 11.7384724276847 | 10.4053776078669 | 11.016021351254 | 8.68624306632227 | -3.053099564 | Low |
| GSM519422 | 0 | 8 | 5.88990798855591 | 6.29225955499318 | 12.2206713079035 | 7.46776863211377 | 10.7128176889825 | 7.09728994039505 | -2.599076729 | Low |
| GSM519423 | 0 | 8 | 6.30182644371427 | 5.15384187059311 | 6.89167063139415 | 7.98869892302065 | 7.55034002497174 | 6.28805698555159 | -2.278003392 | High |
| GSM519424 | 0 | 8.4 | 6.26548147051719 | 6.91717977683607 | 11.3232792730827 | 7.74504686235173 | 10.8677233792466 | 8.82244582397048 | -2.882006899 | Low |
| GSM519425 | 1 | 3.5 | 7.25190912338143 | 5.12296469318519 | 13.5298873036933 | 12.2688154700177 | 7.88213348017005 | 4.14482774075479 | -2.484426705 | High |
| GSM519426 | 0 | 8.1 | 8.38545668381884 | 2.11688107008372 | 13.1135605 | 14.2921566535455 | 9.09957521992978 | 6.48116415531644 | -2.92343115 | Low |
| GSM519427 | 0 | 8.5 | 8.56993651652886 | 3.95454174445661 | 7.18364550139125 | 10.9867331048947 | 7.72184756670954 | 5.70917335536637 | -2.501866366 | High |
| GSM519428 | 0 | 8.1 | 7.42992162307846 | 1.58696427132921 | 6.28795114060874 | 12.9430072037181 | 8.20993187807334 | 5.32348425527178 | -2.307609336 | High |
| GSM519429 | 0 | 6.4 | 6.43500428435482 | 2.89209988839084 | 13.1936247882903 | 12.2286948574173 | 10.649795658427 | 7.14152598464251 | -2.784682723 | Low |
| GSM519430 | 0 | 1.5 | 7.72073468707343 | 5.56930384543396 | 7.88142463336461 | 12.5839785160504 | 8.33171062779639 | 6.38514938115365 | -2.651714985 | Low |
| GSM519431 | 1 | 6.8 | 5.74868567645525 | 5.20212849735073 | 12.8558923387934 | 11.5988456135727 | 11.0959572977496 | 8.30626140976464 | -2.916092472 | Low |
| GSM519432 | 0 | 6.7 | 7.05857475112827 | 1.74348713204923 | 6.95979599201468 | 14.7524544795162 | 7.87880143016262 | 6.85904771231924 | -2.598671095 | Low |
| GSM519433 | 0 | 6.5 | 7.34427265043429 | 6.73615376078411 | 10.0182751754975 | 11.4085636493926 | 8.98307430405602 | 6.87158608996166 | -2.766070404 | Low |
| GSM519434 | 1 | 4 | 6.21915824313888 | 5.99763642159484 | 9.47050678274528 | 14.556257386746 | 9.53519811192208 | 7.86293904499616 | -2.879526315 | Low |
| GSM519435 | 0 | 5.8 | 7.71431039717386 | 5.1957749047172 | 6.17822807239973 | 15.1419565931057 | 7.5726703545328 | 4.8626646008429 | -2.434984766 | High |
| GSM519436 | 0 | 5 | 7.03332780220849 | 5.47017477732562 | 10.7128176889825 | 11.9570873828126 | 9.17870069280881 | 7.98491804635984 | -2.918742079 | Low |
| GSM519437 | 0 | 5.3 | 6.68624313719257 | 4.77540155441818 | 6.43636879326542 | 12.0466466419027 | 7.06272730010282 | 5.94225787852588 | -2.382224854 | High |
| GSM519438 | 0 | 4.9 | 7.58644682936529 | 5.44530430266632 | 11.9510022945323 | 12.7513707622789 | 7.24958816910244 | 6.23282310191226 | -2.78703765 | Low |
| GSM519439 | 0 | 4.7 | 4.43700377758749 | 0.791332007165105 | 8.50504687649028 | 12.2407476127831 | 9.63665635089098 | 5.37844681899284 | -2.075903566 | High |
| GSM519440 | 0 | 4.1 | 7.07145690661853 | 0.88825981911021 | 13.2430775883911 | 13.5879592626582 | 9.50721540688185 | 7.24718310233125 | -2.859239254 | Low |
| GSM519441 | 0 | 3.8 | 6.94660895066244 | 5.78693368159626 | 8.13450532162523 | 14.6833116472097 | 9.17342268454352 | 7.19651357508753 | -2.792861377 | Low |
| GSM519442 | 0 | 3.8 | 7.19530227203704 | 5.27189537269718 | 5.30159876974669 | 14.5900457079983 | 7.75585143248454 | 5.94764274831986 | -2.492206536 | High |
| GSM519443 | 0 | 8.1 | 7.01933193422254 | 5.14569539221138 | 12.616928452851 | 14.5858410312508 | 8.86470741222156 | 6.78364849541953 | -2.904988087 | Low |
