# Supplementary material for: Identification of NETs and inflammation-related prognostic genes in breast cancer and PCR experimental validation
Source: Front Genet. 2026 Aug 21;17:1900298. doi: 10.3389/fgene.2026.1900298 (PMC13541293; doi:10.3389/fgene.2026.1900298)
Supplement: Supplementary file 2 [file DataSheet2.zip › Supplementary Table0713/supplementary table 1.docx]

symbol

ABCA1

ABI1

ACVR1B

ACVR2A

ADM

ADORA2B

ADRM1

AHR

APLNR

AQP9

ATP2A2

ATP2B1

ATP2C1

AXL

BDKRB1

BEST1

BST2

BTG2

C3AR1

C5AR1

CALCRL

CCL17

CCL2

CCL20

CCL22

CCL24

CCL5

CCL7

CCR7

CCRL2

CD14

CD40

CD48

CD55

CD69

CD70

CD82

CDKN1A

CHST2

CLEC5A

CMKLR1

CSF1

CSF3

CSF3R

CX3CL1

CXCL10

CXCL11

CXCL6

CXCL9

CXCR6

CYBB

DCBLD2

EBI3

EDN1

EIF2AK2

EMP3

ADGRE1

EREG

F3

FFAR2

FPR1

FZD5

GABBR1

GCH1

GNA15

GNAI3

GP1BA

GPC3

GPR132

GPR183

HAS2

HBEGF

HIF1A

HPN

HRH1

ICAM1

ICAM4

ICOSLG

IFITM1

IFNAR1

IFNGR2

IL10

IL10RA

IL12B

IL15

IL15RA

IL18

IL18R1

IL18RAP

IL1A

IL1B

IL1R1

IL2RB

IL4R

IL6

IL7R

CXCL8

INHBA

IRAK2

IRF1

IRF7

ITGA5

ITGB3

ITGB8

KCNA3

KCNJ2

KCNMB2

KIF1B

KLF6

LAMP3

LCK

LCP2

LDLR

LIF

LPAR1

LTA

LY6E

LYN

MARCO

MEFV

MEP1A

MET

MMP14

MSR1

MXD1

MYC

NAMPT

NDP

NFKB1

NFKBIA

NLRP3

NMI

NMUR1

NOD2

NPFFR2

OLR1

OPRK1

OSM

OSMR

P2RX4

P2RX7

P2RY2

PCDH7

PDE4B

PDPN

PIK3R5

PLAUR

PROK2

PSEN1

PTAFR

PTGER2

PTGER4

PTGIR

PTPRE

PVR

RAF1

RASGRP1

RELA

RGS1

RGS16

RHOG

RIPK2

RNF144B

ROS1

RTP4

SCARF1

SCN1B

SELE

SELL

SELENOS

SEMA4D

SERPINE1

SGMS2

SLAMF1

SLC11A2

SLC1A2

SLC28A2

SLC31A1

SLC31A2

SLC4A4

SLC7A1

SLC7A2

SPHK1

SRI

STAB1

TACR1

TACR3

TAPBP

TIMP1

TLR1

TLR2

TLR3

TNFAIP6

TNFRSF1B

TNFRSF9

TNFSF10

TNFSF15

TNFSF9

TPBG

VIP
